# CarcinoPred-EL: Novel models for predicting the carcinogenicity of chemicals using molecular fingerprints and ensemble learning methods

**DOI:** 10.1038/s41598-017-02365-0

**Published:** 2017-05-18

**Authors:** Li Zhang, Haixin Ai, Wen Chen, Zimo Yin, Huan Hu, Junfeng Zhu, Jian Zhao, Qi Zhao, Hongsheng Liu

**Affiliations:** 1 School of Life Science, Liaoning University, Shenyang, 110036 China; 2 Research Center for Computer Simulating and Information Processing of Bio-macromolecules of Liaoning Province, Shenyang, 110036 China; 3 Engineering Laboratory for Molecular Simulation and Designing of Drug Molecules of Liaoning, Shenyang, 110036 China; 4 School of Information, Liaoning University, Shenyang, 110036 China; 5 School of Mathematics, Liaoning University, Shenyang, 110036 China

## Abstract

Carcinogenicity refers to a highly toxic end point of certain chemicals, and has become an important issue in the drug development process. In this study, three novel ensemble classification models, namely Ensemble SVM, Ensemble RF, and Ensemble XGBoost, were developed to predict carcinogenicity of chemicals using seven types of molecular fingerprints and three machine learning methods based on a dataset containing 1003 diverse compounds with rat carcinogenicity. Among these three models, Ensemble XGBoost is found to be the best, giving an average accuracy of 70.1 ± 2.9%, sensitivity of 67.0 ± 5.0%, and specificity of 73.1 ± 4.4% in five-fold cross-validation and an accuracy of 70.0%, sensitivity of 65.2%, and specificity of 76.5% in external validation. In comparison with some recent methods, the ensemble models outperform some machine learning-based approaches and yield equal accuracy and higher specificity but lower sensitivity than rule-based expert systems. It is also found that the ensemble models could be further improved if more data were available. As an application, the ensemble models are employed to discover potential carcinogens in the DrugBank database. The results indicate that the proposed models are helpful in predicting the carcinogenicity of chemicals. A web server called CarcinoPred-EL has been built for these models (http://ccsipb.lnu.edu.cn/toxicity/CarcinoPred-EL/).

## Introduction

Evaluating the toxicity of new compounds is an essential part of the drug development process^1, 2^. Any chemical substances that can cause cancer are defined as carcinogens. Thus, among various toxicological endpoints of chemical substances, carcinogenicity is of great concern because of its serious effects on human health. The carcinogenic mechanism of chemicals may be due to their ability to damage the genome or disrupt cellular metabolic processes. Many approved drugs have been identified as carcinogens in humans or animals and have been withdrawn from the market^3^. To prevent the appearance of drug-induced cancer, as stipulated by regulatory authorities, pharmaceutical companies must perform several carcinogenicity tests before receiving marketing approval for their new compounds^4^.

In general, the carcinogenic potency of chemicals is evaluated using animal models, such as the 2-year rodent carcinogenicity assay and the 26-week Tg-rasH2 mice carcinogenicity study^5^. However, these animal model experiments are laborious, time consuming, highly expensive, and even unethical. It is impossible to assess the carcinogenicity of a large number of unascertained chemicals to identify problematic compounds in the early stages of drug development. Therefore, computational approaches for predicting carcinogenicity based on chemical structure properties are recognized as an alternative solution, and have become the focus of research in recent years^6, 7^.

Over the past decades, numerous computational approaches have been proposed for the prediction of chemical carcinogenicity. These approaches can be divided into three categories: qualitative structure–activity relationship (SAR) models^8–20^, i.e., classification models, quantitative structure–activity relationship models (QSAR)^19–25^, and expert systems^26–29^. SAR and QSAR models attempt to describe the relationships between chemical structure features (usually represented by molecular descriptors) and biological activity (e.g., carcinogenicity) based on known activity data using various statistical or mathematical methods^30^. Many SAR and QSAR models have achieved high predictive accuracy in specific congeneric chemical classes such as nitrocompounds^21^, aromatic amines^22^, polycyclic aromatic hydrocarbons^23^, and polychlorinated biphenyls^8^. For example, Morales *et al*. developed a QSAR model for predicting the rodent carcinogenicity of nitrocompounds by applying a topological substructural molecular design approach, which gave a determination coefficient of 0.666 in leave-one-out validation^21^. Tanabe *et al*. constructed a series of support vector machine (SVM)-based SAR models using 20 mutually overlapping subgroups of 911 chemicals, and reported an overall classification accuracy of approximately 80%^9^. However, these models can only be applied to a specific group of congeneric chemicals; in other words, they have a limited applicability domain (AD), and are therefore unsuitable for regulatory use, where there is a need to evaluate diverse classes of chemicals.

In recent years, several SAR models for predicting the carcinogenicity of diverse classes of compounds have been developed based on heterogeneous databases^16–20^. For example, Fjodorova *et al*. presented a carcinogenic potency classification model for diverse chemicals that achieved an accuracy of 92.2% on the training set and 68.3% on the test set^20^. Their model was constructed using 27 molecular descriptors and a counter-propagation artificial neural network (CP ANN) technique based on a dataset containing 422 carcinogenic and 383 non-carcinogenic organic compounds^20^. Zhang *et al*. built a naïve Bayes classification model using five simple molecular descriptors and extended-connectivity fingerprints (ECFPs), and achieved an overall accuracy of 90% with an internal training set and 68% in five-fold cross-validation^16^. These models have a wide AD, but their accuracy in forecasting the carcinogenicity of new compounds (the accuracy estimated by cross-validation or external testing) remains unsatisfactory. Moreover, many models that achieve higher accuracy are generated through fine-tuning processes and have not been evaluated by an appropriate cross-validation.

Structural alert-based expert systems also achieve an overall accuracy of about 70% in predicting the carcinogenicity of compounds^27, 31^. This reflects that the carcinogenicity of a compound is closely related to its two-dimensional structure, which means that molecular fingerprints can be used to predict carcinogenicity.

Molecular fingerprints have been widely used in many aspects of computer-aided drug design, such as virtual screening^32^ and similarity search^33^, but are rarely used in the prediction of carcinogenicity. Using six types of fingerprints, Li *et al*. applied 30 classification models, the best of which was generated by MACCS fingerprints and a k-nearest neighbour (kNN) algorithm, with an overall accuracy of 80.46% in an external validation set^17^. However, the accuracy of the kNN model was only estimated using external validation, and did not use cross-validation.

Ensemble learning is a rather new machine learning model building method. Ensemble models can be formed by fusing a series of simple independent models (base classifiers) via voting or averaging. The ensemble learning method typically produces more accurate and robust models than any of its constituent models. On the other hand, it also has some limitations. For example, the computational cost for training and prediction is high, and the resulting model is difficult to interpret. Nevertheless, it has been successfully used in many cheminformatics and bioinformatics applications, such as hepatotoxicity prediction^34^, and phosphorylation sites prediction^35^. In this study, we apply this method to the prediction of carcinogenicity of chemicals.

The aim of the current study is to build classification models using different molecular fingerprints and ensemble machine learning methods to satisfactorily predict the carcinogenicity of diverse organic compounds, and to identify the structural features related to carcinogenic effects. The predictive performance of the models will be carefully evaluated by five-fold cross-validation with 100 repeats and external validation, which is commonly used in the evaluation of computational models^36–41^. The models are expected to be used in the early stages of drug discovery to filter potential carcinogens. For this purpose, a free carcinogenicity prediction online server has been built to enable public access to the models. The web server is called **CarcinoPred-EL** (**Carcino**genicity **Pred**iction using **E**nsemble **L**earning methods).

## Results and Discussion

### Dataset analysis

**Figure 1:**
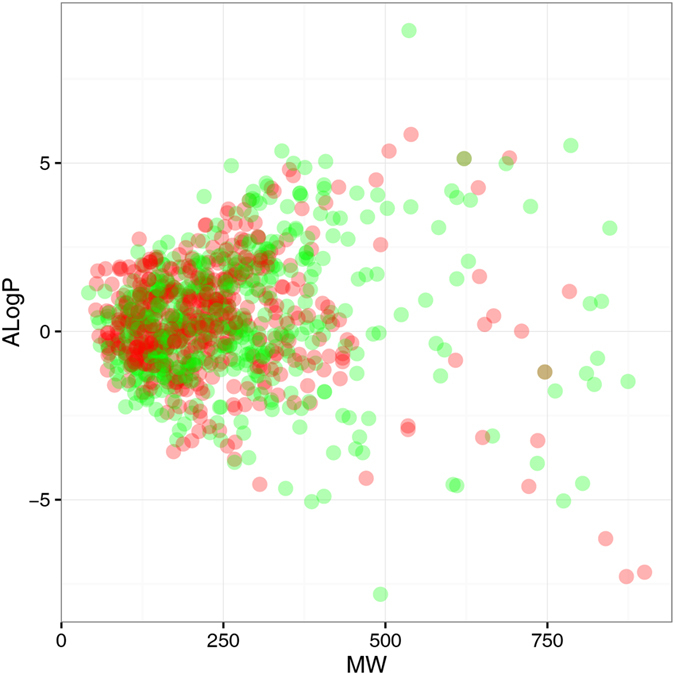
Chemical space of the training set. The chemical space is defined by the molecular weight (MW) on the X-axis and the logarithm of the octanol/water partition coefficient (ALogP) on the Y-axis. Carcinogens and non-carcinogens are represented by red and green dots, respectively.

**Figure 2:**
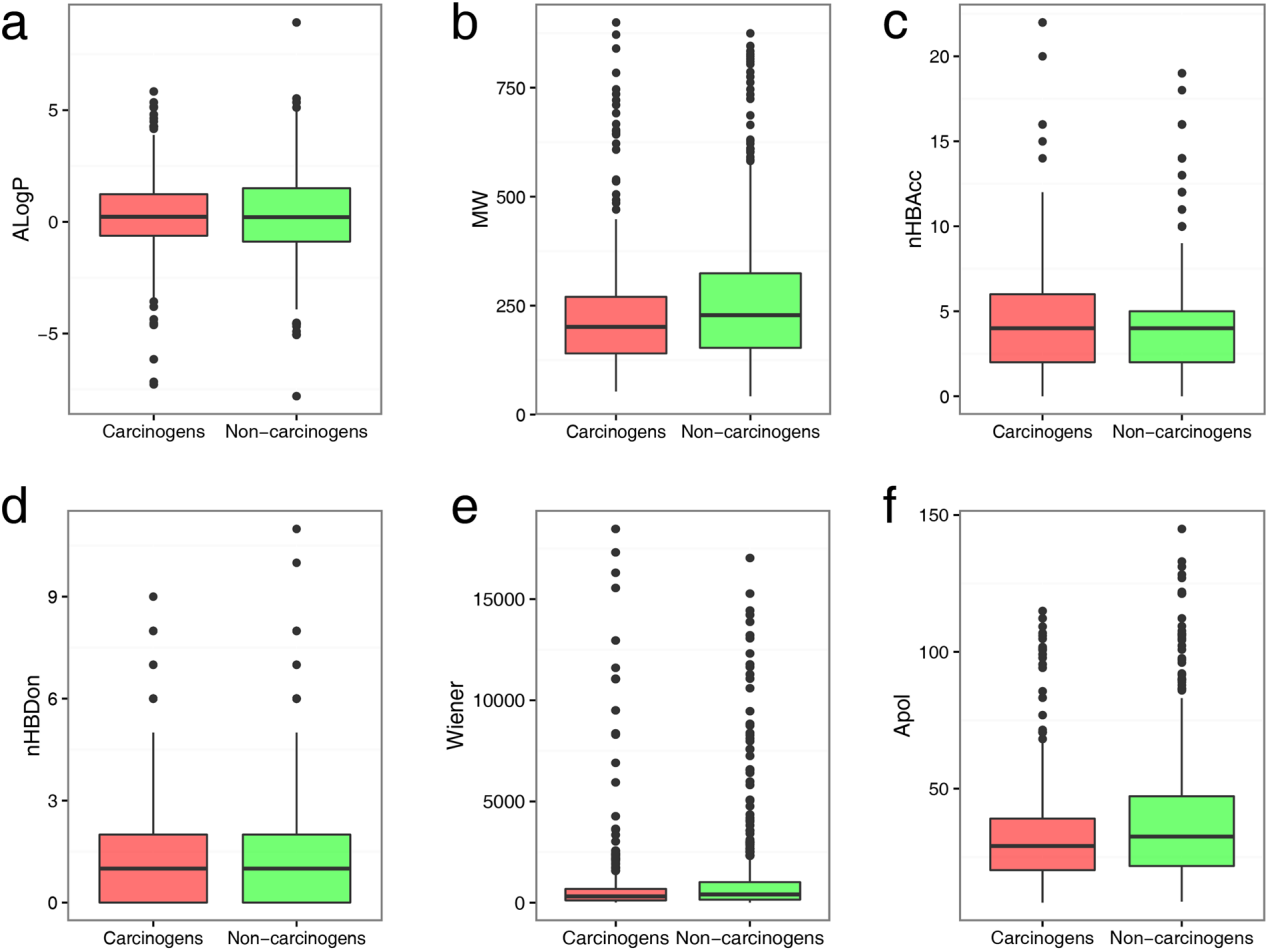
Box plot representing the molecular descriptors for carcinogens and non-carcinogens. Carcinogens and non-carcinogens are represented by red and green boxes, respectively.

In this study, 1003 compounds collected from the Carcinogenic Potency Database (CPDB) were used as training data for building and validating the predictive models. This dataset contained 494 carcinogens and 509 non-carcinogens. As we know, the diversity of compounds in a database has an important effect on the prediction accuracy of a model. The chemical space distribution of the training set can be illustrated by a scatter plot of molecular weight (MW) against the logarithm of the octanol/water partition coefficient (AlogP) for both carcinogens and non-carcinogens. As shown in Fig. 1, the MW and ALogP of carcinogenic and non-carcinogenic compounds have a similar distribution, with MW ranging from approximately 50–900 Da and AlogP ranging from approximately −7–6, which is a broader range than that of most drugs. It is also clear from Fig. 1 that we cannot distinguish carcinogenic from non-carcinogenic compounds using MW and ALogP alone, because they occupy an overlapping chemical space. In addition to MW and AlogP, the distribution of four widely used molecular descriptors was investigated (number of hydrogen bond acceptors (nHBAcc), number of hydrogen bond donors (nHBDon), Weiner path number (Weiner), and the sum of the atomic polarizabilities (Apol)). Their overall distribution is shown in Fig. 2. These box plots reveal that carcinogens tend to have slightly smaller MW and Apol than non-carcinogens (Fig. 2b and f) as deduced from the median and the first and third quartiles of the box plots. For the distribution of ALogP, nHBAcc, nHBDon, and Weiner, there is no significant difference between carcinogens and non-carcinogens. Thus, it is difficult to predict the carcinogenicity of a compound using only simple molecular descriptors.

### Performance of the models

Twelve types of molecular fingerprints (Table 1) were generated for the compounds in the training dataset. Feature selection was then performed to remove the zero variation and collinear bits in each type of fingerprint. Based on the resulting fingerprints, 36 basic classifiers were generated using Support Vector Machine (SVM), Random Forest (RF), and Extreme Gradient Boosting (XGBoost) algorithms. The performance of these models was evaluated by five-fold cross-validation with 100 repeats. The mean and standard deviation of the performance indicators from these runs are presented in Table 2. The accuracy (Q) of these basic classifiers ranges from 61.1–68.4% and the area under the curve (AUC) ranges from 65.2–74.5%. The most accurate classifier is generated by the RF algorithm using CDKExt fingerprints, whereas the highest AUC score is given by the XGBoost algorithm using CDKExt fingerprints. It can be observed that there are small differences in accuracy for the classifiers generated by different algorithms using the same fingerprints. The general rule is that RF almost always achieves slightly higher accuracy than SVM and XGBoost. A bigger difference was observed among the classifiers generated using different fingerprints. The classifiers generated using the Estate (64.2% in RF model), FP4 (62.1%), FP4C (63.6%), AP2D (64.1%), and AP2DC (64.7%) fingerprints have significantly lower accuracy than those constructed from the other fingerprints. Hence, the basic classifiers generated based on the other seven fingerprints (CDK, CDKExt, CDKGraph, MACCS, Pubchem, KR, and KRC) were fused to develop ensemble models.

**Table 1:** Performance of the basic classifiers in five-fold cross-validation. The performance values are represented as means and standard deviation.

Algorithms	Fingerprints	Q (%)	SE (%)	SP (%)	AUC (%)
SVM	CDK	67.5 ± 2.9	63.5 ± 4.9	71.5 ± 4.9	73.8 ± 3.0
	CDKExt	67.9 ± 2.9	62.9 ± 5.1	72.7 ± 4.9	73.7 ± 3.2
	CDKGraph	65.0 ± 3.1	61.5 ± 5.2	68.4 ± 5.0	69.4 ± 3.4
	Estate	63.0 ± 2.9	57.8 ± 5.3	68.0 ± 5.0	68.3 ± 3.2
	MACCS	67.1 ± 3.1	63.6 ± 5.1	70.6 ± 4.9	72.0 ± 3.3
	Pubchem	68.1 ± 3.0	64.7 ± 4.9	71.5 ± 4.5	72.8 ± 3.2
	FP4	64.6 ± 3.0	63.7 ± 4.9	65.4 ± 5.0	68.9 ± 3.1
	FP4C	62.2 ± 3.2	62.6 ± 5.0	61.8 ± 5.1	65.5 ± 3.5
	KR	66.5 ± 2.9	65.7 ± 4.9	67.2 ± 4.8	71.9 ± 3.1
	KRC	66.7 ± 3.0	67.5 ± 4.9	66.0 ± 5.1	72.1 ± 3.2
	AP2D	63.5 ± 3.0	56.3 ± 5.3	70.5 ± 5.4	68.3 ± 3.4
	AP2DC	63.4 ± 3.0	57.0 ± 6.1	69.7 ± 5.9	68.9 ± 3.2
RF	CDK	68.3 ± 3.0	64.5 ± 5.1	72.1 ± 4.5	74.1 ± 3.1
	CDKExt	68.4 ± 2.9	63.9 ± 4.8	72.8 ± 4.4	74.3 ± 3.1
	CDKGraph	66.6 ± 2.8	64.0 ± 4.7	69.0 ± 4.4	71.3 ± 3.1
	Estate	64.2 ± 3.0	61.6 ± 4.8	66.7 ± 4.9	69.9 ± 3.2
	MACCS	67.4 ± 2.9	63.4 ± 4.6	71.3 ± 4.4	73.1 ± 2.9
	Pubchem	68.0 ± 3.0	65.7 ± 4.9	70.3 ± 4.6	74.2 ± 3.1
	FP4	62.1 ± 3.0	65.3 ± 4.8	59.1 ± 5.0	66.8 ± 3.4
	FP4C	63.6 ± 3.2	63.9 ± 5.0	63.3 ± 4.9	67.9 ± 3.5
	KR	67.0 ± 2.9	66.5 ± 4.8	67.6 ± 4.9	73.3 ± 3.0
	KRC	66.5 ± 2.9	68.0 ± 4.5	65.1 ± 4.6	73.0 ± 3.0
	AP2D	64.1 ± 2.9	56.5 ± 5.1	71.5 ± 4.7	68.2 ± 3.2
	AP2DC	64.7 ± 3.0	59.6 ± 5.4	69.7 ± 4.9	70.9 ± 3.3
XGBoost	CDK	67.0 ± 3.0	65.9 ± 5.1	68.2 ± 4.9	73.6 ± 3.0
	CDKExt	68.3 ± 2.9	66.0 ± 4.5	70.6 ± 4.4	74.5 ± 2.9
	CDKGraph	65.1 ± 3.1	64.7 ± 4.6	65.5 ± 4.8	70.8 ± 3.2
	Estate	63.0 ± 2.9	60.9 ± 4.8	65.0 ± 4.8	69.5 ± 3.0
	MACCS	67.2 ± 2.9	65.5 ± 4.9	68.8 ± 4.7	73.2 ± 2.9
	Pubchem	67.8 ± 3.1	66.7 ± 5.2	68.8 ± 4.8	73.8 ± 3.2
	FP4	62.5 ± 2.7	66.1 ± 4.6	59.0 ± 4.4	65.9 ± 3.1
	FP4C	61.1 ± 3.2	61.3 ± 4.9	60.8 ± 5.1	65.2 ± 3.3
	KR	66.0 ± 3.0	66.8 ± 4.8	65.2 ± 4.9	72.7 ± 3.0
	KRC	66.5 ± 3.1	66.2 ± 4.8	66.8 ± 4.7	73.0 ± 3.1
	AP2D	64.4 ± 3.0	59.0 ± 5.0	69.5 ± 4.7	70.0 ± 3.3
	AP2DC	64.4 ± 3.2	60.9 ± 5.2	67.7 ± 4.8	70.9 ± 3.3

**Table 2:** Performance of ensemble models in five-fold cross-validation. The performance values are represented as means and standard deviation.

Models	Fingerprints	Q (%)	SE (%)	SP (%)	AUC (%)
Ensemble SVM	Top 7	69.4 ± 2.9	65.2 ± 5.2	73.5 ± 4.6	75.6 ± 3.0
Ensemble RF	Top 7	69.2 ± 2.9	67.0 ± 5.1	71.3 ± 4.6	75.7 ± 2.9
Ensemble XGBoost	Top 7	70.1 ± 2.9	67.0 ± 5.0	73.1 ± 4.4	76.5 ± 2.9
Ensemble SVM 2	All 12	69.1 ± 3.0	64.3 ± 5.3	73.7 ± 4.7	76.0 ± 3.1
Ensemble RF 2	All 12	68.6 ± 2.9	65.5 ± 4.9	71.6 ± 4.6	75.5 ± 3.0
Ensemble XGBoost 2	All 12	69.8 ± 3.0	65.8 ± 5.0	73.7 ± 4.5	76.6 ± 3.0

The performance of the ensemble models generated using these seven fingerprint sets were evaluated by five-fold cross-validation with 100 repeats. The results are shown in Table 2. It is interesting that the three ensemble models achieve significantly higher accuracy and AUC than any basic classifier. The accuracy of SVM, RF, and XGBoost improves by 1.3%, 0.8%, and 1.8%, respectively, and the AUC improves by 1.8%, 1.4%, and 2.0%, respectively, compared with the best basic classifier built by the same algorithm. Although the RF algorithm has the highest accuracy of the basic classifiers, the ensemble method exhibits the least improvement, resulting in performance equivalent to the ensemble SVM. Ensemble XGBoost is the most improved model, with performance indicators of accuracy, sensitivity, specificity, and AUC of 70.1 ± 2.9%, 67.0 ± 5.0%, 73.1 ± 4.4%, and 76.5 ± 2.9%, respectively. Ensemble models were also trained and evaluated using all 12 fingerprints. The resulting accuracy is lower than when using only the top-seven fingerprint sets, but still higher than the basic classifiers. Clearly, the ensemble method significantly improves the performance of SVM, RF and XGBoost in predicting the carcinogenicity of chemicals.

Furthermore, an external validation dataset containing 40 compounds from the ISSCAN database was used to further evaluate the performance of the ensemble models built using the top-seven fingerprint sets. Because these compounds were not involved in the construction of the models, the resulting performance reflects the ability of the models to predict the carcinogenicity of new compounds. The results in Table 3 indicate that all three models produce high overall prediction accuracy, comparable to that in five-fold cross-validation. Ensemble XGBoost is still the most accurate model. In addition, the AUC of the three models is very high, suggesting that the ensemble models have a good ability to sort the carcinogenic potential of the compounds. These results indicate that the three models established using seven types of molecular fingerprints and the ensemble learning methods can discriminate carcinogenic and non-carcinogenic compounds in both training data and external validation data with high accuracy.

**Table 3:** Performance of ensemble models and some existing software in the external validation dataset.

Models	Type	Q (%)	SE (%)	SP (%)	AUC (%)
Ensemble SVM	machine learning	67.5	60.9	76.5	81.8
Ensemble RF	machine learning	65.0	56.5	76.5	80.1
Ensemble XGBoost	machine learning	70.0	65.2	76.5	80.3
admetSAR	machine learning	50.0	34.8	70.6	49.6
PreADMET	machine learning	62.5	52.2	76.5	—^a^
VEGA CAESAR	machine learning	70.0	65.2	76.5	—^a^
VEGA ISS	rule based	70.0	73.9	64.7	—^a^
VEGA IRFMN/Antares	rule based	70.0	78.3	58.8	—^a^
VEGA IRFMN/ISSCAN-CGX	rule based	75.0	82.6	64.7	—^a^
Toxtree	rule based	70.0	78.3	58.6	—^a^
lazar	similarity search	75.0	87.0	58.8	—^a^

^a^The AUC cannot be calculated for this software because there are no probability values in its results.

These results indicate that the ensemble models will be useful for predicting the carcinogenicity of chemicals. For convenient use of these three ensemble models, a user-friendly web server called **CarcinoPred-EL** has been established (http://ccsipb.lnu.edu.cn/toxicity/CarcinoPred-EL/).

In addition, the method proposed in this study for building the ensemble model using different types of molecular fingerprints can be conveniently applied to the prediction of other toxicity endpoints or other pathological drug properties of chemicals. Since the molecular fingerprints generating methods and the machine learning algorithms used in this study are all publicly available, these models can be implemented as a software module in other programming environments, and integrated into larger software for predicting carcinogenicity and other pathological drug properties.

### Comparison with previous methods

Previously, a number of computational methods have been established for predicting the carcinogenicity of chemicals. Here, we only compare with the SAR methods that have been evaluated using proper cross-validation, because the prediction performance estimated from conventional validation (dividing the dataset into two parts, one for training and one for testing) may be biased by the single split of data. The performance indicators and evaluation method of some recent reported chemical carcinogenicity classification models are summarized in Table 4. From Tables 2 and 4, we can see the following. (1) Compared with previous models, the three proposed ensemble models achieve high overall accuracy (Q). In detail, the IRFMN/ISSCAN-CGX^29^ model implemented in VEGA software^42^ achieves an overall accuracy of 72%, which is higher than that of our models. The MDL-QSAR^14^ model achieves an overall accuracy comparable to our models. And the overall accuracy of other models was lower. (2) Although the specificity (SP) of the ensemble models is lower than that of MDL-QSAR, lazar, and Naïve Bayesian, their sensitivity (SE) is significantly higher (about 5%). SE represents the ability to correctly identify carcinogens. This is considered to be a more important indicator of the quality of a predictor for carcinogenicity classification^8^ because, for human health safety, successful prediction of carcinogens is more important than successful prediction of non-carcinogens. It is noteworthy that the sensitivity of the two rule-based models (IRFMN/Antares and IRFMN/ISSCAN-CGX in VEGA) is significantly higher. But their specificity is low. (3) All models, excepting the two rule-based models, yield larger SP and smaller SE. This phenomenon suggests that lower specificity may be a common characteristic of machine learning based carcinogenicity prediction models. To overcome this drawback, future research should consider not only the overall accuracy of a model, but also its sensitivity. Note that the differences between SP and SE of the three ensemble models are 8.3% (Ensemble SVM), 4.3% (Ensemble RF), and 6.1% (Ensemble XGBoost), which is much less than the 9.5% (MC4PC), 12.0% (MDL-QSAR), 13.5% (lazar), and 22% (Naïve Bayesian) of the other machine learning based models. This indicates that our approach has made some advances in improving the sensitivity of machine learning based models.

**Table 4:** Performance indicators and the evaluation method of some carcinogenicity classification models reported in the literature.

Model name	Evaluation method	Q (%)	SE (%)	SP (%)	Reference
MC4PC^a^	10-fold CV^e^	66.5	61.4	70.9	14
MDL-QSAR^b^	10-fold CV	69.2	62.8	74.8	14
lazar	LOOCV^f^	66.9	59.9	73.4	15
Naïve Bayesian	5-fold CV	68	57	79	16
CP ANN MDL^c^	5-fold CV^g^	66	—	—	12
CP ANN Dragon (VEGA CAESAR)^c^	5-fold CV	62	—	—	12
VEGA IRFMN/Antares	5-fold CV	66.0	83.1	48.3	29
VEGA IRFMN/ISSCAN-CGX^d^	5-fold CV	72.7	76.5	61.8	29

^a^The coverage of this model was 96%. ^b^The coverage of this model was 97%. ^c^This study did not provide the SE and SP of the models. ^d^This model was trained using carcinogenesis data from both rats and mice. ^e^Ten-fold cross-validation. ^f^Leave-one-out cross-validation. ^g^Five-fold cross-validation.

Moreover, the performance of the ensemble models was compared with some existing software, including admetSAR^43^, PreADMET^44^, VEGA^42^, Toxtree^45^, and lazar^46^, using the external validation dataset. Table 3 presents performance indicators for the three ensemble models, the T_Carc_I model^13^ in admetSAR, the Carcino_Rat model in PreADMET, the four VEGA models (CAESAR^12^, ISS^27, 28^, IRFMN/Antares^29^, and IRFMN/ISSCAN-CGX^29^), the ISS model^27, 28^ implemented in Toxtree, and the rat carcinogenicity endpoint^15^ of lazar. As shown in the table, the accuracy of admetSAR and PreADMET on this dataset is 50.5% and 62.5%, respectively, significantly lower than in the three ensemble models. The low accuracy of these software models is mainly due to their low sensitivity, indicating a poor ability to identify carcinogens. The CAESAR model in the VEGA software offers similar predictive performance to the Ensemble XGBoost model, and outperforms the Ensemble SVM and Ensemble RF models. The CAESAR model (CP ANN Dragon in Table 4) was also evaluated using five-fold cross-validation, achieving an overall accuracy of 62%^12^. The IRFMN/ISSCAN-CGX model has an accuracy of 75%, but this high score may be due to some overlap of the data source for the external validation dataset (from ISSCAN) with the training set of the IRFMN/ISSCAN-CGX model (from ISSCAN and CGX). The rule-based models (i.e., ISS, IRFMN/Antares, and IRFMN/ISSCAN-CGX) achieve high accuracy of approximately 70.0% and significantly higher sensitivity than machine learning-based methods, indicating that they have a very strong ability to identify carcinogens. However, their specificity is significantly lower than that of machine learning methods. This indicates that machine learning algorithms tend to accurately predict non-carcinogenic chemicals, whereas rule-based expert systems tend to accurately predict carcinogenic chemicals. The lazar model, using a modified kNN algorithm, has the best accuracy and sensitivity among the tested software, but its performance is relatively poor in leave-one-out cross-validation (Table 4).

As shown above, the three ensemble models have achieved good performance. Nevertheless, there are still some weakest links. Obviously, the execution speed of the ensemble models is slow. When predicting carcinogenicity of new compounds, seven types of molecular fingerprints will firstly be generated. Based on these fingerprints, seven different basic classifiers will be performed, and their results will be averaged to generate the final prediction. This process is relatively computationally expensive, causing the ensemble model to be the slowest among the above mentioned software. Secondly, as with other machine learning based models, the sensitivity of the ensemble models is relatively low. In the future studies, increasing sensitivity should be prioritized. Moreover, as these models predict carcinogenicity of new compounds based on the rule mined from known dataset, although the performance of these models were carefully evaluated by 100 times five fold cross-validation and an external validation, these models are still likely to produce unreliable results when predicting novel compounds that shares few substructure to the compounds in the training dataset. Therefore, these models currently are not suitable to be a standard tool for evaluating carcinogenicity of new compounds, they are preferably suitable for preliminary screening of carcinogenic compounds in early stages of drug discovery.

### The effect of sample size on the performance of the ensemble models

**Figure 3:**
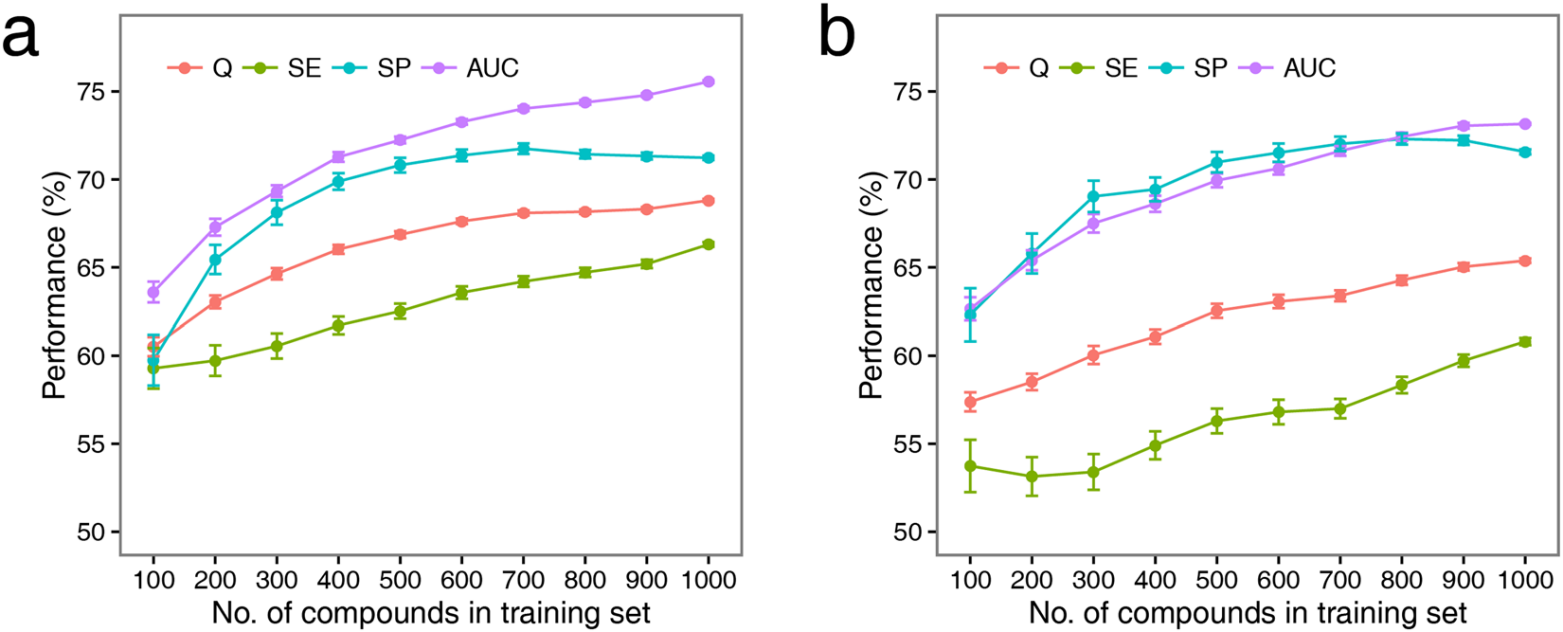
Performance on five-fold cross-validation (**a**) and external validation (**b**) as a function of number of compounds in training set for RF ensemble models. The performance values are represented as means and standard error.

To understand the effect of the number of compounds in the training set on the performance of the resulting ensemble model, the 1003 compounds in the training set were randomly sampled to form datasets containing 100–1000 (at intervals of 100) compounds, and a new ensemble RF model was trained for each dataset. Its performance was then evaluated by five-fold cross-validation and an external validation. The above sampling, training, and evaluation process was repeated 100 times to avoid any bias. As this process is computationally intensive, only the ensemble RF model (in which there is no need to tune the parameters) was investigated. As shown in Fig. 3, the results from five-fold cross-validation (Fig. 3a) and the external validation (Fig. 3b) show that the mean specificity for the RF ensemble model reaches a maximum with 700 compounds in the dataset, and then decreases slightly as the number of compounds increases to 1000. This implies that the available data may have already provided sufficient information on non-carcinogens for the RF ensemble model. It is obvious that the mean accuracy, sensitivity, and AUC increase with the number of compounds in the training set, and these metrics do not reach a plateau until the sample size reaches 1000. These results suggest that the performance, especially the accuracy, sensitivity, and AUC, of the ensemble models could be further improved by the use of more data.

### Substructures related to carcinogenicity

**Figure 4:**
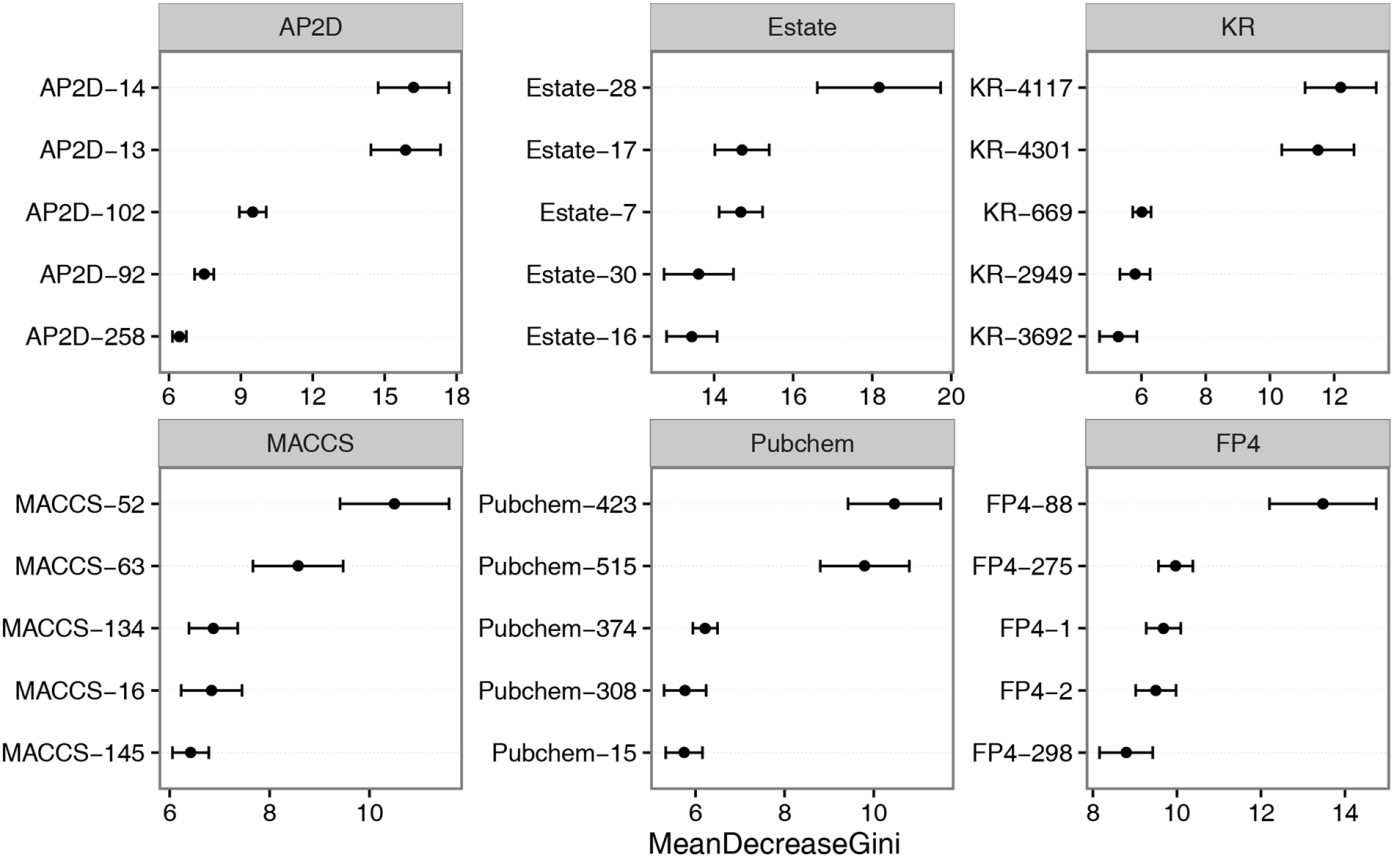
Feature importance results for top-five features from each RF model trained with Estate, MACCS, Pubchem, FP4, KR, and AP2D fingerprints. The MeanDecreaseGini values are represented as means and standard deviation.

The RF algorithm can estimate the importance of the features used in the model by calculating the mean decrease of the Gini index (MeanDecreaseGini) for each feature. Molecular substructures related to the carcinogenicity of the compounds can be identified by analysing the important bits in the molecular fingerprints. In the present study, feature importance analysis was performed for the RF models trained with the Estate, MACCS, Pubchem, FP4, KR, and AP2D fingerprints. The five most important features (with larger values of MeanDecreaseGini) for each fingerprint are shown in Fig. 4. As shown in the figure, there are 10 features with significantly higher MeanDecreaseGini values, suggesting that the substructures represented by these features may be closely related to the carcinogenicity of chemicals. The description and number of occurrences in carcinogens and non-carcinogens for these substructures are listed in Table 5. It can be seen that most of the top-ranking substructures are nitrogen-containing groups, such as the N-N, N-O, N = O, -N = groups, which occur more often in carcinogens than in non-carcinogens. Many of the known carcinogenic compounds, such as nitrosamines and nitrosoureas, contain these substructures. These features are components in many structure alerts (SA) that are used to build rule-based carcinogenicity classifiers, e.g., Aliphatic N-nitro, Alkyl nitrite, and Nitro aromatic SAs developed by Benigni *et al*.^27^. The fingerprint key of FP4-88 shows that carboxylic acid derivative is mostly present in non-carcinogens. Although the patterns of the substructures proposed in this study are very simple, and may not be suitable as SAs for rule-based carcinogenicity prediction, these substructures should be taken into consideration in the early stages of drug design.

**Table 5:** Top ranking substructures and their corresponding description and the number of occurrence in carcinogens and non-carcinogens.

Fingerprint Key	Description	SMARTS Pattern	Present in Carcinogens	Present in Non-Carcinogens
AP2D-14	N-O at topological distance 1	[#7]~[#8]	175	67
AP2D-13	N-N at topological distance 1	[#7]~[#7]	160	54
Estate-28	dsN	[ND2H0]( = *)-*	155	69
KR-4117	N = O	N = O	162	59
KR-4301	NN	NN	137	40
MACCS-52	NN	[#7]~[#7]	160	54
MACCS-63	N = O	[#7] = [#8]	162	59
Pubchem-423	N = O	[#7] = ,:[#8]	163	60
Pubchem-515	N-N-C-C	N-N-C-C	131	43
FP4-88	Carboxylic acid derivative	[$([#6X3 H0][#6]),$([#6X3H])]( = [!#6])[!#6]	136	234

### Case studies: discovery of potential carcinogens in drugs

Carcinogenicity is a serious adverse drug reaction that has already occurred in many approved drugs. As reviewed by Onakpoya *et al*.^3^, 61 medicinal products were withdrawn from the market between 1953 and 2013 because of carcinogenicity, accounting for 13% of all withdrawn. Some approved drugs or experimental drugs may also be carcinogens^47^. To identify potential carcinogens in drugs, the three ensemble models proposed in this study were employed to predict the carcinogenicity of 6538 approved and experimental small molecular drugs from the DrugBank database^48^. Among these drug molecules, 634 were predicted to be carcinogens by the ensemble SVM model, 554 were predicted to be carcinogens by the ensemble RF model, 742 were predicted to be carcinogens by the ensemble XGBoost model, and 394 were simultaneously predicted to be carcinogens by all three ensemble models. Among these 394 compounds, 61 were duplicates of compounds in the training set. Thus, our models have identified 333 potentially carcinogenic drugs.

We investigated the carcinogenicity of those drugs with predicted probabilities greater than 0.8 by searching the literature. The results are presented in Table 6. Four approved drugs are classified by the International Agency for Research on Cancer (IARC). Carmustine, Trypan blue, and Lomustine are Group 2 carcinogens, denoting that they are probably carcinogenic to humans and definitely carcinogenic to experimental animals, whereas Furazolidone is a Group 3 carcinogen that is recognized as a genotoxic carcinogen by the FAO/WHO Expert Committee on Food Additives because of its increased incidence of malignant tumours in mice and rats. Fotemustine and 1-Aminoanthracene have been reported to be a base-pair mutagen to Salmonella and to have genotoxic potency in Drosophila^49–51^. Additionally, 9-hydroxy aristolochic acid is a derivative of aristolochic acid, which is a strong carcinogen and has been classified by the IARC as a Group 1 agent. Five of the drugs predicted to be carcinogens are corticosteroids with very similar chemical structures, with Flunisolide recognized as causing an increased incidence of mammary adenocarcinomas in female rats in a long-term carcinogenesis assay^52^. We did not find any studies on carcinogenicity for the three experimental drugs 1,8-Dihydroxy-4-Nitroanthraquinone, Iodoindomethacin, and tert-butyl N-[cyano(methyl)amino]carbamate. As these experimental drugs were predicted as having a high probability of being carcinogens, developers should pay close attention to their carcinogenicity.

**Table 6:** Predicted carcinogenic drugs with predicted probabilities >0.8.

DrugBank ID	Name	Probabilities			Remarks
		SVM	RF	XGBoost	
DB00262	Carmustine	0.8	0.87	0.96	IARC Group 2A
DB09158	Trypan blue	0.78	0.91	0.95	IARC Group 2B
DB00614	Furazolidone	0.73	0.85	0.94	IARC Group 3
DB01206	Lomustine	0.71	0.78	0.91	IARC Group 2A
DB04106	Fotemustine	0.71	0.74	0.89	Mutagen to Salmonella
DB01260	Desonide	0.73	0.83	0.87	Corticosteroid
DB03035	1,8-Dihydroxy-4-Nitroanthraquinone	0.72	0.73	0.85	—
DB00288	Amcinonide	0.69	0.73	0.84	Corticosteroid
DB02636	9-hydroxy aristolochic acid	0.65	0.69	0.83	Derivative of aristolochic acid (IARC Group 1)
DB07983	Iodoindomethacin	0.64	0.7	0.82	—
DB00591	Fluocinolone Acetonide	0.73	0.82	0.81	Corticosteroid
DB00180	Flunisolide	0.73	0.81	0.81	Corticosteroid
DB01047	Fluocinonide	0.69	0.73	0.81	Corticosteroid
DB08594	tert-butyl N-[cyano(methyl)amino]carbamate	0.69	0.64	0.8	—
DB01976	1-Aminoanthracene	0.71	0.8	0.73	Mutagen to Genotoxic to DrosophilaSalmonella

The full list of 333 potentially carcinogenic drugs along with their predicted probabilities is presented in Supplementary Table S1.

## Conclusions

In this study, three novel ensemble machine learning models (Ensemble RF, Ensemble SVM, and Ensemble XGBoost) were developed to predict the carcinogenicity of chemicals in rats using molecular fingerprint representations of 1003 structurally diverse compounds. The ensemble models outperformed their basic classifiers in both overall accuracy and AUC. The best ensemble model (Ensemble XGBoost) attained an average accuracy of 70.1 ± 2.9%, sensitivity of 67.0 ± 5.0%, specificity of 73.1 ± 4.4%, and AUC of 76.5 ± 2.9% in five-fold cross-validation and an accuracy of 70.0%, sensitivity of 65.2%, specificity of 76.5%, and AUC of 80.3% in external validation. Compared with recent carcinogenicity predictors, the new ensemble models yielded good prediction quality, as demonstrated by their high accuracy and sensitivity in cross-validation. Compared with some existing software using an external validation dataset, the new ensemble models yielded high accuracy and sensitivity among machine learning-based models and similar accuracy but significantly lower sensitivity than rule-based systems. By analysing the effect of sample size on the performance of the ensemble models, we found that the accuracy, sensitivity, and AUC of the ensemble models could be further improved in the future when more data are available. Moreover, some substructures related to carcinogenicity were identified from six types of molecular fingerprints. As an application of the proposed models, 333 potentially carcinogenic drugs were identified from the DrugBank database. These models could be useful in the early stages of drug discovery for filtering potential carcinogens. The ensemble methods used in this paper could also be extended to predict other toxicity end points.

The three ensemble models have been integrated into a web server, which is freely available at http://ccsipb.lnu.edu.cn/toxicity/CarcinoPred-EL/.

## Materials and Methods

### Data Preparation

The training dataset used to develop models for predicting the carcinogenicity of diverse organic compounds was derived from the Carcinogenic Potency Database (CPDB) summary tables (CPDBAS, version 5d)^53^, which is a unique and standardized resource of long-term animal carcinogenesis study results on more than 1500 chemical substances. In the CPDB, chemicals are labelled as active (carcinogens) or inactive (non-carcinogens) according to their TD50 values. In the present study, we only considered the carcinogenicity data of the compounds against rats, as the results from rats were considered more suitable for predicting human carcinogenicity^54, 55^. To build robust predictive models, the following compounds were excluded: (1) compounds containing less than three carbon atoms; (2) compounds containing heavy metals; (3) polymers; (4) mixtures. As a result, 1003 compounds for rat carcinogenicity, including 494 carcinogens and 509 non-carcinogens, were remained as the training set for building predictive models. The details of the molecules in the training dataset are provided in Supplementary Table S2.

To further evaluate the predictive performance of the models, an external validation dataset containing compounds that do not duplicate the training dataset was constructed from the ISSCAN database^56^. The external test set contained 40 compounds, of which 23 are carcinogenic compounds and 17 are non-carcinogenic compounds. Details of the molecules in the external validation dataset are provided in Supplementary Table S3.

The DrugBank database version 5.0^48^ contains 8246 approved and experimental drug entries. We selected 6538 small molecular drugs that matched the selection criteria of the training set from this database. As an application example, the carcinogenicity of these molecules was estimated by our predictive models.

### Calculation of Molecular Fingerprints

In this study, twelve types of molecular fingerprints were generated to represent the chemical structures of the compounds. The fingerprints and their corresponding size and pattern type are summarized in Table 7. All the fingerprints were generated by the PaDEL-Descriptor software (version 2.21)^57^. Prior to the generation of the molecular fingerprints, salt was removed using the *-removesalt* parameter of the PaDEL-Descriptor. Each bit of these molecular fingerprints was used as a feature in the machine learning process.

**Table 7:** Summary of the 12 types of molecular fingerprints.

Fingerprint Type	Abbreviation	Pattern Type	Size (bits)	Selected (bits)
CDK	CDK	Hash fingerprints	1024	931
CDK Extended	CDKExt	Hash fingerprints	1024	942
CDK Graph	CDKGraph	Hash fingerprints	1024	233
Estate	Estate	Structural features	79	19
MACCS	MACCS	Structural features	166	84
Pubchem	Pubchem	Structural features	881	106
Substructure	FP4	Structural features	307	31
Substructure Count	FP4C	Structural features count	307	27
Klekota-Roth	KR	Structural features	4860	97
Klekota-Roth Count	KRC	Structural features count	4860	59
2D Atom Pairs	AP2D	Structural features	780	47
2D Atom Pairs Count	AP2DC	Structural features count	780	25

To investigate the chemical space distribution of the compounds, six molecular descriptors (logarithm of the octanol/water partition coefficient (ALogP), molecular weight (MW), number of hydrogen bond acceptors (nHBAcc), number of hydrogen bond donors (nHBDon), Weiner path number (Weiner), and sum of the atomic polarizabilities (Apol)) that are widely used in ADME/T prediction were also calculated by the PaDEL-Descriptor.

### Feature selection

Feature selection is an important procedure for building predictive models. The deletion of redundant features can simplify the generated model, prevent overfitting, and enhance the generalization ability of the model. In this study, features that had only a single unique value (zero variation features) in the training dataset were identified and removed using the *nearZeroVar* function from the R package *caret* (version 6.0–71)^58^. The pairwise Pearson’s correlation coefficients among the remaining features were then calculated. Highly correlated features (Pearson’s correlation coefficients >0.7) were filtered out using the *findCorrelation* function from *caret* ^58^. The remaining features (bits) for each molecular fingerprint are summarized in Table 7.

### Model building

Ensemble machine learning models formed by fusing a series of simple independent models (base classifiers) via voting or averaging usually produce more accurate results than any of the single models.

**Figure 5:**
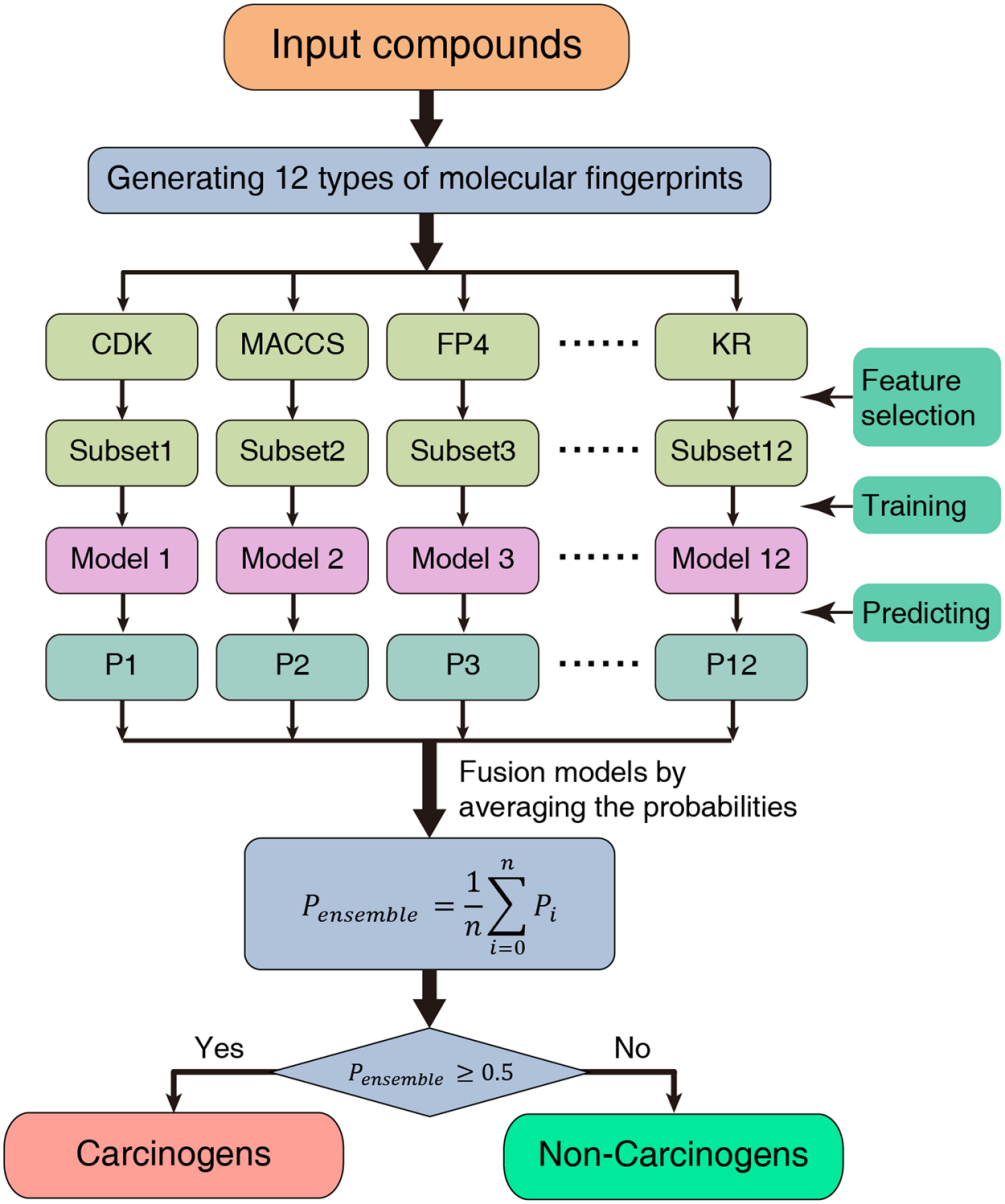
Flowchart to show the ensemble model building process.

In this study, three ensemble models were proposed using three different machine learning algorithms, namely SVM, RF, and XGBoost. Twelve base classifiers were built for each ensemble model by applying the different molecular fingerprints to the corresponding machine learning algorithms. The basic classifiers with better predictive performance were fused to form the ensemble model via averaging the probabilities from the basic classifiers. A flowchart of the ensemble model building process is shown in Fig. 5.

The SVM, RF, and XGBoost algorithms were all executed in R (version 3.3.1) using the *kernlab* (version 0.9–25)^59^, *randomForest* (version 4.6–12)^60^, and *xgboost* (version 0.4–4)^61^ packages, respectively. A brief description of the basic theory of each algorithm and how they were used in this study is provided below.

#### Support vector machine

An SVM is an efficient supervised machine learning method based on the principle of structural risk minimization. This algorithm maps the input data into a high-dimensional feature space through some kernel functions and constructs an optimal separating hyperplane in this space. In this study, the radial basis kernel function (RBF) was used to implement the SVM models. The regularization parameter *C* and the kernel width parameter *gamma* were optimized through the random search method^62^, which was implemented in the *caret* package.

#### Random forest

RF is an ensemble machine learning method in which a multitude of decision trees are combined using randomly selected subsets of training samples and features. RF is considered to be more accurate and robust than decision trees. One of the most important advantages of RF is that it can handle a large number of features without overfitting, and can give an estimate of the importance of the features. There are two main parameters in RF, the number of trees in the forest (*ntree*) and the number of features randomly sampled (*mtry*). In this study, the default values of these parameters were used, that is, *ntree* = 500 and *mtry* = the square root of the number of features in the dataset. The feature importance for each type of molecular fingerprint was analysed using the *importance* function in the *randomForest* package.

#### Extreme gradient boosting

XGBoost is a new implementation of the gradient tree boosting technique. XGBoost has been tested in a series of datasets for QSAR modelling, achieving high accuracy and requiring much less computation time than deep neural nets^63^. There are several adjustable parameters in XGBoost. In this study, the step size shrinkage (*eta*), maximum depth of tree (*max.depth*), minimum sum of instance weight (min.child.weight), and the maximum number of iterations (*nrounds*) were optimized by the *caret* package.

### Performance Evaluation

The performance of the basic classifiers and ensemble models was evaluated by five-fold cross-validation with 100 repeats. In detail, the training set was randomly divided into five equal parts. Four parts were used to train the classifier, and the fifth part was used as a test set to evaluate the performance of the classifier. Thus, five classifiers and performance indicators can be obtained. To reduce the randomness of the results, the five-fold cross-validation was repeated 100 times, resulting in a total of 500 sets of performance indicators. The performance indicators were aggregated to give an accurate performance evaluation of each model. In addition, the final ensemble models and some existing methods (software) were also evaluated using an external validation dataset.

Four performance indicators were used to evaluate the models, namely accuracy (Q), specificity (SP), sensitivity (SE), and the area under the receiver operating characteristic curve (AUC). The accuracy represents the overall prediction accuracy of carcinogens and non-carcinogens. Specificity represents the predictive accuracy for non-carcinogens, and sensitivity describes the predictive accuracy for carcinogens. The indicators were calculated as follows:

1 $$Q=\frac{TP+TN}{TP+TN+FN+FP}\times 100 \% ,$$

2 $$SE=\frac{TP}{TP+FN}\times 100 \% ,$$

3 $$SP=\frac{TN}{TN+FP}\times 100 \% ,$$

where TP (true positive) denotes the number of correctly predicted carcinogens, TN (true negative) represents the number of correctly predicted non-carcinogens, FP (false positive) represents the number of non-carcinogens predicted to be carcinogens, and FN (false negative) represents the number of carcinogen compounds predicted to be non-carcinogens.

The receiver operating characteristic curve (ROC) is a plot of the TP rate (sensitivity) against the FP rate (1-specificity) for the different possible cutoff points of a diagnostic test. The AUC was calculated as an indicator of model predictiveness.

## Electronic supplementary material

Supplementary Information
