# Supplementary material for: CarcinoPred-EL: Novel models for predicting the carcinogenicity of chemicals using molecular fingerprints and ensemble learning methods
Source: Sci Rep. 2017 May 18;7:2118. doi: 10.1038/s41598-017-02365-0 (PMC5437031; doi:10.1038/s41598-017-02365-0)
Supplement: Supplementary file 1 — Supplementary Information [file 41598_2017_2365_MOESM1_ESM.pdf]

CarcinoPred-EL: Novel models for predicting the carcinogenicity of chemicals using molecular fingerprints and ensemble learning methods

Li Zhang[1,2, ¶], Haixin Ai[1,2,3, ¶], Wen Chen[4], Zimo Yin[4], Huan Hu[1], Junfeng Zhu[1], Jian Zhao[1], Qi Zhao[2,5], Hongsheng Liu[1,2,3,*]

[1] School of Life Science, Liaoning University, Shenyang, 110036, China
[2] Research Center for Computer Simulating and Information Processing of Bio-macromolecules of Liaoning Province, Shenyang, 110036, China
[3] Engineering Laboratory for Molecular Simulation and Designing of Drug Molecules of Liaoning, Shenyang, 110036, China
[4] School of Information, Liaoning University, Shenyang, 110036, China
[5] School of Mathematics, Liaoning University, Shenyang, 110036, China

[*] Corresponding author: Hongsheng Liu, E-mail: liuhongsheng@lnu.edu.cn Tel: +86-24-62202280
Fax: +86-24-62202280
[¶] These authors contributed equally to this work.

Supplementary Table S1: List of 330 potentially carcinogenic drugs along with their predicted probabilities

DrugBank ID	CAS Number	Name	Probabilities		
			RF	SVM	XGBoost
DB00262	154-93-8	Carmustine	0.87	0.8	0.96
DB09158	72-57-1	Trypan blue	0.91	0.78	0.95
DB00614	67-45-8	Furazolidone	0.85	0.73	0.94
DB01206	13010-47-4	Lomustine	0.78	0.71	0.91
DB04106	-	Fotemustine	0.74	0.71	0.89
DB01260	638-94-8	Desonide	0.83	0.73	0.87
DB03035	-	1,8-Di-Hydroxy-4-Nitro-Anthraquinone	0.73	0.72	0.85
DB00288	51022-69-6	Amcinonide	0.73	0.69	0.84
DB02636	-	9-Hydroxy-8-Methoxy-6-Nitro-Phenanthrol [3,4-D][1,3]Dioxole-5-Carboxylic Acid	0.69	0.65	0.83
DB07983	-	1-(4-IODOBENZOYL)-5-METHOXY-2-M ETHYL INDOLE-3-ACETIC ACID	0.7	0.64	0.82
DB00591	67-73-2	Fluocinolone Acetonide	0.82	0.73	0.81
DB01047	356-12-7	Fluocinonide	0.73	0.69	0.81
DB00180	3385-03-3	Flunisolide	0.81	0.73	0.81
DB08594	-	TERT-BUTYL 2-CYANO-2-METHYLHYDRAZINECAR BOXYLATE	0.64	0.69	0.8
DB04818	54-92-2	Iproniazid	0.67	0.67	0.79
DB08318	-	6-(2-phenoxyethoxy)-1,3,5-triazine-2,4-dia mine	0.64	0.68	0.79
DB09251	3818-37-9	Phenoxypropazine	0.58	0.72	0.78
DB04110	-	2-Nitro-P-Cresol	0.71	0.59	0.77
DB01950	-	N-(4-Methoxybenzyl)-N'-(5-Nitro-1,3-Thia zol-2-Yl)Urea	0.6	0.63	0.77
DB02833	-	[4-(2-Amino-4-Methyl-Thiazol-5-Yl)-Pyri midin-2-Yl]-(3-Nitro-Phenyl)-Amine	0.62	0.6	0.76
DB04417	100-02-7	P-Nitrophenol	0.73	0.64	0.76
DB09252	306-19-4	Pivhydrazine	0.6	0.67	0.75
DB08178	-	4-(4-methoxy-1H-pyrrolo[2,3-b]pyridin-3-y l)pyrimidin-2-amine	0.63	0.63	0.75
DB08182	-	4-(4-propoxy-1H-pyrrolo[2,3-b]pyridin-3-y l)pyrimidin-2-amine	0.58	0.59	0.75
DB04542	-	3'-Azido-3'-Deoxythymidine-5'-Diphosphat e	0.58	0.56	0.75
DB08660	-	1,2,5,8-tetrahydroxyanthracene-9,10-dione	0.71	0.64	0.75
DB08203	-	7-[2-METHOXY-1-(METHOXYMETHYL	0.62	0.66	0.74

		)ETHYL]-7H-PYRROLO[3,2-F] QUINAZOLINE-1,3-DIAMINE			
DB03407	3316-09-4	4-Nitrocatechol	0.66	0.6	0.74
DB04782	-	(S)-4-Nitrostyrene oxide	0.62	0.57	0.74
DB04777	-	(R)-4-Nitrostyrene oxide	0.62	0.57	0.74
DB07551	-	2-CHLORO-4-ETHYLAMINO-6-(S(-)-2'-CYANO-4-BUTYLAMINO)-1,3,5-TRIAZINE	0.75	0.7	0.73
DB07552	-	2-CHLORO-4-ETHYLAMINO-6-(R(+)-2'-CYANO-4-BUTYLAMINO)-1,3,5-TRIAZINE	0.75	0.7	0.73
DB08204	-	3-DIPHENOL-6-NITRO-3H-BENZO[DE]ISOCHROMEN-1-ONE	0.61	0.55	0.73
DB00406	14426-25-6	Gentian Violet	0.71	0.68	0.73
DB03666	-	3'-Azido-3'-Deoxythymidine-5'-Monophosphate	0.6	0.58	0.73
DB01976	62813-37-0	Aminoanthracene	0.8	0.71	0.73
DB01613	7297-25-8	Erythrityl Tetranitrate	0.62	0.53	0.73
DB09253	33419-68-0	Safrazine	0.56	0.66	0.72
DB00658	52757-95-6	Sevelamer	0.81	0.67	0.72
DB03369	-	9-Aminophenanthrene	0.79	0.72	0.72
DB03474	-	Reactive Red 1 Dye	0.62	0.53	0.72
DB09246	7654-03-7	Benmoxin	0.61	0.61	0.72
DB01129	117976-89-3	Rabeprazole	0.59	0.56	0.72
DB03840	-	Tetra(Imidazole)Diaquacopper (Ii)	0.77	0.66	0.72
DB00380	24584-09-6	Dexrazoxane	0.72	0.61	0.71
DB08519	-	N~4~-(3-methyl-1H-indazol-6-yl)-N~2~-(3,4,5-trimethoxyphenyl)pyrimidine-2,4-diamine	0.57	0.63	0.71
DB01275	86-54-4	Hydralazine	0.61	0.71	0.71
DB00448	103577-45-3	Lansoprazole	0.6	0.56	0.71
DB07248	-	7-PYRIDIN-2-YL-N-(3,4,5-TRIMETHOXYPHENYL)-7H-PYRROLO[2,3-D]PYRIMIDIN-2-AMINE	0.57	0.6	0.71
DB00740	1744-22-5	Riluzole	0.62	0.65	0.71
DB00488	645-05-6	Altretamine	0.68	0.69	0.7
DB07300	-	2-(1H-imidazol-1-yl)-9-methoxy-8-(2-methoxyethoxy)benzo[c][2,7]naphthyridin-4-amine	0.57	0.62	0.7
DB04528	51-28-5	2,4-Dinitrophenol	0.64	0.56	0.7
DB00213	102625-70-7	Pantoprazole	0.65	0.58	0.7

DB07029	-	4-(1,3-BENZODIOXOL-5-YLOXY)-2-[4-(1H-IMIDAZOL-1-YL)PHENOXY]-6-METHYLPYRIMIDINE	0.57	0.57	0.7
DB07862	-	7-(1-ETHYL-PROPYL)-7H-PYRROLO-[3,2-F]QUINAZOLINE-1,3-DIAMINE	0.62	0.66	0.7
DB08583	-	2-amino-5-[3-(1-ethyl-1H-pyrazol-5-yl)-1H-pyrrolo[2,3-b]pyridin-5-yl]-N,N-dimethylbenzamide	0.53	0.56	0.7
DB07408	-	5-(2-NITROPHENYL)-2-FUROIC ACID	0.51	0.51	0.7
DB03924	-	5,8-Di-Amino-1,4-Dihydroxy-Anthraquinone	0.68	0.69	0.7
DB08973	3693-39-8	Fluclorolone acetonide	0.68	0.65	0.7
DB06898	-	4-(2-amino-1-methyl-1H-imidazo[4,5-b]pyridin-6-yl)phenol	0.61	0.63	0.7
DB01213	7554-65-6	Fomepizole	0.62	0.59	0.69
DB07824	-	4-ethyl-5-methyl-2-(1H-tetrazol-5-yl)-1,2-dihydro-3H-pyrazol-3-one	0.59	0.61	0.69
DB00507	55981-09-4	Nitazoxanide	0.55	0.6	0.69
DB04407	-	4-[4-(4-Methyl-2-Methylamino-Thiazol-5-Yl)-Pyrimidin-2-Ylamino]-Phenol	0.52	0.54	0.69
DB07562	-	N-[4-(2,4-DIMETHYL-THIAZOL-5-YL)-PYRIMIDIN-2-YL]-N',N'-DIMETHYL-BENZENE-1,4-DIAMINE	0.6	0.56	0.69
DB00724	99011-02-6	Imiquimod	0.59	0.6	0.69
DB08983	31637-97-5	Etofibrate	0.56	0.54	0.69
DB07990	--	(RP,SP)-O-(2R)-(1-PHENOXYBUT-2-YL)-METHYLPHOSPHONIC ACID CHLORIDE	0.54	0.57	0.69
DB02105	-	3,5-Dinitrocatechol	0.63	0.56	0.69
DB08419	-	(1S)-1-(PHENOXYMETHYL)PROPYL METHYLPHOSPHONOCHLORIDOATE	0.54	0.57	0.69
DB02671	616-47-7	1-Methylimidazole	0.72	0.66	0.69
DB00911	19387-91-8	Tinidazole	0.56	0.55	0.68
DB02768	-	Tert-Butyloxycarbonyl Group	0.71	0.58	0.68
DB03789	-	2,3-Dimethylimidazolium Ion	0.73	0.67	0.68
DB08139	-	5-chloro-7-[(1-methylethyl)amino]pyrazolo[1,5-a]pyrimidine-3-carbonitrile	0.58	0.63	0.68
DB03585	129-20-4	Oxyphenbutazone	0.63	0.61	0.68
DB06932	-	10,11-dimethoxy-4-methyldibenzo[c,f]-2,7-naphthyridine-3,6-diamine	0.61	0.65	0.68
DB02402	-	5-(4-Methoxyphenoxy)-2,4-Quinazolinediamine	0.58	0.66	0.68

DB08387	-	2-{[4-(1-methyl-4-pyridin-4-yl-1H-pyrazol-3-yl)phenoxy]methyl}quinoline	0.56	0.6	0.68
DB04479	-	4-Nitro-Inden-1-One	0.64	0.62	0.68
DB09118	49763-96-4	Stiripentol	0.56	0.66	0.68
DB07008	-	4-(1,3-BENZODIOXOL-5-YLOXY)-2-[4-(1H-IMIDAZOL-1-YL)PHENOXY]PYRIMIDINE	0.57	0.57	0.68
DB09248	65-64-5	Mebanazine	0.56	0.59	0.68
DB02757	288-13-1	Pyrazole	0.65	0.61	0.67
DB07584	-	N-[2-(5-methyl-4H-1,2,4-triazol-3-yl)phenyl]-7H-pyrrolo[2,3-d]pyrimidin-4-amine	0.6	0.59	0.67
DB04069	-	5,6-Dihydro-Benzo[H]Cinnolin-3-Ylamine	0.61	0.6	0.67
DB08042	-	N~4~-methyl-N~4~-(3-methyl-1H-indazol-6-yl)-N~2~-(3,4,5-trimethoxyphenyl)pyrimidine-2,4-diamine	0.54	0.61	0.67
DB08811	22345-47-7	Tofisopam	0.53	0.6	0.67
DB09283	15421-84-8	Trapidil	0.55	0.62	0.67
DB00990	107868-30-4	Exemestane	0.54	0.64	0.67
DB02048	-	1,2,4-Triazole-Carboxamidine	0.61	0.59	0.67
DB08168	-	7-AMINO-4-METHYL-CHROMEN-2-ONE	0.61	0.58	0.67
DB04518	-	3-[4-(2,4-Dimethyl-Thiazol-5-Yl)-Pyrimidin-2-Ylamino]-Phenol	0.53	0.54	0.67
DB07179	-	3-((3-bromo-5-o-tolylpyrazolo[1,5-a]pyrimidin-7-ylamino)methyl)pyridine 1-oxide	0.55	0.58	0.67
DB09282	25717-80-0	Molsidomine	0.6	0.62	0.67
DB08639	-	4-[4-(2,4,6-TRIMETHYL-PHENYLAMINO)-PYRIMIDIN-2-YLAMINO]-BENZONITRILE	0.54	0.64	0.67
DB00733	6735-59-7	Pralidoxime	0.56	0.59	0.67
DB00389	22232-54-8	Carbimazole	0.52	0.52	0.66
DB08969	333-36-8	Flurothyl	0.7	0.63	0.66
DB08300	-	1-methyl-3-naphthalen-2-yl-1H-pyrazolo[3,4-d]pyrimidin-4-amine	0.65	0.63	0.66
DB03060	-	Sri-9662	0.61	0.61	0.66
DB03180	-	4,5-Dimethyl-1,2-Phenylenediamine	0.63	0.63	0.66
DB00846	1524-88-5	Flurandrenolide	0.68	0.63	0.66
DB04472	-	(R)-1-Para-Nitro-Phenyl-2-Azido-Ethanol	0.57	0.55	0.66
DB00696	113-15-5	Ergotamine	0.62	0.62	0.66
DB07610	-	NAPHTHALENE-1,2-DIOL	0.64	0.58	0.66
DB08006	-	N-anthracen-2-yl-5-methyl[1,2,4]triazolo[1,	0.59	0.65	0.65

		5-a]pyrimidin-7-amine			
DB07373	-	ANDROSTA-1,4-DIENE-3,17-DIONE	0.54	0.64	0.65
DB08788	-	3,6-DIAMINO-5-CYANO-4-(4-ETHOXYPHENYL)THIENO[2,3-B]PYRIDINE-2-CARBOXAMIDE	0.55	0.6	0.65
DB04491	-	Diisopropylphosphono Group	0.65	0.59	0.65
DB02583	175354-76-4	N6-(2,5-Dimethoxy-Benzyl)-N6-Methyl-Pyrido[2,3-D]Pyrimidine-2,4,6-Triamine	0.55	0.6	0.65
DB03608	536-71-0	Diminazene	0.6	0.62	0.65
DB00962	151319-34-5	Zaleplon	0.55	0.58	0.65
DB02797	13331-27-6	3-Nitrophenylboronic Acid	0.66	0.6	0.65
DB07161	-	5-phenyl-1H-indazol-3-amine	0.59	0.6	0.65
DB02721	3469-69-0	4-Iodopyrazole	0.63	0.59	0.65
DB08940	853-34-9	Kebuzone	0.66	0.58	0.65
DB03651	-	2,4,6-Trinitrophenol	0.58	0.54	0.65
DB00863	66357-35-5	Ranitidine	0.59	0.56	0.65
DB02589	-	Se-Ethyl-Isoselenourea	0.52	0.59	0.64
DB01676	118-96-7	Trinitrotoluene	0.61	0.55	0.64
DB00228	13838-16-9	Enflurane	0.65	0.6	0.64
DB07236	-	3-(6-HYDROXY-NAPHTHALEN-2-YL)-BENZO[D]ISOOXAZOL-6-OL	0.52	0.55	0.64
DB07722	-	3-(4-NITRO-PHENOXY)-PROPAN-1-OL	0.54	0.57	0.64
DB08186	-	(3E)-4-(1-METHYL-1H-INDOL-3-YL)BUT-3-EN-2-ONE	0.56	0.54	0.64
DB08054	-	1-(1-methylethyl)-3-quinolin-6-yl-1H-pyrazolo[3,4-d]pyrimidin-4-amine	0.56	0.61	0.64
DB02154	-	2,3-Bis-Benzo[1,3]Dioxol-5-Ylmethyl-Succinic Acid	0.5	0.53	0.64
DB08694	-	9-amino-5-(2-aminopyrimidin-4-yl)pyrido[3',2':4,5]pyrrolo[1,2-c]pyrimidin-4-ol	0.54	0.55	0.64
DB01219	7261-97-4	Dantrolene	0.71	0.64	0.64
DB02811	-	Diethylphosphono Group	0.63	0.65	0.64
DB08713	-	2,6-DIMETHYL-1-(3-[3-METHYL-5-ISOXAZOLYL]-PROPANYL)-4-[2N-METHYL-2H-TETRAZOL-5-YL]-PHENOL	0.53	0.59	0.64
DB08785	-	4-METHYL-2H-CHROMEN-2-ONE	0.65	0.55	0.64
DB04982	161832-65-1	Talampanel	0.5	0.58	0.64
DB04169	-	3,5-Diaminophthalhydrazide	0.53	0.54	0.64
DB05791	-	Perflubron emulsion	0.67	0.64	0.64
DB03987	-	2,4-Diamino-6-[N-(3',5'-Dimethoxybenzyl)-N-Methylamino]Pyrido[2,3-D]Pyrimidine	0.56	0.6	0.64

DB08340	-	N,N'-DIPHENYLPYRAZOLO[1,5-A][1,3,5]TRIAZINE-2,4-DIAMINE	0.51	0.61	0.64
DB00922	141505-33-1	Levosimendan	0.58	0.59	0.64
DB08715	-	2,6-DIMETHYL-1-(3-[3-METHYL-5-ISOXAZOLYL]-PROPANYL)-4-[2-METHYL-4-ISOXAZOLYL]-PHENOL	0.53	0.56	0.64
DB03764	-	4-Hydroperoxy-2-Methoxy-Phenol	0.7	0.63	0.64
DB00677	55-91-4	Isoflurophate	0.64	0.58	0.64
DB07731	-	4-[(E)-(3,5-DIAMINO-1H-PYRAZOL-4-YL)DIAZENYL]PHENOL	0.58	0.63	0.64
DB01749	110-71-4	1,2-Dimethoxyethane	0.68	0.6	0.64
DB02935	-	1-Methoxy-2-(2-Methoxyethoxy)Ethane	0.63	0.59	0.64
DB03399	-	Ethyl Isocyanide	0.57	0.63	0.63
DB03771	-	Allyl-{4-[3-(4-Bromo-Phenyl)-Benzofuran-6-Yloxy]-but-2-Enyl}-Methyl-Amine	0.53	0.6	0.63
DB08053	-	1-cyclobutyl-3-(3,4-dimethoxyphenyl)-1H-pyrazolo[3,4-d]pyrimidin-4-amine	0.56	0.64	0.63
DB03347	-	Triethyl Phosphate	0.63	0.61	0.63
DB03033	-	1-Methyloxy-4-Sulfone-Benzene	0.56	0.61	0.63
DB08461	-	3-[(4-AMINO-1-TERT-BUTYL-1H-PYRAZOLO[3,4-D]PYRIMIDIN-3-YL)METHYL]PHENOL	0.55	0.56	0.63
DB00780	51-71-8	Phenelzine	0.51	0.54	0.63
DB01509	4764-17-4	3,4-Methylenedioxyamphetamine	0.57	0.65	0.63
DB02427	-	2,4-Diamino-6-[N-(2',5'-Dimethoxybenzyl)-N-Methylamino]Quinazoline	0.55	0.6	0.63
DB00411	51-83-2	Carbachol	0.55	0.52	0.63
DB02919	-	2,4-Diamino-6-[N-(3',4',5'-Trimethoxybenzyl)-N-Methylamino]Pyrido[2,3-D]Pyrimidine	0.55	0.6	0.63
DB08146	-	7-(2,5-dihydropyrrol-1-yl)-6-phenyl-pyrido[6,5-d]pyrimidin-2-amine	0.54	0.62	0.63
DB02127	-	Methylphosphonic Acid Diisopropyl Ester	0.63	0.58	0.63
DB07511	-	4-(4-methyl-1,3-dioxo-1,3-dihydro-2H-isoindol-2-yl)benzonitrile	0.54	0.54	0.62
DB08658	-	ETHYL HYDROGEN DIETHYLAMIDOPHOSPHATE	0.58	0.65	0.62
DB00311	452-35-7	Ethoxzolamide	0.54	0.54	0.62
DB01039	49562-28-9	Fenofibrate	0.59	0.53	0.62
DB07324	-	3-({2-[(2-AMINO-6-METHYLPYRIMIDIN-4-YL)ETHYNYL]BENZYL}AMINO)-1,3-OXAZOL-2(3H)-ONE	0.55	0.57	0.62

DB01442	13674-05-0	MMDA	0.54	0.62	0.62
DB04910	20559-55-1	Oxibendazole	0.51	0.57	0.62
DB09243	3614-47-9	Hydracarbazine	0.55	0.57	0.62
DB07103	-	2-(4-METHYLPHENOXY)ETHYLPHOSPHINATE	0.56	0.59	0.62
DB00530	183321-74-6	Erlotinib	0.54	0.6	0.62
DB07488	-	{(2Z)-4-AMINO-2-[(4-METHOXYPHENYL)IMINO]-2,3-DIHYDRO-1,3-THIAZOL-5-YL}(4-METHOXYPHENYL)METHANONE	0.54	0.57	0.62
DB07462	-	(3,4-DIHYDROXY-2-NITROPHENYL)(PHENYL)METHANONE	0.58	0.54	0.62
DB01189	57041-67-5	Desflurane	0.67	0.6	0.62
DB00351	595-33-5	Megestrol acetate	0.57	0.52	0.61
DB02041	-	Isoluminol	0.54	0.57	0.61
DB08864	500287-72-9	Rilpivirine	0.54	0.63	0.61
DB02538	-	N-[4-(2-Methylimidazo[1,2-a]Pyridin-3-Yl)-2-Pyrimidinyl]Acetamide	0.59	0.6	0.61
DB07198	-	5-HYDROXY-2-(4-HYDROXYPHENYL)-1-BENZOFURAN-7-CARBONITRILE	0.59	0.62	0.61
DB07335	-	3-[4-AMINO-1-(1-METHYLETHYL)-1H-PYRAZOLO[3,4-D]PYRIMIDIN-3-YL]PHENOL	0.53	0.58	0.61
DB04463	-	3-(4-Amino-1-Tert-Butyl-1h-Pyrazolo[3,4-D]Pyrimidin-3-Yl)Phenol	0.57	0.58	0.61
DB03365	-	4-[3-Hydroxyanilino]-6,7-Dimethoxyquinazoline	0.56	0.61	0.61
DB01247	59-63-2	Isocarboxazid	0.56	0.59	0.61
DB00753	26675-46-7	Isoflurane	0.65	0.59	0.61
DB08714	-	2,6-DIMETHYL-1-(3-[3-METHYL-5-ISOXAZOLYL]-PROPANYL)-4-[4-METHYL-2H-TETRAZOL-2-YL]-PHENOL	0.53	0.59	0.61
DB08008	-	5-methyl-N-[4-(trifluoromethyl)phenyl][1,2,4]triazolo[1,5-a]pyrimidin-7-amine	0.54	0.61	0.61
DB08065	-	2-(1H-pyrazol-3-yl)-1H-benzimidazole	0.57	0.59	0.61
DB08035	-	1-TERT-BUTYL-3-(2,5-DIMETHYLBENZYL)-1H-PYRAZOLO[3,4-D]PYRIMIDIN-4-AMINE	0.54	0.59	0.61
DB01028	76-38-0	Methoxyflurane	0.66	0.59	0.61
DB07032	-	2-(4-HYDROXY-PHENYL)BENZOFURAN-5-OL	0.55	0.6	0.6
DB08479	-	N-(3,5-dimethoxyphenyl)imidodicarbonimi	0.54	0.51	0.6

		dic diamide			
DB02252	-	Iodophenyl	0.73	0.6	0.6
DB02967	128-53-0	N-Ethylmaleimide	0.54	0.55	0.6
DB04130	-	5-Methoxybenzimidazole	0.58	0.6	0.6
DB08335	-	4-HYDROXYBENZALDEHYDE O-(3,3-DIMETHYLBUTANOYL)OXIME	0.51	0.61	0.6
DB00323	134308-13-7	Tolcapone	0.59	0.54	0.6
DB08362	-	N-(3-(8-CYANO-4-(PHENYLAMINO)PYRAZOLO[1,5-A][1,3,5]TRIAZIN-2-YLAMINO)PHENYL)ACETAMIDE	0.55	0.59	0.6
DB08363	-	1-(9-ethyl-9H-carbazol-3-yl)-N-methylmethanamine	0.54	0.57	0.6
DB03836	-	1,3,5-Trichloro-Benzene	0.75	0.5	0.6
DB03695	-	6-(2,5-Dimethoxy-Benzyl)-5-Methyl-Pyrido[2,3-D]Pyrimidine-2,4-Diamine	0.59	0.59	0.6
DB08699	-	1-tert-butyl-3-(3-methylbenzyl)-1H-pyrazolo[3,4-d]pyrimidin-4-amine	0.57	0.59	0.6
DB08695	-	3-(4-nitrophenyl)-1H-pyrazole	0.62	0.6	0.6
DB01006	112809-51-5	Letrozole	0.57	0.6	0.6
DB06816	7187-62-4	Pyrvinium	0.54	0.51	0.59
DB08786	-	4-(2-methoxyethoxy)-6-methylpyrimidin-2-amine	0.56	0.59	0.59
DB03514	-	2-Methoxy-4-Vinyl-Phenol	0.57	0.55	0.59
DB03121	-	1-Benzyl-5-Methoxy-2-Methyl-1h-Indol-3-Yl)-Acetic Acid	0.56	0.52	0.59
DB00440	738-70-5	Trimethoprim	0.57	0.61	0.59
DB02915	-	4-(2,4-Dimethyl-Thiazol-5-Yl)-Pyrimidin-2-Yl]-(4-Trifluoromethyl-Phenyl)-Amine	0.5	0.55	0.59
DB08538	-	N-((2-aminopyrimidin-5-yl)methyl)-5-(2,6-difluorophenyl)-3-ethylpyrazolo[1,5-a]pyrimidin-7-amine	0.52	0.58	0.59
DB07525	-	3-(3-methoxybenzyl)-1H-pyrrolo[2,3-b]pyridine	0.58	0.59	0.59
DB01656	162401-32-3	Roflumilast	0.61	0.58	0.59
DB06967	-	6-ETHYL-5-[9-(3-METHOXYPROPYL)-9H-CARBAZOL-2-YL]PYRIMIDINE-2,4-DIAMINE	0.53	0.52	0.59
DB01612	8017-89-8	Amyl Nitrite	0.57	0.57	0.59
DB02265	3439-38-1	Ethyl-Trimethyl-Silane	0.59	0.6	0.59
DB02532	-	2,4,6-Triaminoquinazoline	0.59	0.63	0.59
DB08671	-	5-IMINO-4-(2-TRIFLUOROMETHYL-PHENYLAZO)-5H-PYRAZOL-3-YLAMINE	0.54	0.56	0.59

DB00437	315-30-0	Allopurinol	0.54	0.56	0.58
DB07229	-	3-{5-[AMINO(IMINIO)METHYL]-1H-INDOL-2-YL}-5-METHOXY-1,1'-BIPHENYL-2-OLATE	0.51	0.52	0.58
DB08215	-	2-T-BUTYLAMINO-4-ETHYLAMINO-6-METHYLTHIO-S-TRIAZINE	0.58	0.59	0.58
DB07004	-	2-[(5-hex-1-yn-1-ylfuran-2-yl)carbonyl]-N-methylhydrazinecarbothioamide	0.51	0.54	0.58
DB04115	2086-83-1	Berberine	0.57	0.6	0.58
DB04007	-	Bromo-WR99210	0.51	0.57	0.58
DB02627	-	4,4'-Biphenyldiboronic Acid	0.61	0.6	0.58
DB07280	-	5-[4-(DIMETHYLAMINO)PHENYL]-6-[(6-MORPHOLIN-4-YLPYRIDIN-3-YL)ETHYNYL]PYRIMIDIN-4-AMINE	0.5	0.53	0.58
DB00793	777-11-7	Haloprogin	0.59	0.56	0.58
DB02091	-	4-(2,4-Dimethyl-Thiazol-5-Yl)-Pyrimidin-2-Ylamine	0.59	0.57	0.58
DB03073	-	3-Methoxybenzamide	0.57	0.61	0.58
DB03125	-	2,4-Diamino-5-(3,4,5-Trimethoxy-Benzyl)-Pyrimidin-1-Ium	0.56	0.61	0.58
DB05105	153168-05-9	Pleconaril	0.52	0.53	0.58
DB08317		5-methyl-6-phenylquinazoline-2,4-diamine	0.54	0.55	0.58
DB01996	108-99-6	3-Methylpyridine	0.66	0.52	0.58
DB08454	-	N-(5-METHYL-1H-PYRAZOL-3-YL)-2-PHENYLQUINAZOLIN-4-AMINE	0.54	0.54	0.58
DB08184	-	2-(2-METHYLPHENYL)-1H-INDOLE-5-CARBOXIMIDAMIDE	0.56	0.56	0.58
DB08075	-	4-(2-amino-1,3-thiazol-4-yl)pyrimidin-2-amine	0.55	0.55	0.58
DB02297	-	2-Amino-6-Chloropyrazine	0.55	0.59	0.58
DB07168	-	[4-({4-[(5-cyclopropyl-1H-pyrazol-3-yl)amino]-6-(methylamino)pyrimidin-2-yl}amino)phenyl]acetonitrile	0.51	0.55	0.58
DB07840	-	(E)-[4-(3,5-difluorophenyl)-3H-pyrrolo[2,3-b]pyridin-3-ylidene](3-methoxyphenyl)methanol	0.57	0.57	0.58
DB08666	-	5-imino-4-(3-trifluoromethyl-phenylazo)-5H-pyrazol-3-ylamine	0.54	0.55	0.58
DB07966	-	[4-({4-[(5-cyclopropyl-1H-pyrazol-3-yl)amino]quinazolin-2-yl}amino)phenyl]acetonitrile	0.52	0.56	0.58
DB01236	28523-86-6	Sevoflurane	0.67	0.57	0.57

DB08145	-	6-(2,6-DIMETHOXYPHENYL)PYRIDO[2,3-D]PYRIMIDINE-2,7-DIAMINE	0.6	0.63	0.57
DB04017	-	N-Methyl-N-Propargyl-3-(2,4-Dichlorophenoxy)Propylamine	0.53	0.51	0.57
DB08533	-	3-methyl-N-(pyridin-4-ylmethyl)imidazo[1,2-a]pyrazin-8-amine	0.55	0.54	0.57
DB02001	-	5-(4-Morpholin-4-Yl-Phenylsulfanyl)-2,4-Quinazolinediamine	0.53	0.51	0.57
DB03853	-	Azo-Dye Hapten	0.57	0.51	0.57
DB04254	-	8-Benzo[1,3]Dioxol-,5-Ylmethyl-9-Butyl-2-Fluoro-9h-Purin-6-Ylamine	0.52	0.55	0.57
DB05154	187235-37-6	PA824	0.54	0.53	0.57
DB00828	23155-02-4	Fosfomycin	0.55	0.55	0.57
DB02918	-	6-(4-Difluoromethoxy-3-Methoxy-Phenyl)-2h-Pyridazin-3-One	0.52	0.6	0.57
DB07073	-	5,5-dimethyl-2-morpholin-4-yl-5,6-dihydro-1,3-benzothiazol-7(4H)-one	0.5	0.53	0.57
DB00827	28657-80-9	Cinoxacin	0.55	0.59	0.57
DB08234	-	5-[3-(2,5-dimethoxyphenyl)prop-1-yn-1-yl]-6-ethylpyrimidine-2,4-diamine	0.53	0.53	0.57
DB07427	-	2-[(2-methoxy-5-methylphenoxy)methyl]pyridine	0.58	0.57	0.57
DB01918	-	[Methyltelluro]Acetate	0.56	0.5	0.57
DB00371	57-53-4	Meprobamate	0.51	0.55	0.57
DB08667	-	4-(4-fluoro-phenylazo)-5-imino-5H-pyrazol-3-ylamine	0.56	0.57	0.57
DB04251	-	Monoisopropyl Ester Phosphonic Acid Group	0.55	0.54	0.57
DB07612	-	6-(3-AMINOPHENYL)-N-(TERT-BUTYL)-2-(TRIFLUOROMETHYL)QUINAZOLIN-4-AMINE	0.54	0.56	0.56
DB08991	18694-40-1	Epirizole	0.54	0.54	0.56
DB04820	51-12-7	Nialamide	0.51	0.51	0.56
DB02372	-	2,5-Dimethylpyrimidin-4-Amine	0.6	0.56	0.56
DB01667	134-58-7	8-azaguanine	0.52	0.52	0.56
DB02170	-	1,8-Di-Hydroxy-4-Nitro-Xanthen-9-One	0.54	0.59	0.56
DB02197	-	4-[(4-Imidazo[1,2-a]Pyridin-3-Ylpyrimidin-2-Yl)Amino]Benzenesulfonamide	0.51	0.5	0.56
DB02143	-	N-Isopropyl-N'-Hydroxyguanidine	0.59	0.59	0.56
DB02984	-	4-[3-Methylsulfanylanilino]-6,7-Dimethoxyquinazoline	0.54	0.56	0.56
DB03098	-	[Methylseleno]Acetate	0.55	0.5	0.56

DB02998	965-93-5	Methyltrienolone	0.53	0.51	0.55
DB06875	-	3-(3-FLUORO-4-HYDROXYPHENYL)-7-HYDROXY-1-NAPHTHONITRILE	0.53	0.57	0.55
DB04469	-	1-(4-Methoxyphenyl)-3,5-Dimethyl-1h-Pyrazole-4-Carboxylic Acid Ethyl Ester	0.5	0.51	0.55
DB08413	-	METHYL-PHOSPHONIC ACID MONO-(4-NITRO-PHENYL) ESTER	0.5	0.51	0.55
DB00894	968-93-4	Testolactone	0.59	0.52	0.55
DB01809	-	1-Ter-Butyl-3-P-Tolyl-1h-Pyrazolo[3,4-D]Pyrimidin-4-Ylamine	0.56	0.61	0.55
DB08450	-	N-1H-indazol-5-yl-2-(6-methylpyridin-2-yl)quinazolin-4-amine	0.54	0.58	0.55
DB06781	23674-86-4	Difluprednate	0.51	0.53	0.55
DB02412	142-68-7	Tetrahydropyran	0.58	0.51	0.55
DB08359	-	2-PHENYLAMINO-4-METHYL-5-ACETYL THIAZOLE	0.52	0.53	0.55
DB03153	-	3h-Pyrazolo[4,3-D]Pyrimidin-7-Ol	0.54	0.51	0.55
DB01541	846-48-0	Boldenone	0.52	0.54	0.55
DB07245	-	6-[2-(3'-METHOXYBIPHENYL-3-YL)ETHYL]PYRIDIN-2-AMINE	0.53	0.58	0.55
DB01665	-	ZK-800270	0.52	0.52	0.55
DB08758	-	IMIDAZO[2,1-A]ISOQUINOLINE-2-CARBOHYDRAZIDE	0.51	0.54	0.54
DB00556	76-19-7	Perflutren	0.72	0.63	0.54
DB01782	-	2,6-Dihydroanthra/1,9-Cd/Pyrazol-6-One	0.57	0.55	0.54
DB04360	-	Benzo[B]Thiophene-2-Boronic Acid	0.54	0.54	0.54
DB02254	-	Trifluoro-thiamin phosphate	0.55	0.59	0.54
DB08744	-	6-methoxy-9-methyl[1,3]dioxolo[4,5-h]quinolin-8(9H)-one	0.5	0.5	0.54
DB02104	-	2,4-Diamino-5-Methyl-6-[(3,4,5-Trimethoxy-N-Methylanilino)Methyl]Pyrido[2,3-D]Pyrimidine	0.52	0.53	0.54
DB05719	488832-69-5	Elesclomol	0.52	0.56	0.54
DB04646	29096-93-3	Dibromothymoquinone	0.51	0.56	0.54
DB08386	-	2-{[4-(4-pyridin-4-yl-1H-pyrazol-3-yl)phenoxy]methyl}quinoline	0.54	0.56	0.54
DB03730	-	3,9-Dimethyladenine	0.61	0.56	0.54
DB04449	-	5-(3,3-Dihydroxypropeny)-3-Methoxy-Benzene-1,2-Diol	0.54	0.52	0.54
DB02222	-	2,6-Diamino-(S)-9-[2-(Phosphonomethoxy)Propyl]Purine	0.52	0.52	0.54
DB08214	-	4-(1H-IMIDAZOL-1-YL)PHENOL	0.55	0.55	0.54

DB01123	92-62-6	Proflavine	0.61	0.58	0.54
DB03976	-	Phosphorylisopropane	0.54	0.53	0.54
DB07249	-	N-(5-chloro-1,3-benzodioxol-4-yl)-6-methoxy-7-(3-piperidin-1-ylpropoxy)quinazolin-4-amine	0.51	0.51	0.54
DB02078	-	1-Methoxy-2-[2-(2-Methoxy-Ethoxy]-Ethane	0.56	0.56	0.54
DB03096	-	N-Aminoethylmorpholine	0.52	0.52	0.53
DB06915	-	naphthalene-1,2,4,5,7-pentol	0.51	0.53	0.53
DB03129	-	[3-(1,3,2-Dioxaborolan-2-Yloxy)Propyl]Guanidine	0.51	0.52	0.53
DB09117	123-63-7	Paraldehyde	0.5	0.52	0.53
DB08436	-	8-BENZO[1,3]DIOXOL-,5-YLMETHYL-9-BUTYL-9H-	0.52	0.54	0.53
DB03046	-	7-Methoxy-8-[1-(Methylsulfonyl)-1h-Pyrazol-4-Yl]Naphthalene-2-Carboximidamide	0.51	0.5	0.53
DB02856	-	Methyl-Carbamic Acid Ethyl Ester	0.58	0.53	0.53
DB02848	-	{4-[3-(6,7-Diethoxy-Quinazolin-4-Ylamino)-Phenyl]-Thiazol-2-Yl}-Methanol	0.51	0.55	0.53
DB03385	-	4-Methylimidazole	0.63	0.51	0.53
DB04154	-	N-Methyl-N-[3-(6-Phenyl[1,2,4]Triazolo[4,3-B]Pyridazin-3-Yl)Phenyl]Acetamide	0.53	0.51	0.53
DB07621	-	(5-(PYRIDIN-3-YL)FURAN-2-YL)METHANAMINE	0.51	0.54	0.52
DB03416	532-40-1	Thiamin Phosphate	0.53	0.57	0.52
DB03145	-	4-Methyl-5-Hydroxyethylthiazole Phosphate	0.51	0.51	0.52
DB03508	-	1-Ethoxy-2-(2-Methoxyethoxy)Ethane	0.55	0.56	0.52
DB04580	606-43-9	1-Methyl-2-quinolone	0.53	0.5	0.52
DB03506	-	9-Deazaadenine	0.58	0.58	0.52
DB08781	-	1-[(2S)-4-(5-BROMO-1H-PYRAZOLO[3,4-B]PYRIDIN-4-YL)MORPHOLIN-2-YL]METHANAMINE	0.52	0.55	0.52
DB08774	-	1-[(2S)-4-(5-phenyl-1H-pyrazolo[3,4-b]pyridin-4-yl)morpholin-2-yl]methanamine	0.51	0.53	0.52
DB03329	-	2-Pyridinethiol	0.5	0.54	0.52
DB02207	2942-42-9	7-Nitroindazole	0.59	0.57	0.52
DB08357	-	1-ETHOXY-2-(2-ETHOXYETHOXY)ETHANE	0.53	0.55	0.52
DB07959	-	3-(1H-BENZIMIDAZOL-2-YL)-1H-INDAZOLE	0.56	0.59	0.52
DB03164	-	6-Amino-1-Methylpurine	0.56	0.55	0.51

DB00276	51264-14-3	Amsacrine	0.5	0.5	0.51
DB07292	-	4-(2-amino-1,3-thiazol-4-yl)phenol	0.5	0.55	0.51
DB07821	-	(1R)-1,2,2-TRIMETHYLPROPYL (R)-METHYLPHOSPHINATE	0.53	0.55	0.51
DB07822	-	(1R)-1,2,2-TRIMETHYLPROPYL (S)-METHYLPHOSPHINATE	0.53	0.55	0.51
DB01795	-	Phenyl Boronic Acid	0.51	0.51	0.51
DB07613	-	3-phenyl-5-(1H-pyrazol-3-yl)isoxazole	0.54	0.52	0.51
DB07140	-	5-[(3R)-3-(5-methoxybiphenyl-3-yl)but-1-yn-1-yl]-6-methylpyrimidine-2,4-diamine	0.52	0.51	0.51
DB07141	-	5-[(3R)-3-(5-methoxy-4'-methylbiphenyl-3-yl)but-1-yn-1-yl]-6-methylpyrimidine-2,4-diamine	0.5	0.53	0.5
DB07935	-	5-[(5-fluoro-3-methyl-1H-indazol-4-yl)oxy]benzene-1,3-dicarbonitrile	0.57	0.54	0.5
DB06927	-	[5-HYDROXY-2-(4-HYDROXYPHENYL)-1-BENZOFURAN-7-YL]ACETONITRILE	0.51	0.58	0.5
DB07412	-	1-biphenyl-2-ylmethanamine	0.51	0.54	0.5
DB02002	-	2-Aminoprop-2-Enamide	0.58	0.53	0.5
DB07595	-	(5Z)-5-(3-BROMOCYCLOHEXA-2,5-DIEN-1-YLIDENE)-N-(PYRIDIN-4-YLMETHYL)-1,5-DIHYDROPYRAZOLO[1,5-A]PYRIMIDIN-7-AMINE	0.51	0.54	0.5

Supplementary Table S2: Detailed information for the compounds in the training dataset

Index	Name	CAS	SMILES	Class
1	Acetaminophen	103-90-2	CC(=O)NC1=CC=C(C=C1)O	Carcinogen
2	Acetohexamide	968-81-0	CC(=O)C1=CC=C(C=C1)S(=O)(=O)NC(=O)NC2CCCCC2	Non-Carcinogen
3	Acetone[4-(5-nitro-2-furyl)-2-thiazolyl]hydrazone	18523-69-8	CC(=NNC1=NC=C(S1)C2=CC=C(O2)[N+](=O)[O-])C	Carcinogen
4	Acetoxime	127-06-0	CC(=NO)C	Carcinogen
5	1'-Acetoxysafrole	34627-78-6	CC(=O)OC(C=C)C1=CC2=C(C=C1)OCO2	Carcinogen
6	4-Acetylaminobiphenyl	4075-79-0	CC(=O)NC1=CC=C(C=C1)C2=CC=CC=C2	Carcinogen
7	1-Acetylaminofluorene	28314-03-6	CC(=O)NC1=CC=CC2=C1CC3=CC=CC=C32	Non-Carcinogen
8	2-Acetylaminofluorene	53-96-3	CC(=O)NC1=CC2=C(C=C1)C3=CC=CC=C3C2	Carcinogen
9	4-Acetylaminofluorene	28322-02-3	CC(=O)NC1=CC=CC2=C1C3=CC=CC=C3C2	Non-Carcinogen
10	4-Acetylaminophenylacetic acid	18699-02-0	CC(=O)NC1=CC=C(C=C1)CC(=O)O	Non-Carcinogen
11	N-Acetylcysteine	616-91-1	CC(=O)N[C@@H](CS)C(=O)O	Non-Carcinogen
12	Acrolein	107-02-8	C=CC=O	Non-Carcinogen
13	Acrolein diethylacetal	3054-95-3	CCOC(C=C)OCC	Non-Carcinogen
14	Acrolein oxime	5314-33-0	C=C/C=N/O	Non-Carcinogen
15	Acronycine	7008-42-6	CC1(C=CC2=C3C(=C(C=C2O1)OC)C(=O)C4=CC=CC=C4N3C)C	Carcinogen
16	Acrylamide	79-06-1	C=CC(=O)N	Carcinogen
17	Acrylic acid	79-10-7	C=CC(=O)O	Non-Carcinogen
18	Acrylonitrile	107-13-1	C=CC#N	Carcinogen
19	Adipamide	628-94-4	C(CCC(=O)N)CC(=O)N	Non-Carcinogen
20	AF-2	3688-53-7	C1=COC(=C1)/C(=C/C2=CC=C(O2)[N+](=O)[O-])/C(=O)N	Carcinogen
21	Aflatoxicol	29611-03-8	COC1=C2C3=C([C@@H](CC3)O)C(=O)OC2=C4[C@@H]5C=CO[C@H]5OC4=C1	Carcinogen
22	Aflatoxin B1	1162-65-8	COC1=C2C3=C(C(=O)CC3)C(=O)OC2=C4C5C=COC5OC4=C1	Carcinogen
23	Aflatoxin G1	1165-39-5	COC1=C2C3=C(C(=O)OCC3)C(=O)OC2=C4C5C=COC5OC4=C1	Carcinogen
24	Alclofenac	22131-79-9	C=CCOC1=C(C=C(C=C1)CC(=O)O)Cl	Non-Carcinogen
25	Aldicarb	116-06-3	CC(C)(/C=N/OC(=O)NC)SC	Non-Carcinogen

26	Aldrin	309-00-2	C1[C@@H]2C=CC1[C@H]3[C@@H]2[C@]4(C(=C(C3(C4(Cl)Cl)Cl)Cl)Cl)Cl	Non-Carcinogen
27	Alkylbenzenesulfonate	42615-29-2	CCCCCCCCCCC(C)C1=CC=C(C=C1)S(=O)(=O)[O-]	Non-Carcinogen
28	Decylamine oxide	2605-79-0	CCCCCCCCCC[N+](C)(C)[O-]	Non-Carcinogen
29	Allantoin	97-59-6	C1(C(=O)NC(=O)N1)NC(=O)N	Non-Carcinogen
30	Allyl alcohol	107-18-6	C=CCO	Non-Carcinogen
31	Allyl glycidyl ether	106-92-3	C=CCOCC1CO1	Non-Carcinogen
32	Allyl isothiocyanate	57-06-7	C=CCN=C=S	Carcinogen
33	Allyl isovalerate	2835-39-4	CC(C)CC(=O)OCC=C	Carcinogen
34	1-Allyl-1-nitrosourea	760-56-5	C=CCN(C(=O)N)N=O	Carcinogen
35	1-Amino-2,4-dibromoanthraquinone	81-49-2	C1=CC=C2C(=C1)C(=O)C3=C(C2=O)C(=C(C=C3Br)Br)N	Carcinogen
36	3-Amino-4-ethoxy acetanilide	17026-81-2	CCOC1=C(C=C(C=C1)NC(=O)C)N	Non-Carcinogen
37	3-Amino-9-ethylcarbazole	6109-97-3	CCN1C2=C(C=C(C=C2)N)C3=CC=CC=C31	Carcinogen
38	3-Amino-4-[2-[(2-guanidinothiazol-4-yl)methylthio], ethylamino]-1,2,5-thiadiazole	NOCAS	C1=C(N=C(S1)N=C(N)N)CSCCNC2=NSN=C2N	Carcinogen
39	1-Amino-2-methyl anthraquinone	82-28-0	CC1=C(C2=C(C=C1)C(=O)C3=CC=CC=C3C2=O)N	Carcinogen
40	2-Amino-5-(5-nitro-2-furyl)-1,3,4-oxadiazole	3775-55-1	C1=C(OC(=C1)[N+](=O)[O-])C2=NN=C(O2)N	Carcinogen
41	2-Amino-5-(5-nitro-2-furyl)-1,3,4-thiadiazole	712-68-5	C1=C(OC(=C1)[N+](=O)[O-])C2=NN=C(S2)N	Carcinogen
42	2-Amino-4-(5-nitro-2-furyl)thiazole	38514-71-5	C1=C(OC(=C1)[N+](=O)[O-])C2=CSC(=N2)N	Carcinogen
43	2-Amino-4-nitrophenol	99-57-0	C1=CC(=C(C=C1[N+](=O)[O-])N)O	Carcinogen
44	2-Amino-5-nitrophenol	121-88-0	C1=CC(=C(C=C1[N+](=O)[O-])O)N	Carcinogen
45	4-Amino-2-nitrophenol	119-34-6	C1=CC(=C(C=C1N)[N+](=O)[O-])O	Carcinogen
46	2-Amino-5-nitrothiazole	121-66-4	C1=C(SC(=N1)N)[N+](=O)[O-]	Carcinogen

47	Tamilan	18968-99-5	C1=CC=C(C=C1)C2C(=O)N=C(O2)N	Non-Carcinogen
48	2-Aminoanthraquinone	117-79-3	C1=CC=C2C(=C1)C(=O)C3=C(C2=O)C=C(C=C3)N	Carcinogen
49	o-Aminoazotoluene	97-56-3	CC1=CC=CC=C1N=NC2=CC(=C(C=C2)N)C	Carcinogen
50	6-Aminocaproic acid	60-32-2	C(CCC(=O)O)CCN	Non-Carcinogen
51	4-Aminodiphenyl	92-67-1	C1=CC=C(C=C1)C2=CC=C(C=C2)N	Carcinogen
52	1-(Aminomethyl)cyclohexaneacetic acid	60142-96-3	C1CCC(CC1)(CC(=O)O)CN	Carcinogen
53	2,2´-[(4-Aminophenyl)imino]bisethanol	54381-16-7	C1=CC(=CC=C1N)N(CCO)CCO	Non-Carcinogen
54	11-Aminoundecanoic acid	2432-99-7	C(CCCCCN)CCCCC(=O)O	Carcinogen
55	Ammonium	3012-65-5	C(C(=O)O)C(CC(=O)[O-])(C(=O)[O-])O	Non-Carcinogen
56	Amobarbital	57-43-2	CCC1(C(=O)NC(=O)NC1=O)CCC(C)C	Non-Carcinogen
57	dl-Amphetamine	60-13-9	CC(CC1=CC=CC=C1)N	Non-Carcinogen
58	Ampicillin	7177-48-2	CC1([C@@H](N2[C@H](S1)[C@@H](C2=O)NC(=O)[C@@H](C3=CC=CC=C3)N)C(=O)O)C	Non-Carcinogen
59	1-Amyl-1-nitrosourea	10589-74-9	CCCCCN(C(=O)N)N=O	Carcinogen
60	trans-Anethole	4180-23-8	C/C=C/C1=CC=C(C=C1)OC	Non-Carcinogen
61	Anilazine	101-05-3	C1=CC=C(C(=C1)NC2=NC(=NC(=N2)Cl)Cl)Cl	Non-Carcinogen
62	o-Anisidine	134-29-2	COC1=CC=CC=C1N	Carcinogen
63	p-Anisidine	20265-97-8	COC1=CC=C(C=C1)N	Non-Carcinogen
64	Anthranilic acid	118-92-3	C1=CC=C(C(=C1)C(=O)O)N	Non-Carcinogen
65	Aramite	140-57-8	CC(COC1=CC=C(C=C1)C(C)(C)C)OS(=O)OCCCl	Carcinogen
66	Aristolochic acid	10190-99-5	COC1=CC=CC2=C3C(=C(C=C21)[N+](=O)[O-])C(=CC4=C3OCO4)C(=O)[O-]	Carcinogen
67	Aroclor 1016	12674-11-2	C1=CC(=CC(=C1)Cl)C2=CC(=CC(=C2)Cl)Cl	Carcinogen
68	Aroclor 1242	36559-22-5	C1=CC(=C(C(=C1)Cl)Cl)C2=C(C=C(C=C2)Cl)Cl	Carcinogen
69	Arochlor 1254	11097-69-1	C1=CC(=C(C(=C1)Cl)Cl)C2=C(C(=C(C=C2)Cl)Cl)Cl	Carcinogen
70	Aroclor 1260	11096-82-5	C1=CC(=C(C(=C1C2=C(C(=C(C=C2)Cl)Cl)Cl)Cl)Cl)Cl	Carcinogen
71	l-Ascorbate	134-03-2	C(C(C1C(=C(C(=O)O1)O)[O-])O)O	Non-Carcinogen
72	l-Ascorbic acid	50-81-7	C([C@@H]([C@@H]1C(=C(C(=O)O1)O)O)O)O	Non-Carcinogen
73	Aspartame	22839-47-0	COC(=O)[C@H](CC1=CC=CC=C1)NC(=O)[C@H](CC(=O)O)N	Non-Carcinogen

74	Aspirin	50-78-2	CC(=O)OC1=CC=CC=C1C(=O)O	Non-Carcinogen
75	Astemizole	68844-77-9	COC1=CC=C(C=C1)CCN2CCC(CC2)NC3=NC4=CC=CC=C4N3CC5=CC=C(C=C5)F	Non-Carcinogen
76	dl-Atenolol	51706-40-2	CC(C)NCC(COC1=CC=C(C=C1)CC(=O)N)O	Non-Carcinogen
77	Atrazine	1912-24-9	CCNC1=NC(=NC(=N1)Cl)NC(C)C	Carcinogen
78	Atropine	51-55-8	CN1[C@@H]2CC[C@H]1CC(C2)OC(=O)C(CO)C3=CC=CC=C3	Non-Carcinogen
79	Auramine-O	2465-27-2	CN(C)C1=CC=C(C=C1)C(=N)C2=CC=C(C=C2)N(C)C	Carcinogen
80	5-Azacytidine	320-67-2	C1=NC(=NC(=O)N1[C@H]2[C@@H]([C@@H]([C@H](O2)CO)O)O)N	Carcinogen
81	6-Azacytidine	3131-60-0	C1=NN(C(=O)N=C1N)[C@H]2[C@@H]([C@@H]([C@H](O2)CO)O)O	Non-Carcinogen
82	Azaserine	115-02-6	C([C@@H](C(=O)O)N)O/C(=C/[N+]#N)/[O-]	Carcinogen
83	Azathioprine	446-86-6	CN1C=NC(=C1SC2=NC=NC3=C2NC=N3)[N+](=O)[O-]	Non-Carcinogen
84	Azelnidipine	123524-52-7	CC1=C(C(C(=C(N1)N)C(=O)OC2CN(C2)C(C3=CC=CC=C3)C4=CC=CC=C4)C5=CC(=CC=C5)[N+](=O)[O-])C(=O)OC(C)C	Non-Carcinogen
85	Azinphosmethyl	86-50-0	COP(=S)(OC)SCN1C(=O)C2=CC=CC=C2N=N1	Non-Carcinogen
86	Azobenzene	103-33-3	C1=CC=C(C=C1)N=NC2=CC=CC=C2	Carcinogen
87	1-Azoxypropane	17697-55-1	CCCN=[N+](CCC)[O-]	Carcinogen
88	2-Azoxypropane	NOCAS	CC(C)N=[N+](C(C)C)[O-]	Carcinogen
89	AZT	30516-87-1	CC1=CN(C(=O)NC1=O)[C@H]2C[C@@H]([C@H](O2)CO)N=[N+]=[N-]	Carcinogen
90	Barbital	144-02-5	CCC1(C(=O)NC(=NC1=O)[O-])CC	Carcinogen
91	Barbituric acid	67-52-7	C1C(=O)NC(=O)NC1=O	Non-Carcinogen
92	Bemitradine	88133-11-3	CCOCCC1=C(N=C(N2C1=NC=N2)N)C3=CC=CC=C3	Carcinogen
93	Benzalazine	64896-26-0	C1=CC(=CC=C1C(=O)O)N/N=C/2\C=CC(=O)C(=C2)C(=O)O	Non-Carcinogen
94	Benzaldehyde	100-52-7	C1=CC=C(C=C1)C=O	Non-Carcinogen
95	Benzene	71-43-2	C1=CC=CC=C1	Carcinogen
96	Benzidine	92-87-5	C1=CC(=CC=C1C2=CC=C(C=C2)N)N	Carcinogen
97	Benzo(a)pyrene	50-32-8	C1=CC=C2C3=C4C(=CC2=C1)C=CC5=C4C(=CC=C5)C=C3	Carcinogen
98	Benzoate	532-32-1	C1=CC=C(C=C1)C(=O)[O-]	Non-Carcinogen
99	Benzofuran	271-89-6	C1=CC=C2C(=C1)C=CO2	Carcinogen
100	Benzoguanamine	91-76-9	C1=CC=C(C=C1)C2=NC(=NC(=N2)N)N	Non-Carcinogen
101	Benzoic acid	65-85-0	C1=CC=C(C=C1)C(=O)O	Non-Carcinogen
102	Benzoin	119-53-9	C1=CC=C(C=C1)C(C(=O)C2=CC=CC=C2)O	Non-Carcinogen

103	1H-Benzotriazole	95-14-7	C1=CC2=NNN=C2C=C1	Non-Carcinogen
104	Benzyl acetate	140-11-4	CC(=O)OCC1=CC=CC=C1	Non-Carcinogen
105	Benzyl alcohol	100-51-6	C1=CC=C(C=C1)CO	Non-Carcinogen
106	Benzyl chloride	100-44-7	C1=CC=C(C=C1)CCl	Non-Carcinogen
107	o-Benzyl-p-chloro phenol	120-32-1	C1=CC=C(C=C1)CC2=C(C=CC(=C2)Cl)O	Non-Carcinogen
108	Benzyl isothiocyanate	622-78-6	C1=CC=C(C=C1)CN=C=S	Non-Carcinogen
109	Benzyl thiocyanate	3012-37-1	C1=CC=C(C=C1)CSC#N	Non-Carcinogen
110	3-Benzylsydnone-4-acetamide	14504-15-5	C1=CC=C(C=C1)C[N+]2=NOC(=C2CC(=O)N)[O-]	Carcinogen
111	2-Biphenylamine	2185-92-4	C1=CC=C(C=C1)C2=CC=CC=C2N	Non-Carcinogen
112	Bis(2-chloro-1-methylethyl)ether	108-60-1	CC(CCl)OC(C)CCl	Non-Carcinogen
113	Bis(2,3-dibromopropyl)phosphate	36711-31-6	C(C(CBr)Br)OP(=O)([O-])OCC(CBr)Br	Carcinogen
114	1,4-Bis[2-(3,5-dichloropyridyloxy)]benzene	76150-91-9	C1=CC(=CC=C1OC2=C(C=C(C=N2)Cl)Cl)OC3=C(C=C(C=N3)Cl)Cl	Non-Carcinogen
115	4-Bis(2-hydroxyethyl)amino-2-(5-nitro-2-thienyl)quinazoline	33372-39-3	C1=CC=C2C(=C1)C(=NC(=N2)C3=CC=C(S3)[N+](=O)[O-])N(CCO)CCO	Carcinogen
116	4-Bis(2-hydroxyethyl)amino-2-(2-thienyl)quinazoline	58139-47-2	C1=CC=C2C(=C1)C(=NC(=N2)C3=CC=CS3)N(CCO)CCO	Non-Carcinogen
117	Bis(2-hydroxypropyl)amine	110-97-4	CC(CNCC(C)O)O	Non-Carcinogen
118	Bisphenol A	80-05-7	CC(C)(C1=CC=C(C=C1)O)C2=CC=C(C=C2)O	Non-Carcinogen
119	Black PN	2519-30-4	CC(=O)NC1=C2C(=C(C=C1)S(=O)(=O)[O-])C=C(/C(=N\NC3=C4C=C(C=CC4=C(C=C3)N=NC5=CC=C(C=C5)S(=O)(=O)[O-])S(=O)(=O)[O-])/C2=O)S(=O)(=O)[O-]	Non-Carcinogen
120	C.I. direct black 38	1937-37-7	C1=CC=C(C=C1)N/N=C/2\C(=CC3=CC(=C(C(=C3C2=O)N)N=NC4=CC=C(C=C4)C5=CC=C(C=C5)N=NC6=C(C=C(C=C6)N)N)S(=O)(=O)[O-])S(=O)(=O)[O-]	Carcinogen
121	C.I. direct blue 6	2602-46-2	C1=CC(=CC=C1C2=CC=C(C=C2)N/N=C\3/C(=CC4=CC(=CC(=C4C3=O)N)S(=O)(=O)[O-])S(=O)(=O)[O-])N/N=C/5\C(=CC6=CC(=CC(=C6C5=O)N)S(=O)(=O)[O-])S(=O)(=O)[O-]	Carcinogen
122	C.I. direct blue 15	2429-74-5	COC1=C(C=CC(=C1)C2=CC(=C(C=C2)N/N=C/3\	Carcinogen

			C(=O)C4=C(C=C(C=C4C=C3S(=O)(=O)[O-])S(=O)(=O)[O-])N)OC)N/N=C/5\C(=O)C6=C(C=C(C=C6C=C5S(=O)(=O)[O-])S(=O)(=O)[O-])N	
123	C.I. disperse blue 1	2475-45-8	C1=CC(=C2C(=C1N)C(=O)C3=C(C=CC(=C3C2=O)N)N)N	Carcinogen
124	FD & C blue no. 1	3844-45-9	CCN(CC1=CC(=CC=C1)S(=O)(=O)[O-])C2=CC=C(C=C2)C(=C3C=CC(=[N+](CC)CC4=CC(=CC=C4)S(=O)(=O)[O-])C=C3)C5=CC=CC=C5S(=O)(=O)[O-]	Non-Carcinogen
125	FD & C blue no. 2	860-22-0	C1=CC2=C(C=C1S(=O)(=O)[O-])C(=O)/C(=C\3/C(=O)C4=C(N3)C=CC(=C4)S(=O)(=O)[O-])/N2	Non-Carcinogen
126	HC blue no. 1	2784-94-3	CNC1=C(C=C(C=C1)N(CCO)CCO)[N+](=O)[O-]	Carcinogen
127	HC blue no. 2	33229-34-4	C1=CC(=C(C=C1N(CCO)CCO)[N+](=O)[O-])NCCO	Non-Carcinogen
128	Bromocriptine	22260-51-1	CC(C)C[C@H]1C(=O)N2CCC[C@H]2[C@]3(N1C(=O)[C@](O3)(C(C)C)NC(=O)[C@H]4CN([C@@H]5CC6=C(NC7=CC=CC(=C67)C5=C4)Br)C)O	Carcinogen
129	Budesonide	51333-22-3	CCC[C@@H]1O[C@@H]2C[C@H]3[C@@H]4CCC5=CC(=O)C=C[C@@]5([C@H]4[C@H](C[C@@]3([C@@]2(O1)C(=O)CO)C)O)C	Carcinogen
130	2-Butoxyethanol	111-76-2	CCCCOCCO	Non-Carcinogen
131	tert-Butyl alcohol	75-65-0	CC(C)(C)O	Carcinogen
132	n-Butyl chloride	109-69-3	CCCCCl	Non-Carcinogen
133	N-Butyl-N-(4-hydroxybutyl)nitrosamine	3817-11-6	CCCCN(CCCCO)N=O	Carcinogen
134	di-tert-Butyl-4-hydroxymethyl phenol	88-26-6	CC(C)(C)C1=CC(=CC(=C1O)C(C)(C)C)CO	Non-Carcinogen
135	2-tert-Butyl-4-methylphenol	2409-55-4	CC1=CC(=C(C=C1)O)C(C)(C)C	Non-Carcinogen
136	N-Butyl-N'-nitro-N-nitrosoguanidine	13010-08-7	CCCCN(/C(=N/[N+](=O)[O-])/N)N=O	Non-Carcinogen
137	N-n-Butyl-N-nitrosourea	869-01-2	CCCCN(C(=O)N)N=O	Carcinogen
138	Butylated hydroxyanisole	25013-16-5	CC(C)(C)C1=C(C=CC(=C1)OC)O	Carcinogen
139	Butylated hydroxytoluene	128-37-0	CC1=CC(=C(C(=C1)C(C)(C)C)O)C(C)(C)C	Non-Carcinogen
140	2,4,6-tri-tert-Butyl phenol	732-26-3	CC(C)(C)C1=CC(=C(C(=C1)C(C)(C)C)O)C(C)(C)C	Non-Carcinogen
141	p-tert-Butylphenol	98-54-4	CC(C)(C)C1=CC=C(C=C1)O	Non-Carcinogen
142	N-Butylurea	592-31-4	CCCCNC(=O)N	Non-Carcinogen

143	beta-Butyrolactone	3068-88-0	CC1CC(=O)O1	Carcinogen
144	gamma-Butyrolactone	96-48-0	C1CC(=O)OC1	Non-Carcinogen
145	Caffeic acid	331-39-5	C1=CC(=C(C=C1/C=C/C(=O)O)O)O	Carcinogen
146	Caffeine	58-08-2	CN1C=NC2=C1C(=O)N(C(=O)N2C)C	Non-Carcinogen
147	Camostat	59721-29-8	CN(C)C(=O)COC(=O)CC1=CC=C(C=C1)OC(=O)C2=CC=C(C=C2)N=C(N)N	Non-Carcinogen
148	Candesartan cilexetil	145040-37-5	CCOC1=NC2=CC=CC(=C2N1CC3=CC=C(C=C3)C4=CC=CC=C4C5=NNN=N5)C(=O)OC(C)OC(=O)OC6CCCCC6	Non-Carcinogen
149	Caprolactam	105-60-2	C1CCC(=O)NCC1	Non-Carcinogen
150	Captafol	2425-06-1	C1C=CCC2C1C(=O)N(C2=O)SC(C(Cl)Cl)(Cl)Cl	Carcinogen
151	Captan	133-06-2	C1C=CCC2C1C(=O)N(C2=O)SC(Cl)(Cl)Cl	Carcinogen
152	Carbaryl	63-25-2	CNC(=O)OC1=CC=CC2=CC=CC=C21	Carcinogen
153	Carboxymethylnitrosourea	60391-92-6	C(C(=O)O)N(C(=O)N)N=O	Carcinogen
154	Carbromal	77-65-6	CCC(CC)(C(=O)NC(=O)N)Br	Non-Carcinogen
155	b-Carotene	7235-40-7	CC1=C(C(CCC1)(C)C)/C=C/C(=C/C=C/C(=C/C=C/C=C(/C=C/C=C(/C=C/C2=C(CCCC2(C)C)C)\C)\C)/C)/C	Non-Carcinogen
156	Catechol	120-80-9	C1=CC=C(C(=C1)O)O	Carcinogen
157	Celecoxib	169590-42-5	CC1=CC=C(C=C1)C2=CC(=NN2C3=CC=C(C=C3)S(=O)(=O)N)C(F)(F)F	Non-Carcinogen
158	Celiprolol	56980-93-9	CCN(CC)C(=O)NC1=CC(=C(C=C1)OCC(CNC(C)(C)C)O)C(=O)C	Non-Carcinogen
159	Cevimeline	153504-70-2	C[C@@H]1O[C@]2(CN3CCC2CC3)CS1	Non-Carcinogen
160	Chloramben	133-90-4	C1=C(C=C(C(=C1N)Cl)C(=O)O)Cl	Non-Carcinogen
161	Chlorambucil	305-03-3	C1=CC(=CC=C1CCCC(=O)O)N(CCCl)CCCl	Carcinogen
162	Chloramphenicol	56-75-7	C1=CC(=CC=C1[C@H]([C@@H](CO)NC(=O)C(Cl)Cl)O)[N+](=O)[O-]	Non-Carcinogen
163	Chlordane	12789-03-6	C1[C@H]2[C@@H]([C@H]([C@@H]1Cl)Cl)[C@@]3(C(=C(C2(C3(Cl)Cl)Cl)Cl)Cl)Cl	Non-Carcinogen
164	Chlorendic acid	115-28-6	[C@@H]1([C@H]([C@@]2(C(=C([C@]1(C2(Cl)Cl)Cl)Cl)Cl)Cl)C(=O)O)C(=O)O	Carcinogen
165	Chlorinated paraffins	63449-39-8	CCC(CCC(C(CC(C(C(CCl)Cl)Cl)Cl)Cl)Cl)Cl	Carcinogen
166	2,5,8,11,14,17,20-heptachlorotricosane	1005111-49-8	CCCC(CCC(CCC(CCC(CCC(CCC(CCC(C)Cl)Cl)Cl)Cl)Cl)Cl)Cl	Non-Carcinogen
167	2,5,8,11,14,17,20-heptachlorotricosane	101-79-1	C1=CC(=CC=C1N)OC2=CC=C(C=C2)Cl	Carcinogen
168	3-Chloro-4-(dichloromethyl)-5-hydro	77439-76-0	C1(C(=C(C(=O)O1)Cl)C(Cl)Cl)O	Carcinogen

	xy-2(5H)-furanone			
169	2-Chloro-5-(3,5-dimethylpiperidinosulphonyl)benzoic acid	37087-94-8	C[C@@H]1C[C@@H](CN(C1)S(=O)(=O)C2=CC(=C(C=C2)Cl)C(=O)O)C	Carcinogen
170	1-Chloro-2,4-dinitrobenzene	97-00-7	C1=CC(=C(C=C1[N+](=O)[O-])[N+](=O)[O-])Cl	Non-Carcinogen
171	3-Chloro-2-methylpropene	563-47-3	CC(=C)CCl	Carcinogen
172	1-Chloro-2-nitrobenzene	88-73-3	C1=CC=C(C(=C1)[N+](=O)[O-])Cl	Non-Carcinogen
173	1-Chloro-4-nitrobenzene	100-00-5	C1=CC(=CC=C1[N+](=O)[O-])Cl	Non-Carcinogen
174	4-Chloro-m-phenylenediamine	5131-60-2	C1=CC(=C(C=C1N)N)Cl	Carcinogen
175	4-Chloro-o-phenylenediamine	95-83-0	C1=CC(=C(C=C1Cl)N)N	Carcinogen
176	2-Chloro-p-phenylenediamine	61702-44-1	C1=CC(=C(C=C1N)Cl)N	Non-Carcinogen
177	1-Chloro-2-propanol	127-00-4	CC(CCl)O	Non-Carcinogen
178	3-Chloro-p-toluidine	95-74-9	CC1=C(C=C(C=C1)N)Cl	Non-Carcinogen
179	5-Chloro-o-toluidine	95-79-4	CC1=C(C=C(C=C1)Cl)N	Non-Carcinogen
180	4-Chloro-o-toluidine	3165-93-3	CC1=C(C=CC(=C1)Cl)N	Non-Carcinogen
181	[4-Chloro-6-(2,3-xylidino)-2-pyrimidinylthio]acetic acid	50892-23-4	CC1=C(C(=CC=C1)NC2=CC(=NC(=N2)SCC(=O)O)Cl)C	Carcinogen
182	4-Chloro-6-(2,3-xylidino)-2-pyrimidinylthio(N-beta-hydroxyethyl)acetamide	65089-17-0	CC1=C(C(=CC=C1)NC2=CC(=NC(=N2)SCC(=O)NCCO)Cl)C	Carcinogen
183	2-Chloroacetophenone	532-27-4	C1=CC=C(C=C1)C(=O)CCl	Non-Carcinogen
184	4'-(Chloroacetyl)-acetanilide	140-49-8	CC(=O)NC1=CC=C(C=C1)C(=O)CCl	Non-Carcinogen
185	o-Chlorobenzalmalonitrile	2698-41-1	C1=CC=C(C(=C1)C=C(C#N)C#N)Cl	Non-Carcinogen
186	Chlorobenzene	108-90-7	C1=CC=C(C=C1)Cl	Carcinogen

187	Chlorobenzilate	510-15-6	CCOC(=O)C(C1=CC=C(C=C1)Cl)(C2=CC=C(C=C2)Cl)O	Non-Carcinogen
188	(2-Chloroethyl)trimethylammonium chloride	999-81-5	C[N+](C)(C)CCCl	Non-Carcinogen
189	1-Chloroethylnitroso-3-(2-hydroxypropyl) urea	NOCAS	CC(CNC(=O)N(C(C)Cl)N=O)O	Carcinogen
190	2-(Chloromethyl)pyridine	6959-47-3	C1=CC=NC(=C1)CCl	Non-Carcinogen
191	3-(Chloromethyl)pyridine	6959-48-4	C1=CC(=CN=C1)CCl	Carcinogen
192	3-(p-Chlorophenyl)-1,1-dimethylurea	150-68-5	CN(C)C(=O)NC1=CC=C(C=C1)Cl	Carcinogen
193	(+-)-4-(2-chlorophenyl)-2-[2-(4-isobutylphenyl)ethyl]-6,9-dimethyl-6H-thieno[3,2-f][1,2,4]triazolo[4,3-a][1,4]diazepine	117279-73-9	CC1C2=NN=C(N2C3=C(C=C(S3)CCC4=CC=C(C=C4)CC(C)C)C(=N1)C5=CC=CC=C5Cl)C	Non-Carcinogen
194	1-(4-Chlorophenyl)-1-phenyl-2-propynyl carbamate	10473-70-8	C#CC(C1=CC=CC=C1)(C2=CC=C(C=C2)Cl)OC(=O)N	Carcinogen
195	p-Chlorophenyl-2,4,5-trichlorophenyl sulfide	2227-13-6	C1=CC(=CC=C1SC2=CC(=C(C=C2Cl)Cl)Cl)Cl	Non-Carcinogen
196	Chloropicrin	76-06-2	C(#N)C1=C(C(=C(C(=C1Cl)Cl)Cl)C#N)Cl	Carcinogen
197	1-(2-chloroethyl)-1-nitroso-3-[(2S,3R,4R,5S,6R)-2,4,5-trihydroxy-6-(hydroxymethyl)oxan-3-yl]urea	NOCAS	C(CCl)N(C(=O)N[C@@H]1[C@H]([C@@H]([C@H](O[C@@H]1O)CO)O)O)N=O	Carcinogen
198	Chlorpheniramine	113-92-8	C[NH+](C)CCC(C1=CC=C(C=C1)Cl)C2=CC=CC=N2	Non-Carcinogen
199	Chlorpropamide	94-20-2	CCCNC(=O)NS(=O)(=O)C1=CC=C(C=C1)Cl	Non-Carcinogen
200	Choline chloride	67-48-1	C[N+](C)(C)CCO	Non-Carcinogen
201	Chrysazin	117-10-2	C1=CC2=C(C(=C1)O)C(=O)C3=C(C2=O)C=CC=C3O	Carcinogen
202	Cimetidine	51481-61-9	CC1=C(N=CN1)CSCCNC(=NC)NC#N	Non-Carcinogen
203	trans-Cinnamaldehyde	14371-10-9	C1=CC=C(C=C1)/C=C/C=O	Non-Carcinogen

204	Cinnamyl anthranilate	87-29-6	C1=CC=C(C=C1)/C=C/COC(=O)C2=CC=CC=C2N	Carcinogen
205	Ciprofibrate	52214-84-3	CC(C)(C(=O)O)OC1=CC=C(C=C1)C2CC2(Cl)Cl	Carcinogen
206	Citral	5392-40-5	CC(=CCC/C(=C/C=O)/C)C	Non-Carcinogen
207	Citric acid	77-92-9	C(C(=O)O)C(CC(=O)O)(C(=O)O)O	Non-Carcinogen
208	Citrinin	518-75-2	C[C@H]1[C@@H](OC=C2C1=C(C(=C(C2=O)C(=O)O)O)C)C	Carcinogen
209	Clivorine	33979-15-6	C[C@H]1/C=C(\C(=O)O[C@@H]2CC[N+]3(C2(C(=CC3)COC(=O)[C@@]1(C)OC(=O)C)[O-])C)/C=C	Carcinogen
210	Clobuzarit	22494-47-9	CC(C)(C(=O)O)OCC1=CC=C(C=C1)C2=CC=C(C=C2)Cl	Non-Carcinogen
211	Clofibrate	637-07-0	CCOC(=O)C(C)(C)OC1=CC=C(C=C1)Cl	Carcinogen
212	Clonitralid	1420-04-8	C1=CC(=C(C=C1[N+](=O)[O-])Cl)NC(=O)C2=C(C=CC(=C2)Cl)O	Non-Carcinogen
213	Clophen A 30	55600-34-5	C1=CC(=CC(=C1)Cl)C2=CC(=CC=C2)Cl	Carcinogen
214	Codeine	76-57-3	CN1CC[C@]23[C@@H]4[C@H]1CC5=C2C(=C(C=C5)OC)O[C@H]3[C@H](C=C4)O	Non-Carcinogen
215	Colcemid	477-30-5	CN[C@H]1CCC2=CC(=C(C(=C2C3=CC=C(C(=O)C=C13)OC)OC)OC)OC	Non-Carcinogen
216	Compound 50-892	65765-07-3	CC1=CC2=C(C=C1)C(=NC(=O)N2C(C)C)C3=CC(=CC=C3)O	Non-Carcinogen
217	Coumaphos	56-72-4	CCOP(=S)(OCC)OC1=CC2=C(C=C1)C(=C(C(=O)O2)Cl)C	Non-Carcinogen
218	Coumarin	91-64-5	C1=CC=C2C(=C1)C=CC(=O)O2	Carcinogen
219	m-Cresidine	102-50-1	CC1=C(C=CC(=C1)OC)N	Carcinogen
220	p-Cresidine	120-71-8	CC1=CC(=C(C=C1)OC)N	Carcinogen
221	Crotonaldehyde	123-73-9	C/C=C/C=O	Carcinogen
222	Cupferron	135-20-6	C1=CC=C(C=C1)N(N=O)[O-]	Carcinogen
223	Cyanamide	156-62-7	CCNC1=NC(=NC(=N1)Cl)NC(C)(C)C#N	Carcinogen
224	Cyclamate	139-05-9	C1CCC(CC1)NS(=O)(=O)[O-]	Non-Carcinogen
225	Cyclocytidine	31698-14-3	C1=CN2[C@H]3[C@H]([C@@H]([C@H](O3)CO)O)OC2=NC1=N	Non-Carcinogen
226	Cyclohexanone	108-94-1	C1CCC(=O)CC1	Non-Carcinogen
227	Cyclohexylamine	4998-76-9	C1CCC(CC1)N	Non-Carcinogen
228	Cyclopentanone oxime	1192-28-5	C1CCC(=NO)C1	Carcinogen
229	Piclamilast	144035-83-6	COC1=C(C=C(C=C1)C(=O)NC2=C(C=NC=C2Cl)Cl)OC3CCCC3	Carcinogen
230	Cyclophosphamide	50-18-0	C1CNP(=O)(OC1)N(CCCl)CCCl	Carcinogen
231	l-Cysteine	52-89-1	C([C@@H](C(=O)O)N)S	Non-Carcinogen

232	Cytembena	16170-75-5	COC1=CC=C(C=C1)C(=O)/C(=C\C(=O)[O-])/Br	Carcinogen
233	Dacarbazine	4342-03-4	CN(C)N/N=C\1/C(=NC=N1)C(=O)N	Carcinogen
234	Daminozide	1596-84-5	CN(C)NC(=O)CCC(=O)O	Carcinogen
235	Dapsone	80-08-0	C1=CC(=CC=C1N)S(=O)(=O)C2=CC=C(C=C2)N	Carcinogen
236	p,p'-DDD	72-54-8	C1=CC(=CC=C1C(C2=CC=C(C=C2)Cl)C(Cl)Cl)Cl	Non-Carcinogen
237	p,p'-DDE	72-55-9	C1=CC(=CC=C1C(=C(Cl)Cl)C2=CC=C(C=C2)Cl)Cl	Non-Carcinogen
238	DDT	50-29-3	C1=CC(=CC=C1C(C2=CC=C(C=C2)Cl)C(Cl)(Cl)Cl)Cl	Carcinogen
239	Deflazacort	14484-47-0	CC1=N[C@@]2([C@H](O1)C[C@@H]3[C@@]2(C[C@@H]([C@H]4[C@H]3CCC5=CC(=O)C=C[C@]45C)O)C)C(=O)COC(=O)C	Carcinogen
240	Dehydroepiandrosterone	53-43-0	C[C@]12CC[C@H]3[C@H]([C@@H]1CCC2=O)CC=C4[C@@]3(CC[C@@H](C4)O)C	Carcinogen
241	Piclamilast	853-23-6	CC(=O)O[C@H]1CC[C@@]2([C@H]3CC[C@]4([C@H]([C@@H]3CC=C2C1)CCC4=O)C)C	Carcinogen
242	Deltamethrin	52918-63-5	CC1([C@H]([C@H]1C(=O)O[C@H](C#N)C2=CC(=CC=C2)OC3=CC=CC=C3)C=C(Br)Br)C	Non-Carcinogen
243	Deserpidine	131-01-1	CO[C@H]1[C@@H](C[C@@H]2CN3CCC4=C([C@H]3C[C@@H]2[C@@H]1C(=O)OC)NC5=CC=CC=C45)OC(=O)C6=CC(=C(C(=C6)OC)OC)OC	Non-Carcinogen
244	Dexamethazone	50-02-2	C[C@@H]1C[C@H]2[C@@H]3CCC4=CC(=O)C=C[C@@]4([C@]3([C@H](C[C@@]2([C@]1(C(=O)CO)O)C)O)F)C	Non-Carcinogen
245	N-1-Diacetamidofluorene	63019-65-8	CC(=O)N(C1=CC=CC2=C1CC3=CC=CC=C32)C(=O)C	Carcinogen
246	Diallyl phthalate	131-17-9	C=CCOC(=O)C1=CC=CC=C1C(=O)OCC=C	Non-Carcinogen
247	Diallylnitrosamine	16338-97-9	C=CCN(CC=C)N=O	Carcinogen
248	4,6-Diamino-2-(5-nitro-2-furyl)-S-triazine	720-69-4	C1=C(OC(=C1)[N+](=O)[O-])C2=NC(=NC(=N2)N)N	Carcinogen
249	4,4'-Diamino-2,2'-stilbenedisulfonic acid	7336-20-1	C1=CC(=C(C=C1N)S(=O)(=O)[O-])/C=C/C2=C(C=C(C=C2)N)S(=O)(=O)[O-]	Non-Carcinogen
250	2,4-Diaminoanisole	39156-41-7	COC1=C(C=C(C=C1)N)N	Carcinogen
251	2,4-Diaminophenol	137-09-7	C1=CC(=C(C=C1N)N)O	Non-Carcinogen
252	2,4-Diaminotoluene	95-80-7	CC1=C(C=C(C=C1)N)N	Carcinogen
253	2,6-Diaminotoluene	15481-70-6	CC1=C(C=CC=C1N)N	Non-Carcinogen

254	2,5-Diaminotoluene	6369-59-1	CC1=C(C=CC(=C1)N)N	Non-Carcinogen
255	Diazepam	439-14-5	CN1C(=O)CN=C(C2=C1C=CC(=C2)Cl)C3=CC=CC=C3	Non-Carcinogen
256	Diazinon	333-41-5	CCOP(=S)(OCC)OC1=NC(=NC(=C1)C)C(C)C	Non-Carcinogen
257	3-Diazotyramine	NOCAS	C1=CC(=C(C=C1CCN)[N+]#N)[O-]	Carcinogen
258	Dibenzo-p-dioxin	262-12-4	C1=CC=C2C(=C1)OC3=CC=CC=C3O2	Non-Carcinogen
259	3-Dibenzofuranamine	4106-66-5	C1=CC=C2C(=C1)C3=C(O2)C=C(C=C3)N	Carcinogen
260	O,S-Dibenzoyl thiamine	35660-60-7	CC1=NC=C(C(=N1)N)CN(C=O)/C(=C(/CCOC(=O)C2=CC=CC=C2)\SC(=O)C3=CC=CC=C3)/C	Non-Carcinogen
261	1,2-Dibromo-3-chloropropane	96-12-8	C(C(CBr)Br)Cl	Carcinogen
262	Dibromodulcitol	10318-26-0	C([C@H]([C@@H]([C@@H]([C@H](CBr)O)O)O)O)Br	Carcinogen
263	Dibromomannitol	488-41-5	C([C@@H]([C@H]([C@@H]([C@H](CBr)O)O)O)O)Br	Carcinogen
264	5,7-Dibromoquinoline	34522-69-5	C1=CC2=C(C=C(C=C2N=C1)Br)Br	Non-Carcinogen
265	1,3-Dibutyl-1-nitrosourea	56654-52-5	CCCCNC(=O)N(CCCC)N=O	Carcinogen
266	Dibutyltin diacetate	1067-33-0	CCCC[Sn](CCCC)(OC(=O)C)OC(=O)C	Non-Carcinogen
267	2,6-Dichloro-p-phenylenediamine	609-20-1	C1=C(C=C(C(=C1Cl)N)Cl)N	Non-Carcinogen
268	1,2-Dichlorobenzene	95-50-1	C1=CC=C(C(=C1)Cl)Cl	Non-Carcinogen
269	1,4-Dichlorobenzene	106-46-7	C1=CC(=CC=C1Cl)Cl	Carcinogen
270	3,3'-Dichlorobenzidine	91-94-1	C1=CC(=C(C=C1C2=CC(=C(C=C2)N)Cl)Cl)N	Carcinogen
271	trans-1,4-Dichlorobutene-2	110-57-6	C(/C=C/CCl)Cl	Carcinogen
272	2,7-Dichlorodibenzo-p-dioxin	33857-26-0	C1=CC2=C(C=C1Cl)OC3=C(O2)C=C(C=C3)Cl	Non-Carcinogen
273	p,p'-Dichlorodiphenyl sulfone	80-07-9	C1=CC(=CC=C1S(=O)(=O)C2=CC=C(C=C2)Cl)Cl	Non-Carcinogen
274	2,4-Dichlorophenol	120-83-2	C1=CC(=C(C=C1Cl)Cl)O	Non-Carcinogen
275	2,4-Dichlorophenoxyacetic acid	94-75-7	C1=CC(=C(C=C1Cl)Cl)OCC(=O)O	Non-Carcinogen
276	2,4-Dichlorópheno	94-80-4	CC(CCl)Cl	Non-Carcinogen

	xyacetic acid, n-butyl ester			
277	Dichlorvos	62-73-7	COP(=O)(OC)OC=C(Cl)Cl	Carcinogen
278	Dicofol	115-32-2	C1=CC(=CC=C1C(C2=CC=C(C=C2)Cl)(C(Cl)(Cl)Cl)O)Cl	Non-Carcinogen
279	N,N'-Dicyclohexyl thiourea	1212-29-9	C1CCC(CC1)NC(=S)NC2CCCCC2	Non-Carcinogen
280	Dicyclopentadiene dioxide	81-21-0	C1C2C3CC4C(C3C1C5C2O5)O4	Non-Carcinogen
281	Dieldrin	60-57-1	C1[C@@H]2[C@H]3[C@@H]([C@H]1[C@H]4[C@@H]2O4)[C@]5(C(=C([C@@]3(C5(Cl)Cl)Cl)Cl)Cl)Cl	Non-Carcinogen
282	Dieldrin, photo-	13366-73-9	C12C3C(C4C1C5(C(C3(C4(C5(Cl)Cl)Cl)Cl)Cl)Cl)C6C2O6	Non-Carcinogen
283	d,l-Diepoxybutane	298-18-0	C1C(O1)C2CO2	Non-Carcinogen
284	N,N-Diethyl-4-(4'-[pyridyl-1'-oxide]azo)aniline	7347-49-1	CCN(CC)C1=CC=C(C=C1)N=NC2=CC=[N+](C=C2)[O-]	Carcinogen
285	N,N-Diethyl-m-toluamide	134-62-3	CCN(CC)C(=O)C1=CC(=CC=C1)C	Non-Carcinogen
286	O,O-Diethyl-O-(3,5,6-trichloro-2-pyridyl)phosphorothiolate	2921-88-2	CCOP(=S)(OCC)OC1=NC(=C(C=C1Cl)Cl)Cl	Non-Carcinogen
287	Diethylacetamide	685-91-6	CCN(CC)C(=O)C	Carcinogen
288	Diethylacetylurea	NOCAS	CCC(CC)C(=O)NC(=O)N	Non-Carcinogen
289	(+-)-4-diethylamino-1,1-dimethylbut-2-yn-1-yl 2-cyclohexyl-2-hydroxy-2-phenylacetate	NOCAS	CCN(CC)CC#CC(C)(C)OC(=O)C(C1CCCCC1)(C2=CC=CC=C2)O	Non-Carcinogen
290	Diethylene glycol	111-46-6	C(COCCO)O	Carcinogen
291	Diethylformamide	617-84-5	CCN(CC)C=O	Non-Carcinogen
292	Diethylmaleate	141-05-9	CCOC(=O)/C=C\C(=O)OCC	Non-Carcinogen
293	Diethylstilbestrol	56-53-1	CC/C(=C(/CC)\C1=CC=C(C=C1)O)/C2=CC=C(C=C2)O	Carcinogen
294	N,N'-Diethylthiourea	105-55-5	CCNC(=S)NCC	Carcinogen
295	2-(Difluoromethyl)-dl-ornithine	70052-12-9	C(CC(C(F)F)(C(=O)O)N)CN	Non-Carcinogen
296	Diglycidyl	101-90-6	C1C(O1)COC2=CC(=CC=C2)OCC3CO3	Carcinogen

	resorcinol ether			
297	5,6-Dihydro-5-azacytidine	62488-57-7	C1N=C(NC(=O)N1[C@H]2[C@@H]([C@@H]([C@H](O2)CO)O)O)N	Non-Carcinogen
298	1,2-Dihydro-2-(5-nitro-2-thienyl) quinazolin-4(3H)-one	33389-33-2	C1=CC=C2C(=C1)C(=O)NC(N2)C3=CC=C(S3)[N+](=O)[O-]	Carcinogen
299	3,6-Dihydro-2-nitroso-2H-1,2-oxazine	3276-41-3	C1C=CCON1N=O	Carcinogen
300	3,4-Dihydrocoumarin	119-84-6	C1CC(=O)OC2=CC=CC=C21	Carcinogen
301	Dihydrosafrole	94-58-6	CCCC1=CC2=C(C=C1)OCO2	Carcinogen
302	3,3'-Dihydroxybenzidine	1592-36-5	C[C@H](CC[C@H](C(C)(C)O)O)[C@H]1CC[C@@H]\2[C@@]1(CCC/C2=C\C=C/3\C[C@H](CCC3=C)O)C	Non-Carcinogen
303	Diisononyl phthalate	68515-48-0	CC(C)CCCCCCOC(=O)C1=CC=CC=C1C(=O)OCCCCCCC(C)C	Carcinogen
304	(R,R)-Dilevalol	75659-08-4	C[C@H](CCC1=CC=CC=C1)NC[C@@H](C2=CC(=C(C=C2)O)C(=O)N)O	Non-Carcinogen
305	Dimethadione	695-53-4	CC1(C(=O)NC(=O)O1)C	Non-Carcinogen
306	Dimethoate	60-51-5	CNC(=O)CSP(=S)(OC)OC	Non-Carcinogen
307	2,5-Dimethoxy-4'-aminostilbene	5803-51-0	COC1=CC(=C(C=C1)OC)/C=C/C2=CC=C(C=C2)N	Carcinogen
308	2,4-Dimethoxyaniline	54150-69-5	COC1=CC(=C(C=C1)N)OC	Non-Carcinogen
309	3,3'-Dimethoxybenzidine-4,4'-diisocyanate	91-93-0	COC1=C(C=CC(=C1)C2=CC(=C(C=C2)N=C=O)OC)N=C=O	Carcinogen
310	3,3'-Dimethoxybenzidine	20325-40-0	COC1=C(C=CC(=C1)C2=CC(=C(C=C2)N)OC)N	Carcinogen
311	5,7-Dimethoxycyclopentene[c]coumarin	1146-71-0	COC1=CC(=C2C3=C(CCC3)C(=O)OC2=C1)OC	Non-Carcinogen
312	5,7-Dimethoxycyclopentenone[2,3-c]coumarin	1150-37-4	COC1=CC(=C2C(=C1)OC(=O)C3=C2C(=O)CC3)OC	Non-Carcinogen
313	5,7-Dimethoxycyclopentenone[3,2-c] coumarin	1150-42-1	COC1=CC(=C2C3=C(C(=O)CC3)C(=O)OC2=C1)OC	Non-Carcinogen
314	5,6-Dimethoxysterigmatocystin	65176-75-2	COC1=C(C2=C(C(=C1)O)C(=O)C3=C(C=C4C(=C3O2)C5C=COC5O4)OC)OC	Carcinogen

315	O,O-Dimethyl S-2(acetylamino)ethyl dithiophosphate	13265-60-6	CC(=O)NCCSP(=S)(OC)OC	Non-Carcinogen
316	N,N-Dimethyl-4-aminoazobenzene	60-11-7	CN(C)C1=CC=C(C=C1)N=NC2=CC=CC=C2	Carcinogen
317	N,N'-Dimethyl-N,N'-dinitrosophthalamide	3851-16-9	CN(C(=O)C1=CC=CC=C1C(=O)N(C)N=O)N=O	Non-Carcinogen
318	Dimethyl methylphosphonate	756-79-6	COP(=O)(C)OC	Carcinogen
319	Dimethyl morpholinophosphoramidate	597-25-1	COP(=O)(N1CCOCC1)OC	Carcinogen
320	4,6-Dimethyl-2-(5-nitro-2-furyl) pyrimidine	59-35-8	CC1=CC(=NC(=N1)C2=CC=C(O2)[N+](=O)[O-])C	Carcinogen
321	1,2-Dimethyl-5-nitroimidazole	551-92-8	CC1=NC=C(N1C)[N+](=O)[O-]	Carcinogen
322	Dimethyl terephthalate	120-61-6	COC(=O)C1=CC=C(C=C1)C(=O)OC	Non-Carcinogen
323	Dimethylacetamide	127-19-5	CC(=O)N(C)C	Non-Carcinogen
324	6-Dimethylamino-4,4-diphenyl-3-heptanolacetate	43033-72-3	CC[C@@H](C(C[C@H](C)N(C)C)(C1=CC=CC=C1)C2=CC=CC=C2)OC(=O)C	Carcinogen
325	6-Dimethylamino-4,4-diphenyl-3-heptanone	1095-90-5	CCC(=O)C(CC(C)N(C)C)(C1=CC=CC=C1)C2=CC=CC=C2	Non-Carcinogen
326	trans-2-[(Dimethylamino)methylimino]-5-[2-(5-nitro-2-furyl)vinyl]-1,3,4-oxadiazole	55738-54-0	CN(C)CNC1=NN=C(O1)/C=C/C2=CC=C(O2)[N+](=O)[O-]	Carcinogen
327	Dimethylaminoethylnitrosoethylurea, nitrite salt	142713-78-8	CC[NH2+]C(=O)N(CCN(C)C)N=O	Carcinogen
328	2,6-Dimethylaniline	87-62-7	CC1=C(C(=CC=C1)C)N	Non-Carcinogen
329	N,N-Dimethylaniline	121-69-7	CN(C)C1=CC=CC=C1	Carcinogen
330	Dimethylarsinic acid	75-60-5	CC1(C(=O)NC(=O)NC1=O)C	Non-Carcinogen
331	3,3'-Dimethylbenzi	612-82-8	CC1=C(C=CC(=C1)C2=CC(=C(C=C2)N)C)N	Carcinogen

	dine			
332	N,N-Dimethyldodecylamine-N-oxide	1643-20-5	CCCCCCCCCCCC[N+](C)(C)[O-]	Non-Carcinogen
333	Dimethylformamide	68-12-2	CN(C)C=O	Non-Carcinogen
334	2-(2,2-Dimethylhydrazino)-4-(5-nitro-2-furyl)thiazole	26049-69-4	CN(C)NC1=NC(=CS1)C2=CC=C(O2)[N+](=O)[O-]	Carcinogen
335	1,3-Dimethylthiourea	534-13-4	CNC(=S)NC	Non-Carcinogen
336	Dimethylvinyl chloride	513-37-1	CC(=CCl)C	Carcinogen
337	2,4-Dinitro-6-tert-butylphenylmethanesulfonate	29110-68-7	CC(C)(C)C1=CC(=CC(=C1OS(=O)(=O)C)[N+](=O)[O-])[N+](=O)[O-]	Non-Carcinogen
338	Dinitrosocaffeidine	145438-97-7	CN1C=NC(=C1C(=O)N(C)N=O)N(C)N=O	Carcinogen
339	Dinitrosohomopiperazine	55557-00-1	C1CN(CCN(C1)N=O)N=O	Carcinogen
340	N,N-Dinitrosopentamethylenetetramine	101-25-7	C1N2CN(CN1CN(C2)N=O)N=O	Non-Carcinogen
341	2,6-Dinitrotoluene	606-20-2	CC1=C(C=CC=C1[N+](=O)[O-])[N+](=O)[O-]	Carcinogen
342	1,4-Dioxane	123-91-1	C1COCCO1	Carcinogen
343	Dioxathion	78-34-2	CCOP(=S)(OCC)SC1C(OCCO1)SP(=S)(OCC)OCC	Non-Carcinogen
344	Dipentylnitrosamine	13256-06-9	CCCCCN(CCCCC)N=O	Carcinogen
345	Diphenhydramine	147-24-0	CN(C)CCOC(C1=CC=CC=C1)C2=CC=CC=C2	Non-Carcinogen
346	Diphenyl-p-phenylenediamine	74-31-7	C1=CC=C(C=C1)NC2=CC=C(C=C2)NC3=CC=CC=C3	Non-Carcinogen
347	5,5-Diphenylhydantoin	57-41-0	C1=CC=C(C=C1)C2(C(=O)NC(=O)N2)C3=CC=CC=C3	Non-Carcinogen
348	N,N-Dipropyl-4-(4'-[pyridyl-1'-oxide]azo)aniline	NOCAS	CCCN(CCC)C1=CC=C(C=C1)N=NC2=CC=[N+](C=C2)[O-]	Non-Carcinogen
349	Dipropylene glycol	25265-71-8	CCC(O)OC(CC)O	Non-Carcinogen
350	Dipyrone	68-89-3	CC1=C(C(=O)N(N1C)C2=CC=CC=C2)N(C)CS(=O)(=O)[O-]	Non-Carcinogen
351	Disodium 5´-ribonucleotide	80702-47-2	C1=NC(=O)C2=C(N1)N(C=N2)[C@H]3[C@@H]([C@@H]([C@H](O3)COP(=O)([O-])[O-])O)O	Non-Carcinogen
352	3-O-Dodecylcarbomethylascorbic	NOCAS	CCCCCCCCCCCCC(=O)COC1=C(C(=O)O[C@@H]1[C@H](CO)O)O	Non-Carcinogen

	acid			
353	dl-Dopa	63-84-3	C1=CC(=C(C=C1CC(C(=O)O)N)O)O	Non-Carcinogen
354	Dopamine	62-31-7	C1=CC(=C(C=C1CCN)O)O	Non-Carcinogen
355	Doxefazepam	40762-15-0	C1=CC=C(C(=C1)C2=NC(C(=O)N(C3=C2C=C(C=C3)Cl)CCO)O)F	Non-Carcinogen
356	Doxylamine succinate	562-10-7	CC(C1=CC=CC=C1)(C2=CC=CC=N2)OCCN(C)C	Carcinogen
357	EDTA	150-38-9	C(CN(CC(=O)[O-])CC(=O)[O-])N(CC(=O)O)CC(=O)[O-]	Non-Carcinogen
358	Efonidipine	111011-76-8	CC1=C(C(C(=C(N1)C)P2(=O)OCC(CO2)(C)C)C3=CC(=CC=C3)[N+](=O)[O-])C(=O)OCCN(CC4=CC=CC=C4)C5=CC=CC=C5	Non-Carcinogen
359	Ellagic acid	476-66-4	C1=C2C3=C(C(=C1O)O)OC(=O)C4=CC(=C(C(=C43)OC2=O)O)O	Non-Carcinogen
360	Emodin	518-82-1	CC1=CC(=C2C(=C1)C(=O)C3=CC(=CC(=C3C2=O)O)O)O	Non-Carcinogen
361	Endosulfan	115-29-7	C1C2C(COS(=O)O1)C3(C(=C(C2(C3(Cl)Cl)Cl)Cl)Cl)Cl	Non-Carcinogen
362	Endrin	72-20-8	C1[C@@H]2[C@@H]3[C@H]([C@H]1[C@H]4[C@@H]2O4)[C@@]5(C(=C([C@]3(C5(Cl)Cl)Cl)Cl)Cl)Cl	Non-Carcinogen
363	Ephedrine	134-72-5	C[C@@H]([C@@H](C1=CC=CC=C1)O)NC	Non-Carcinogen
364	Epichlorohydrin	106-89-8	C1C(O1)CCl	Carcinogen
365	1,2-Epoxybutane	106-88-7	CCC1CO1	Carcinogen
366	Erythorbate	6381-77-7	C([C@H]([C@@H]1C(=C(C(=O)O1)O)O)O)[O-]	Non-Carcinogen
367	Erythromycin	643-22-1	CC[C@@H]1[C@@]([C@@H]([C@H](C(=O)[C@@H](C[C@@]([C@@H]([C@H]([C@@H]([C@H](C(=O)O1)C)O[C@H]2C[C@@]([C@H]([C@@H](O2)C)O)(C)OC)C)O[C@H]3[C@@H]([C@H](C[C@H](O3)C)[NH+](C)C)O)(C)O)C)C)O)(C)O	Non-Carcinogen
368	Estazolam	29975-16-4	C1C2=NN=CN2C3=C(C=C(C=C3)Cl)C(=N1)C4=CC=CC=C4	Non-Carcinogen
369	Estradiol	50-28-2	C[C@]12CC[C@H]3[C@H]([C@@H]1CC[C@@H]2OC(=O)CC4=CC=C(C=C4)N(CCCl)CCCl)CCC5=C3C=CC(=C5)OC(=O)CC6=CC=C(C=C6)N(CCCl)CCCl	Non-Carcinogen
370	Ethinyl estradiol	57-63-6	C[C@]12CC[C@H]3[C@H]([C@@H]1CC[C@]2(C#C)O)CCC4=C3C=CC(=C4)O	Carcinogen
371	Ethionamide	536-33-4	CCC1=NC=CC(=C1)C(=S)N	Non-Carcinogen
372	Ethionine	13073-35-3	CCSCC[C@@H](C(=O)O)N	Carcinogen
373	dl-Ethionine	67-21-0	CCSCCC(C(=O)O)N	Carcinogen
374	4-Ethoxy-phenylur	150-69-6	CCOC1=CC=C(C=C1)NC(=O)N	Carcinogen

	ea			
375	Ethoxyquin	91-53-2	CCOC1=CC2=C(C=C1)NC(C=C2C)(C)C	Non-Carcinogen
376	Ethyl acrylate	140-88-5	CCOC(=O)C=C	Carcinogen
377	Z-Ethyl-O,N,N-azoxyethane	16301-26-1	CCN=[N+](CC)[O-]	Carcinogen
378	Z-Ethyl-O,N,N-azoxymethane	57497-29-7	CC[N+](=NC)[O-]	Carcinogen
379	S-Ethyl-l-cysteine	2629-59-6	CCSC[C@@H](C(=O)O)N	Non-Carcinogen
380	p,p'-Ethyl-DDD	72-56-0	CCC1=CC=C(C=C1)C(C2=CC=C(C=C2)CC)C(Cl)Cl	Non-Carcinogen
381	Ethyl methylphenylglycidate	77-83-8	CCOC(=O)C1C(O1)(C)C2=CC=CC=C2	Non-Carcinogen
382	1-Ethyl-1-nitrosourea	759-73-9	CCN(C(=O)N)N=O	Carcinogen
383	3-O-Ethylascorbic acid	86404-04-8	CCOC1=C(C(=O)O[C@@H]1[C@H](CO)O)O	Non-Carcinogen
384	Ethylbenzene	100-41-4	CCC1=CC=CC=C1	Carcinogen
385	Ethylene thiourea	96-45-7	C1CNC(=S)N1	Carcinogen
386	1-Ethyleneoxy-3,4-epoxycyclohexane	106-87-6	C1CC2C(O2)CC1C3CO3	Non-Carcinogen
387	2-Ethylhexanol	104-76-7	CCCCC(CC)CO	Non-Carcinogen
388	di(2-Ethylhexyl)adipate	103-23-1	CCCCC(CC)COC(=O)CCCCC(=O)OCC(CC)CCCC	Non-Carcinogen
389	di(2-Ethylhexyl)phthalate	117-81-7	CCCCC(CC)COC(=O)C1=CC=CC=C1C(=O)OCC(CC)CCCC	Carcinogen
390	1-Ethylnitroso-3-(2-hydroxyethyl)-urea	96724-44-6	CCN(C(=O)NCCO)N=O	Carcinogen
391	1-Ethylnitroso-3-(2-oxopropyl)-urea	110559-84-7	CCN(C(=O)NCC(=O)C)N=O	Carcinogen
392	Ethylnitrosocyanamide	38434-77-4	CCN(C#N)N=O	Carcinogen
393	Ethylphenylacetylurea	90-49-3	CCC(C1=CC=CC=C1)C(=O)NC(=O)N	Non-Carcinogen
394	Ethynodiol diacetate	297-76-7	CC(=O)O[C@H]1CC[C@@H]2[C@H]3CC[C@]4([C@H]([C@@H]3CCC2=C1)CC[C@]4(C#C)OC(=O)C)C	Non-Carcinogen
395	Etodolac	41340-25-4	CCC1=CC=CC2=C1NC3=C2CCOC3(CC)CC(=O)O	Non-Carcinogen
396	Eugenol	97-53-0	COC1=C(C=CC(=C1)CC=C)O	Non-Carcinogen
397	Fadrozole	102676-31-3	C1CC(N2C=NC=C2C1)C3=CC=C(C=C3)C#N	Non-Carcinogen

398	Fenaminosulf	140-56-7	CN(C)C1=CC=C(C=C1)N=NS(=O)(=O)[O-]	Non-Carcinogen
399	Fenthion	55-38-9	CC1=C(C=CC(=C1)OP(=S)(OC)OC)SC	Non-Carcinogen
400	Fenvalerate	51630-58-1	CC(C)C(C1=CC=C(C=C1)Cl)C(=O)OC(C#N)C2=CC(=CC=C2)OC3=CC=CC=C3	Non-Carcinogen
401	Flecainide acetate	54143-56-5	C1CCNC(C1)CNC(=O)C2=C(C=CC(=C2)OCC(F)(F)F)OCC(F)(F)F	Non-Carcinogen
402	Fluconazole	86386-73-4	C1=CC(=C(C=C1F)F)C(CN2C=NC=N2)(CN3C=NC=N3)O	Carcinogen
403	Fluometuron	2164-17-2	CN(C)C(=O)NC1=CC=CC(=C1)C(F)(F)F	Non-Carcinogen
404	N-(2-Fluorenyl)-2,2,2-trifluoroacetamide	363-17-7	C1C2=CC=CC=C2C3=C1C=C(C=C3)NC(=O)C(F)(F)F	Carcinogen
405	N-4-(4'-Fluorobiphenyl)acetamide	398-32-3	CC(=O)NC1=CC=C(C=C1)C2=CC=C(C=C2)F	Carcinogen
406	2-Fluoroethyl-nitrosourea	69112-98-7	C(CF)N(C(=O)N)N=O	Carcinogen
407	5-Fluorouracil	51-21-8	C1=C(C(=O)NC(=O)N1)F	Non-Carcinogen
408	Fluoxetine	59333-67-4	CNCCC(C1=CC=CC=C1)OC2=CC=C(C=C2)C(F)(F)F	Non-Carcinogen
409	Fluvastatin	93957-54-1	CC(C)N1C2=CC=CC=C2C(=C1/C=C/[C@H](C[C@H](CC(=O)O)O)O)C3=CC=C(C=C3)F	Carcinogen
410	Formic acid 2-[4-(2-furyl)-2-thiazolyl]hydrazide	31873-81-1	C1=COC(=C1)C2=CSC(=N2)NNC=O	Non-Carcinogen
411	Formic acid 2-(4-methyl-2-thiazolyl)hydrazide	32852-21-4	CC1=CSC(=N1)NNC=O	Carcinogen
412	Formic acid 2-[4-(5-nitro-2-furyl)-2-thiazolyl]hydrazide	3570-75-0	C1=C(OC(=C1)[N+](=O)[O-])C2=CSC(=N2)NNC=O	Carcinogen
413	Fumonisin B1	116355-83-0	CCCC[C@H](C)[C@H]([C@@H](C[C@H](C)C[C@H](CCCC[C@H](C[C@@H]([C@H](C)N)O)O)O)OC(=O)C[C@H](CC(=O)O)C(=O)O)OC(=O)C[C@H](CC(=O)O)C(=O)O	Carcinogen
414	2-Furaldehyde semicarbazone	2411-74-7	C1=COC(=C1)/C=N/NC(=O)N	Non-Carcinogen
415	Furan	110-00-9	C1=COC=C1	Carcinogen
416	Furfural	98-01-1	C1=COC(=C1)C=O	Carcinogen
417	Furfuryl alcohol	98-00-0	C1=COC(=C1)CO	Carcinogen
418	Furosemide	54-31-9	C1=COC(=C1)CNC2=CC(=C(C=C2C(=O)O)S(=O)(=O)N)Cl	Non-Carcinogen

419	Fusarenon-X	23255-69-8	CC1=CC2[C@@](C(C1=O)O)([C@]3([C@@H]([C@H](C([C@@]34CO4)O2)O)OC(=O)C)C)CO	Non-Carcinogen
420	Gallic acid	149-91-7	C1=C(C=C(C(=C1O)O)O)C(=O)O	Non-Carcinogen
421	Gemcadiol	35449-36-6	CC(C)(CCCCCCC(C)(C)CO)CO	Non-Carcinogen
422	Gemfibrozil	25812-30-0	CC1=CC(=C(C=C1)C)OCCCC(C)(C)C(=O)O	Carcinogen
423	Geranyl acetate	NOCAS	CC(=CCC/C(=C/COC(=O)C)/C)C	Non-Carcinogen
424	Glu-P-1	67730-11-4	CC1=CC=CN2C1=NC3=C2N=C(C=C3)N	Carcinogen
425	Glu-P-2	67730-10-3	C1=CC2=NC3=C(N2C=C1)N=C(C=C3)N	Carcinogen
426	Glutaraldehyde	111-30-8	C(CC=O)CC=O	Non-Carcinogen
427	Glycerol alpha-monochlorohydrin	96-24-2	C(C(CCl)O)O	Non-Carcinogen
428	Glycidaldehyde	765-34-4	C1C(O1)C=O	Non-Carcinogen
429	Glycidol	556-52-5	C1C(O1)CO	Carcinogen
430	Glycyrrhetinic acid	471-53-4	C[C@]12CC[C@](C[C@H]1C3=CC(=O)[C@@H]4[C@]5(CC[C@@H](C([C@@H]5CC[C@]4([C@@]3(CC2)C)C)(C)C)O)C)(C)C(=O)O	Non-Carcinogen
431	FD & C green no. 1	4680-78-8	CCN(CC1=CC(=CC=C1)S(=O)(=O)[O-])C2=CC=C(C=C2)C(=C3C=CC(=[N+](CC)CC4=CC(=CC=C4)S(=O)(=O)[O-])C=C3)C5=CC=CC=C5	Carcinogen
432	FD & C green no. 2	5141-20-8	CCN(CC1=CC(=CC=C1)S(=O)(=O)[O-])C2=CC=C(C=C2)C(=C3C=CC(=[N+](CC)CC4=CC(=CC=C4)S(=O)(=O)[O-])C=C3)C5=CC=C(C=C5)S(=O)(=O)[O-]	Carcinogen
433	FD & C green no. 3	2353-45-9	CCN(CC1=CC(=CC=C1)S(=O)(=O)[O-])C2=CC=C(C=C2)C(=C3C=CC(=[N+](CC)CC4=CC(=CC=C4)S(=O)(=O)[O-])C=C3)C5=C(C=C(C=C5)O)S(=O)(=O)[O-]	Non-Carcinogen
434	1,2,3,7,8,9-Hexachlorodibenzodioxin	19408-74-3	C1=C2C(=C(C(=C1Cl)Cl)Cl)OC3=C(C(=C(C=C3O2)Cl)Cl)Cl	Carcinogen
435	Hematoxylin	517-28-2	C1C2=CC(=C(C=C2[C@@H]3[C@]1(COC4=C3C=CC(=C4O)O)O)O)O	Carcinogen
436	Heptachlor	76-44-8	C1=CC(C2C1C3(C(=C(C2(C3(Cl)Cl)Cl)Cl)Cl)Cl)Cl	Non-Carcinogen
437	Heptamethyleneimine	1121-92-2	C1CCCNCCC1	Non-Carcinogen
438	Heptylamine	111-68-2	CCCCCCCN	Non-Carcinogen
439	Hexachlorobenzene	118-74-1	C1(=C(C(=C(C(=C1Cl)Cl)Cl)Cl)Cl)Cl	Carcinogen
440	Hexachlorobutadiene	87-68-3	C(=C(Cl)Cl)(C(=C(Cl)Cl)Cl)Cl	Carcinogen
441	Hexachlorocyclope	77-47-4	C1(=C(C(C(=C1Cl)Cl)(Cl)Cl)Cl)Cl	Non-Carcinogen

	ntadiene			
442	Hexachlorophene	70-30-4	C1=C(C(=C(C(=C1Cl)Cl)CC2=C(C(=CC(=C2Cl)Cl)Cl)O)O)Cl	Non-Carcinogen
443	3-(Hexahydro-4,7-methanoindan-5-yl)-1,1-dimethylurea	2163-79-3	C/C=C/C=C/C=O	Carcinogen
444	Hexamethylenetetramine	100-97-0	C1N2CN3CN1CN(C2)C3	Non-Carcinogen
445	Hexamethylmelamine	531-18-0	C(N(CO)C1=NC(=NC(=N1)N(CO)CO)N(CO)CO)O	Carcinogen
446	Hexamethylphosphoramide	680-31-9	CN(C)P(=O)(N(C)C)N(C)C	Carcinogen
447	Hexanamide	628-02-4	CCCCCC(=O)N	Non-Carcinogen
448	Hexane	110-54-3	CCCCCC	Non-Carcinogen
449	1-O-Hexyl-2,3,5-trimethylhydroquinone	148081-72-5	CCCCCCOC1=C(C(=C(C(=C1)C)O)C)C	Non-Carcinogen
450	N-Hexylnitrosourea	18774-85-1	CCCCCCN(C(=O)N)N=O	Carcinogen
451	4-Hexylresorcinol	136-77-6	CCCCCCC1=C(C=C(C=C1)O)O	Non-Carcinogen
452	l-Histidine	645-35-2	C1=C(NC=N1)C[C@@H](C(=O)O)N	Non-Carcinogen
453	2-Hydrazino-4-(p-aminophenyl) thiazole	26049-71-8	C1=CC(=CC=C1C2=CSC(=N2)NN)N	Carcinogen
454	2-Hydrazino-4-(5-nitro-2-furyl)thiazole	26049-68-3	C1=C(OC(=C1)[N+](=O)[O-])C2=CSC(=N2)NN	Carcinogen
455	2-Hydrazino-4-(p-nitrophenyl) thiazole	26049-70-7	C1=CC(=CC=C1C2=CSC(=N2)NN)[N+](=O)[O-]	Carcinogen
456	2-Hydrazino-4-phenylthiazole	34176-52-8	C1=CC=C(C=C1)C2=CSC(=N2)NN	Non-Carcinogen
457	Hydrazobenzene	122-66-7	C1=CC=C(C=C1)NNC2=CC=CC=C2	Carcinogen
458	Hydrochlorothiazide	58-93-5	C1NC2=CC(=C(C=C2S(=O)(=O)N1)S(=O)(=O)N)Cl	Non-Carcinogen
459	Hydrocortisone	50-23-7	C[C@]12CCC(=O)C=C1CC[C@@H]3[C@@H]2[C@H](C[C@]4([C@H]3CC[C@@]4(C(=O)CO)O)C)O	Non-Carcinogen
460	Hydroquinone	123-31-9	C1=CC(=CC=C1O)O	Carcinogen
461	4-Acetamido-3-hydroxybiphenyl	13347-44-9	CC(=O)NC1=C(C=C(C=C1)C2=CC=CC=C2)O	Non-Carcinogen
462	N-Hydroxy-2-acet	53-95-2	CC(=O)N(C1=CC2=C(C=C1)C3=CC=CC=C3C2)O	Carcinogen

	ylaminofluorene			
463	3-Hydroxy-4-aminobiphenyl	4363-03-5	C1=CC=C(C=C1)C2=CC(=C(C=C2)N)O	Non-Carcinogen
464	1-Hydroxyanthraquinone	129-43-1	C1=CC=C2C(=C1)C(=O)C3=C(C2=O)C(=CC=C3)O	Carcinogen
465	1-(2-Hydroxyethyl)-3-[(5-nitrofurfurylidene)amino]-2-imidazolidinone	5036-03-3	C1CN(C(=O)N1CCO)/N=C/C2=CC=C(O2)[N+](=O)[O-]	Carcinogen
466	1-(2-Hydroxyethyl)-nitroso-3-ethylurea	96724-45-7	CCNC(=O)N(CCO)N=O	Carcinogen
467	1-(2-Hydroxyethyl)-1-nitrosourea	13743-07-2	C(CO)N(C(=O)N)N=O	Carcinogen
468	4-(2-Hydroxyethylamino)-2-(5-nitro-2-thienyl)quinazoline	33389-36-5	C1=CC=C2C(=C1)C(=NC(=N2)C3=CC=C(S3)[N+](=O)[O-])NCCO	Carcinogen
469	1-(3-Hydroxypropyl)-1-nitrosourea	71752-70-0	C(CN(C(=O)N)N=O)CO	Carcinogen
470	8-Hydroxyquinoline	148-24-3	C1=CC2=C(C(=C1)O)N=CC=C2	Non-Carcinogen
471	1'-Hydroxysafrole	5208-87-7	C=CC(C1=CC2=C(C=C1)OCO2)O	Carcinogen
472	Ibuprofen	15687-27-1	CC(C)CC1=CC=C(C=C1)C(C)C(=O)O	Non-Carcinogen
473	ICRF-159	21416-87-5	CC(CN1CC(=O)NC(=O)C1)N2CC(=O)NC(=O)C2	Carcinogen
474	3-((Imino((2,2,2-trifluoroethyl)amino)methyl)amino)1H-pyrazole-1-pentamide	84545-30-2	C1=CN(N=C1NC(=NCC(F)(F)F)N)CCCCC(=O)N	Carcinogen
475	3,3'-Iminobis-1-propanoldimethanesulfonate(ester)	3458-22-8	CS(=O)(=O)OCCCNCCCOS(=O)(=O)C	Non-Carcinogen
476	Iminodiacetic acid	32607-00-4	C(C(=O)O)NCC(=O)[O-]	Non-Carcinogen
477	Indole	120-72-9	CC1(C2=C(C=CC(=C2)C3=NNC(=O)CC3)NC1=O)C	Carcinogen
478	Indomethacin	53-86-1	CC1=C(C2=C(N1C(=O)C3=CC=C(C=C3)Cl)C=CC(=C2)OC)CC(=O)O	Carcinogen
479	Iodinated glycerol	5634-39-9	CC(C1OCC(O1)CO)I	Carcinogen
480	Ipazilide fumarate	115436-74-3	CCN(CC)CCCNC(=O)CN1C(=C(C=N1)C2=CC=CC=C2)C3=CC=CC=C3	Non-Carcinogen
481	IQ	76180-96-6	CN1C2=C(C3=C(C=C2)N=CC=C3)N=C1N	Carcinogen

482	Irsogladine maleate	84504-69-8	C1=CC(=C(C=C1Cl)C2=NC(=NC(=N2)N)N)Cl	Non-Carcinogen
483	Isatidine	15503-86-3	C/C=C\1/C[C@H]([C@@](C(=O)OCC2=CC[N+]3([C@H]2[C@@H](CC3)OC1=O)[O-])(CO)O)C	Carcinogen
484	Isobutene	115-11-7	CC(=C)C	Carcinogen
485	Isobutyl nitrite	542-56-3	CC(C)CON=O	Carcinogen
486	N-Isobutyl-N'-nitro-N-nitrosoguanidine	5461-85-8	CC(C)CN(/C(=N/[N+](=O)[O-])/N)N=O	Non-Carcinogen
487	Isobutyraldehyde	78-84-2	CC(C)C=O	Non-Carcinogen
488	Isomazole	86315-52-8	COC1=C(C=CC(=C1)S(=O)C)C2=NC3=C(N2)C=NC=C3	Carcinogen
489	Isoniazid	54-85-3	C1=CN=CC=C1C(=O)NN	Carcinogen
490	Isophorone	78-59-1	CC1=CC(=O)CC(C1)(C)C	Carcinogen
491	Isophosphamide	3778-73-2	C1CN(P(=O)(OC1)NCCCl)CCCl	Carcinogen
492	Isoprene	78-79-5	CC(=C)C=C	Carcinogen
493	Isopropanol	67-63-0	CC(C)O	Non-Carcinogen
494	Isopropyl-N-(3-chlorophenyl) carbamate	101-21-3	CC(C)OC(=O)NC1=CC(=CC=C1)Cl	Non-Carcinogen
495	Ivermectin	70288-86-7	CC[C@H](C)[C@@H]1[C@H](CC[C@@]2(O1)C[C@@H]3C[C@H](O2)C/C=C(/[C@H]([C@H](/C=C/C=C/4\CO[C@H]5[C@@]4([C@@H](C=C([C@H]5O)C)C(=O)O3)O)C)O[C@H]6C[C@@H]([C@H]([C@@H](O6)C)O[C@H]7C[C@@H]([C@H]([C@@H](O7)C)O)OC)OC)\C)C	Non-Carcinogen
496	Josamycin	16846-24-5	C[C@@H]1C/C=C/C=C/[C@@H]([C@@H](C[C@@H]([C@@H](C([C@@H](CC(=O)O1)OC(=O)C)OC)O[C@H]2[C@@H]([C@H]([C@@H]([C@H](O2)C)O[C@H]3C[C@@]([C@@H]([C@@H](O3)C)OC(=O)CC(C)C)(C)O)N(C)C)O)CC=O)C)O	Non-Carcinogen
497	Kaempferol	520-18-3	C1=CC(=CC=C1C2=C(C(=O)C3=C(C=C(C=C3O2)O)O)O)O	Non-Carcinogen
498	Kanechlor 400	12737-87-0	C1=C(C=C(C=C1Cl)Cl)C2=CC(=CC(=C2)Cl)Cl	Non-Carcinogen
499	Kepone	143-50-0	C1(=O)C2(C3(C4(C1(C5(C2(C3(C(C45Cl)(Cl)Cl)Cl)Cl)Cl)Cl)Cl)Cl)Cl	Carcinogen
500	Ketoprofen	22071-15-4	CC(C1=CC=CC(=C1)C(=O)C2=CC=CC=C2)C(=O)O	Non-Carcinogen
501	Lasiocarpine	303-34-4	C/C=C(/C)\C(=O)O[C@H]1CCN2[C@@H]1C(=CC2)COC(=O)[C@]([C@H](C)OC)(C(C)(C)O)O	Carcinogen
502	Levobunolol	27912-14-7	CC(C)(C)NC[C@@H](COC1=CC=CC2=C1CCCC2=O)O	Non-Carcinogen

503	d-Limonene	5989-27-5	CC1=CC[C@@H](CC1)C(=C)C	Carcinogen
504	Lithocholic acid	434-13-9	C[C@H](CCC(=O)O)[C@H]1CC[C@@H]2[C@@]1(CC[C@H]3[C@H]2CC[C@H]4[C@@]3(CC[C@H](C4)O)C)C	Non-Carcinogen
505	Lofexidine	21498-08-8	CC(C1=NCCN1)OC2=C(C=CC=C2Cl)Cl	Non-Carcinogen
506	Lonidamine	50264-69-2	C1=CC=C2C(=C1)C(=NN2CC3=C(C=C(C=C3)Cl)Cl)C(=O)O	Non-Carcinogen
507	Lornoxicam	70374-39-9	CN1C(=C(C2=C(S1(=O)=O)C=C(S2)Cl)O)C(=O)NC3=CC=CC=N3	Non-Carcinogen
508	Lovastatin	75330-75-5	CC[C@H](C)C(=O)O[C@H]1C[C@H](C=C2[C@H]1[C@H]([C@H](C=C2)C)CC[C@@H]3C[C@H](CC(=O)O3)O)C	Non-Carcinogen
509	Loxtidine	76956-02-0	CN1C(=NC(=N1)CO)NCCCOC2=CC=CC(=C2)CN3CCCCC3	Carcinogen
510	Lupitidine	72716-75-7	CC1=NC=C(C=C1)CC2=CN=C(NC2=O)NCCSCC3=CC=C(O3)CN(C)C	Carcinogen
511	Malaoxon	1634-78-2	CCOC(=O)CC(C(=O)OCC)SP(=O)(OC)OC	Non-Carcinogen
512	Malathion	121-75-5	CCOC(=O)CC(C(=O)OCC)SP(=S)(OC)OC	Non-Carcinogen
513	Maleic hydrazide	123-33-1	C1=CC(=O)NNC1=O	Non-Carcinogen
514	Malonaldehyde	24382-04-5	C(=C/[O-])\C=O	Carcinogen
515	Manganese ethylenebisthiocarbamate	12427-38-2	C(CNC(=S)[S-])NC(=S)[S-]	Carcinogen
516	Manidipine	89226-75-5	CC1=C(C(C(=C(N1)C)C(=O)OCCN2CCN(CC2)C(C3=CC=CC=C3)C4=CC=CC=C4)C5=CC(=CC=C5)[N+](=O)[O-])C(=O)OC	Non-Carcinogen
517	d-Mannitol	69-65-8	C([C@H]([C@H]([C@@H]([C@@H](CO)O)O)O)O)O	Non-Carcinogen
518	Mannitol nitrogen mustard	576-68-1	C(CCl)NC[C@H]([C@H]([C@@H]([C@@H](CNCCCl)O)O)O)O	Non-Carcinogen
519	MeA-alpha-C acetate	117831-29-5	CC1=C(N=C2C(=C1)C3=CC=CC=C3N2)[NH3+]	Carcinogen
520	MeIQx	77500-04-0	CC1=CN=C2C=CC3=C(C2=N1)N=C(N3C)N	Carcinogen
521	Melamine	108-78-1	C1(=NC(=NC(=N1)N)N)N	Carcinogen
522	Meloxicam	71125-38-7	CC1=CN=C(S1)NC(=O)C2=C(C3=CC=CC=C3S(=O)(=O)N2C)O	Non-Carcinogen
523	Melphalan	148-82-3	C1=CC(=CC=C1C[C@@H](C(=O)O)N)N(CCCl)CCCl	Carcinogen
524	dl-Menthol	15356-70-4	CC1CCC(C(C1)O)C(C)C	Non-Carcinogen
525	MER-25	67-98-1	CCN(CC)CCOC1=CC=C(C=C1)C(CC2=CC=C(C=C2)OC)(C3=CC=CC=C3)O	Non-Carcinogen
526	2-Mercaptobenzoth	149-30-4	C1=CC=C2C(=C1)NC(=S)S2	Carcinogen

	iazole			
527	6-Mercaptopurine	50-44-2	C1=NC2=C(N1)C(=S)N=CN2	Non-Carcinogen
528	Metepa	57-39-6	CC1CN1P(=O)(N2CC2C)N3CC3C	Carcinogen
529	Methacrylonitrile	126-98-7	CC(=C)C#N	Non-Carcinogen
530	Methafurylene	531-06-6	CN(C)CCN(CC1=CC=CO1)C2=CC=CC=N2	Non-Carcinogen
531	Methaphenilene	493-78-7	CN(C)CCN(CC1=CC=CS1)C2=CC=CC=C2	Non-Carcinogen
532	Methapyrilene	135-23-9	CN(C)CCN(CC1=CC=CS1)C2=CC=CC=N2	Carcinogen
533	Methimazole	60-56-0	CN1C=CNC1=S	Carcinogen
534	dl-Methionine	59-51-8	CSCCC(C(=O)O)N	Non-Carcinogen
535	Methotrexate	59-05-2	CN(CC1=CN=C2C(=N1)C(=NC(=N2)N)N)C3=CC=C(C=C3)C(=O)N[C@@H](CCC(=O)O)C(=O)O	Non-Carcinogen
536	2-Methoxy-3-aminodibenzofuran	5834-17-3	COC1=C(C=C2C(=C1)C3=CC=CC=C3O2)N	Carcinogen
537	3-Methoxycatechol	934-00-9	COC1=CC=CC(=C1O)O	Carcinogen
538	Methoxychlor	72-43-5	COC1=CC=C(C=C1)C(C2=CC=C(C=C2)OC)C(Cl)(Cl)Cl	Non-Carcinogen
539	4-Methoxyphenol	150-76-5	COC1=CC=C(C=C1)O	Carcinogen
540	8-Methoxypsoralen	298-81-7	COC1=C2C(=CC3=C1OC=C3)C=CC(=O)O2	Carcinogen
541	Z-Methyl-O,N,N-azoxyethane	57497-34-4	CCN=[N+](C)[O-]	Carcinogen
542	Methyl tert-butyl ether	1634-04-4	CC(C)(C)OC	Carcinogen
543	2-Methyl-4-chlorophenoxyacetic acid	94-74-6	CC1=C(C=CC(=C1)Cl)OCC(=O)O	Non-Carcinogen
544	Methyl clofenapate	21340-68-1	CC(C)(C(=O)OC)OC1=CC=C(C=C1)C2=CC=C(C=C2)Cl	Carcinogen
545	3'-Methyl-4-dimethylaminoazobenzene	55-80-1	CC1=CC(=CC=C1)N=NC2=CC=C(C=C2)N(C)C	Carcinogen
546	N-Methyl-N,4-dinitrosoaniline	99-80-9	CN(C1=CC=C(C=C1)N=O)N=O	Carcinogen
547	methyl (9Z,11E)-13-hydroperoxyoctadeca-9,11-dienoate	NOCAS	CCCCCC(/C=C/C=C\CCCCCCCC(=O)OC)OO	Non-Carcinogen
548	Methyl linoleate	112-63-0	CCCCC/C=C\C/C=C\CCCCCCCC(=O)OC	Non-Carcinogen
549	Methyl methacrylate	80-62-6	CC(=C)C(=O)OC	Non-Carcinogen
550	2-Methyl-1-nitroanthraquinone	129-15-7	CC1=C(C2=C(C=C1)C(=O)C3=CC=CC=C3C2=O)[N+](=O)[O-]	Carcinogen
551	4-Methyl-1-[(5-nitrofurfurylidene)ami	21638-36-8	CC1CN(C(=O)N1)/N=C/C2=CC=C(O2)[N+](=O)[O-]	Carcinogen

	no]-2-imidazolidinone			
552	4-(4-N-Methyl-N-nitrosaminostyryl)quinoline	16699-10-8	CN(C1=CC=C(C=C1)/C=C/C2=CC=NC3=CC=CC=C23)N=O	Carcinogen
553	N-Methyl-N-nitrosobenzamide	63412-06-6	CN(C(=O)C1=CC=CC=C1)N=O	Carcinogen
554	N-(N-Methyl-N-nitrosocarbamoyl)-l-ornithine	63642-17-1	CN(C(=O)NCCC[C@@H](C(=O)O)N)N=O	Carcinogen
555	R(-)-2-Methyl-N-nitrosopiperidine	14026-03-0	C[C@@H]1CCCCN1N=O	Carcinogen
556	S(+)-2-Methyl-N-nitrosopiperidine	36702-44-0	C[C@H]1CCCCN1N=O	Carcinogen
557	Methyl parathion	298-00-0	COP(=S)(OC)OC1=CC=C(C=C1)[N+](=O)[O-]	Non-Carcinogen
558	N-Methyl-2-pyrrolidone	872-50-4	CN1CCCC1=O	Non-Carcinogen
559	alpha-Methylbenzyl alcohol	98-85-1	CC(C1=CC=CC=C1)O	Carcinogen
560	4-Methylcatechol	452-86-8	CC1=CC(=C(C=C1)O)O	Carcinogen
561	3-Methylcholanthrene	56-49-5	CC1=C2CCC3=C2C(=CC4=C3C=CC5=CC=CC=C54)C=C1	Carcinogen
562	alpha-Methyldopa sesquihydrate	41372-08-1	C[C@](CC1=CC(=C(C=C1)O)O)(C(=O)O)N	Non-Carcinogen
563	N-Methyldopamine,O,O'-diisobutyroylester	75011-65-3	CC(C)C(=O)OC1=C(C=C(C=C1)CCNC)OC(=O)C(C)C	Non-Carcinogen
564	4,4'-Methylene-bis(2-methylaniline)	838-88-0	CC1=C(C=CC(=C1)CC2=CC(=C(C=C2)N)C)N	Carcinogen
565	4,4'-Methylenebis(N,N-dimethyl) benzenamine	101-61-1	CN(C)C1=CC=C(C=C1)CC2=CC=C(C=C2)N(C)C	Carcinogen
566	2,2'-Methylenebis(4-methyl-6-tert-butylphenol)	119-47-1	CC1=CC(=C(C(=C1)C(C)(C)C)O)CC2=C(C(=CC(=C2)C)C(C)(C)C)O	Non-Carcinogen
567	4,4'-Methylenedianiline	13552-44-8	C1=CC(=CC=C1CC2=CC=C(C=C2)N)N	Carcinogen
568	Methylethylketoxime	96-29-7	CC/C(=N/O)/C	Carcinogen
569	Methylguanidine	471-29-4	COC1=C(C=C(C=C1)CC=C)OC	Carcinogen
570	7-Methylguanine	578-76-7	CN1C=NC2=C1C(=O)N=C(N2)N	Non-Carcinogen
571	Methylhydroquino	95-71-6	CC1=C(C=CC(=C1)O)O	Non-Carcinogen

	ne			
572	2-Methylimidazole	693-98-1	CC1=NC=CN1	Carcinogen
573	4-(Methylnitrosamino)-1-(3-pyridyl)-1-butanol	76014-81-8	CN(CCCC(C1=CN=CC=C1)O)N=O	Carcinogen
574	4-(Methylnitrosamino)-1-(3-pyridyl)-1-(butanone)	64091-91-4	CN(CCCC(=O)C1=CN=CC=C1)N=O	Carcinogen
575	N-Methylolacrylamide	924-42-5	C=CC(=O)NCO	Non-Carcinogen
576	Methylphenidate	298-59-9	COC(=O)C(C1CCCCN1)C2=CC=CC=C2	Non-Carcinogen
577	6-Methylquinoline	91-62-3	CC1=CC2=C(C=C1)N=CC=C2	Non-Carcinogen
578	8-Methylquinoline	611-32-5	CC1=CC=CC2=C1N=CC=C2	Non-Carcinogen
579	Metiapine	5800-19-1	CC1=CC2=C(C=C1)SC3=CC=CC=C3N=C2N4CCN(CC4)C	Non-Carcinogen
580	Metronidazole	443-48-1	CC1=NC=C(N1CCO)[N+](=O)[O-]	Carcinogen
581	Mexacarbate	315-18-4	CC1=CC(=CC(=C1N(C)C)C)OC(=O)NC	Non-Carcinogen
582	Michler's ketone	90-94-8	CN(C)C1=CC=C(C=C1)C(=O)C2=CC=C(C=C2)N(C)C	Carcinogen
583	Mirex	2385-85-5	C12(C3(C4(C5(C3(C(C1(C5(C2(C4(Cl)Cl)Cl)Cl)Cl)(Cl)Cl)Cl)Cl)Cl)Cl)Cl	Carcinogen
584	Photomirex	39801-14-4	C12C3(C4(C5(C3(C(C1(C5(C2(C4(Cl)Cl)Cl)Cl)Cl)(Cl)Cl)Cl)Cl)Cl)Cl	Carcinogen
585	Misoprostol	59122-46-2	CCCCC(C)(C/C=C/[C@H]1[C@@H](CC(=O)[C@@H]1CCCCCCC(=O)OC)O)O	Non-Carcinogen
586	Mitomycin-C	50-07-7	CC1=C(C(=O)C2=C(C1=O)N3C[C@H]4[C@@H]([C@@]3([C@@H]2COC(=O)N)OC)N4)N	Carcinogen
587	Monocrotaline	315-22-0	C[C@H]1C(=O)O[C@@H]2CCN3[C@@H]2C(=CC3)COC(=O)[C@]([C@]1(C)O)(C)O	Carcinogen
588	Mononitrosocaffeidine	145438-96-6	CNC(=O)C1=C(N=CN1C)N(C)N=O	Carcinogen
589	Monosodium aspartate	3792-50-5	C([C@@H](C(=O)[O-])N)C(=O)O	Non-Carcinogen
590	l-Monosodium glutamate	142-47-2	C(CC(=O)O)[C@@H](C(=O)[O-])N	Non-Carcinogen
591	Monosodium succinate	2922-54-5	C(CC(=O)[O-])C(=O)O	Non-Carcinogen
592	4-Morpholino-2-(5-nitro-2-thienyl)quinazoline	58139-48-3	C1COCCN1C2=NC(=NC3=CC=CC=C32)C4=CC=C(S4)[N+](=O)[O-]	Carcinogen
593	l-5-Morpholinomethyl-3-[(5-nitrofurfu	3031-51-4	C1COCCN1C[C@H]2CN(C(=O)O2)/N=C/C3=CC=C(O3)[N+](=O)[O-]	Carcinogen

	rylidene)amino]-2-oxazolidinone			
594	Myleran	55-98-1	CS(=O)(=O)OCCCCOS(=O)(=O)C	Non-Carcinogen
595	Nafenopin	3771-19-5	CC(C)(C(=O)O)OC1=CC=C(C=C1)C2CCCC3=CC=CC=C23	Carcinogen
596	Nalidixic acid	389-08-2	CCN1C=C(C(=O)C2=C1N=C(C=C2)C)C(=O)O	Carcinogen
597	Naphthalene	91-20-3	C1=CC=C2C=CC=CC2=C1	Carcinogen
598	1,5-Naphthalenediamine	2243-62-1	C1=CC2=C(C=CC=C2N)C(=C1)N	Carcinogen
599	N-(1-Naphthyl)ethylenediamine	1465-25-4	C1=CC=C2C(=C1)C=CC=C2NCCN	Non-Carcinogen
600	2-Naphthylamine	91-59-8	C1=CC=C2C=C(C=CC2=C1)N	Carcinogen
601	Nefiracetam	77191-36-7	CC1=C(C(=CC=C1)C)NC(=O)CN2CCCC2=O	Non-Carcinogen
602	Nicotine	54-11-5	CN1CCC[C@H]1C2=CN=CC=C2	Non-Carcinogen
603	Nithiazide	139-94-6	CCNC(=O)NC1=NC=C(S1)[N+](=O)[O-]	Carcinogen
604	Nitrilotriacetic acid	139-13-9	C(C(=O)O)N(CC(=O)O)CC(=O)O	Carcinogen
605	Nitrilotriacetic acid	18662-53-8	C(C(=O)[O-])N(CC(=O)[O-])CC(=O)[O-]	Carcinogen
606	3-Nitro-p-acetophenetide	1777-84-0	CCOC1=C(C=C(C=C1)NC(=O)C)[N+](=O)[O-]	Non-Carcinogen
607	5-Nitro-o-anisidine	99-59-2	COC1=C(C=C(C=C1)[N+](=O)[O-])N	Carcinogen
608	5-Nitro-2-furaldehyde semicarbazone	59-87-0	C1=C(OC(=C1)[N+](=O)[O-])/C=N/NC(=O)N	Carcinogen
609	5-Nitro-2-furamidoxime	772-43-0	C1=C(OC(=C1)[N+](=O)[O-])/C(=N/O)/N	Non-Carcinogen
610	5-Nitro-2-furanmethanediol diacetate	92-55-7	CC(=O)OC(C1=CC=C(O1)[N+](=O)[O-])OC(=O)C	Non-Carcinogen
611	3-(5-Nitro-2-furyl)-imidazo(1,2-alpha) pyridine	75198-31-1	C1=CC2=NC=C(N2C=C1)C3=CC=C(O3)[N+](=O)[O-]	Carcinogen
612	5-(5-Nitro-2-furyl)-1,3,4-oxadiazole-2-ol	2122-86-3	C1=C(OC(=C1)[N+](=O)[O-])C2=NNC(=O)O2	Carcinogen
613	N-{[3-(5-Nitro-2-furyl)-1,2,4-oxadiazole-5-yl]-methyl}acetamide	36133-88-7	CC(=O)NCC1=NC(=NO1)C2=CC=C(O2)[N+](=O)[O-]	Carcinogen
614	N-[5-(5-Nitro-2-furyl)-1,3,4-thiadiazol-2-yl]acetamide	2578-75-8	CC(=O)NC1=NN=C(S1)C2=CC=C(O2)[N+](=O)[O-]	Carcinogen
615	4-(5-Nitro-2-furyl)thiazole	53757-28-1	C1=C(OC(=C1)[N+](=O)[O-])C2=CSC=N2	Carcinogen
616	N-[4-(5-Nitro-2-fur	531-82-8	CC(=O)NC1=NC(=CS1)C2=CC=C(O2)[N+](=O)[	Carcinogen

	yl)-2-thiazolyl]acetamide		O-]	
617	N-[4-(5-Nitro-2-furyl)-2-thiazolyl]formamide	24554-26-5	C1=C(OC(=C1)[N+](=O)[O-])C2=CSC(=N2)NC=O	Carcinogen
618	N,N'-[6-(5-Nitro-2-furyl)-S-triazine-2,4-diyl]bisacetamide	51325-35-0	CC(=O)NC1=NC(=NC(=N1)C2=CC=C(O2)[N+](=O)[O-])NC(=O)C	Carcinogen
619	3-Nitro-3-hexene	4812-22-0	CC/C=C(/CC)\[N+](=O)[O-]	Carcinogen
620	2-Nitro-p-phenylenediamine	5307-14-2	C1=CC(=C(C=C1N)[N+](=O)[O-])N	Non-Carcinogen
621	4-Nitro-o-phenylenediamine	99-56-9	C1=CC(=C(C=C1[N+](=O)[O-])N)N	Non-Carcinogen
622	5-Nitro-o-toluidine	99-55-8	CC1=C(C=C(C=C1)[N+](=O)[O-])N	Non-Carcinogen
623	5-Nitroacenaphthene	602-87-9	C1CC2=CC=CC3=C(C=CC1=C23)[N+](=O)[O-]	Carcinogen
624	p-Nitroaniline	100-01-6	C1=CC(=CC=C1N)[N+](=O)[O-]	Non-Carcinogen
625	o-Nitroanisole	91-23-6	COC1=CC=CC=C1[N+](=O)[O-]	Carcinogen
626	4-Nitroanthranilic acid	619-17-0	C1=CC(=C(C=C1[N+](=O)[O-])N)C(=O)O	Non-Carcinogen
627	Nitrobenzene	98-95-3	C1=CC=C(C=C1)[N+](=O)[O-]	Carcinogen
628	6-Nitrobenzimidazole	94-52-0	C1=CC2=C(C=C1[N+](=O)[O-])NC=N2	Non-Carcinogen
629	p-Nitrobenzoic acid	62-23-7	C1=CC(=CC=C1C(=O)O)[N+](=O)[O-]	Carcinogen
630	1-Nitrobutane	627-05-4	CCCC[N+](=O)[O-]	Non-Carcinogen
631	2-Nitrobutane	600-24-8	CCC(C)[N+](=O)[O-]	Carcinogen
632	Nitrofen	1836-75-5	C1=CC(=CC=C1[N+](=O)[O-])OC2=C(C=C(C=C2)Cl)Cl	Carcinogen
633	2-Nitrofluorene	607-57-8	C1C2=CC=CC=C2C3=C1C=C(C=C3)[N+](=O)[O-]	Carcinogen
634	1-[(5-Nitrofurfurylidene)amino]hydantoin	67-20-9	C1C(=O)NC(=O)N1/N=C/C2=CC=C(O2)[N+](=O)[O-]	Carcinogen
635	1-[(5-Nitrofurfurylidene)amino]-2-imidazolidinone	555-84-0	C1CN(C(=O)N1)/N=C/C2=CC=C(O2)[N+](=O)[O-]	Carcinogen
636	Nitrogen mustard	51-75-2	CN(CCCl)CCCl	Carcinogen
637	Nitrogen mustard N-oxide	126-85-2	C[N+](CCCl)(CCCl)[O-]	Carcinogen
638	1-Nitronaphthalene	86-57-7	C1=CC=C2C(=C1)C=CC=C2[N+](=O)[O-]	Non-Carcinogen

639	3-Nitropentane	551-88-2	CCC(CC)[N+](=O)[O-]	Carcinogen
640	1-Nitropropane	108-03-2	CCC[N+](=O)[O-]	Non-Carcinogen
641	2-Nitropropane	79-46-9	CC(C)[N+](=O)[O-]	Non-Carcinogen
642	3-Nitropropionic acid	504-88-1	C(C[N+](=O)[O-])C(=O)O	Non-Carcinogen
643	1-Nitropyrene	5522-43-0	C1=CC2=C3C(=C1)C=CC4=C(C=CC(=C43)C=C2)[N+](=O)[O-]	Carcinogen
644	6-Nitroquinoline	613-50-3	C1=CC2=C(C=CC(=C2)[N+](=O)[O-])N=C1	Non-Carcinogen
645	8-Nitroquinoline	607-35-2	C1=CC2=C(C(=C1)[N+](=O)[O-])N=CC=C2	Carcinogen
646	Nitroso-Baygon	38777-13-8	CC(C)OC1=CC=CC=C1OC(=O)N(C)N=O	Carcinogen
647	N-Nitroso-bis-(4,4,4-trifluoro-N-butyl)amine	83335-32-4	C(CC(F)(F)F)CN(CCCC(F)(F)F)N=O	Carcinogen
648	1-Nitroso-5,6-dihydrothymine	62641-67-2	CC1CN(C(=O)NC1=O)N=O	Non-Carcinogen
649	1-Nitroso-5,6-dihydrouracil	16813-36-8	C1CN(C(=O)NC1=O)N=O	Carcinogen
650	N-Nitroso-2,3-dihydroxypropyl-2-hydroxypropylamine	89911-79-5	CC(CN(CC(CO)O)N=O)O	Carcinogen
651	Nitroso-2,3-dihydroxypropyl-2-oxopropylamine	92177-50-9	CC(=O)CN(CC(CO)O)N=O	Carcinogen
652	N-Nitroso-2,3-dihydroxypropylethanolamine	89911-78-4	C(CO)N(CC(CO)O)N=O	Carcinogen
653	1-Nitroso-3,5-dimethyl-4-benzoylpiperazine	61034-40-0	CC1CN(CC(N1C(=O)C2=CC=CC=C2)C)N=O	Carcinogen
654	1-Nitroso-1-hydroxyethyl-3-chloroethylurea	96806-34-7	C(CCl)NC(=O)N(CCO)N=O	Carcinogen
655	N-Nitroso-2-hydroxymorpholine	67587-52-4	C1COC(CN1N=O)O	Non-Carcinogen
656	1-Nitroso-1-(2-hydroxypropyl)-3-chloroethylurea	96806-35-8	CC(CN(C(=O)NCCCl)N=O)O	Carcinogen
657	N-Nitroso-(2-hydroxypropyl)-(2-hydroxyethyl)amine	75896-33-2	CC(CN(CCO)N=O)O	Carcinogen
658	N-Nitroso-3-hydroxypyrrolidine	56222-35-6	C1CN(CC1O)N=O	Carcinogen

659	N-Nitroso-N-isobutylurea	760-60-1	CC(C)CN(C(=O)N)N=O	Carcinogen
660	N-Nitroso-N-methyl-N-dodecylamine	55090-44-3	CCCCCCCCCCCCN(C)N=O	Carcinogen
661	N-Nitroso-N-methyl-4-fluoroaniline	937-25-7	CN(C1=CC=C(C=C1)F)N=O	Carcinogen
662	N-Nitroso-N-methyl-4-nitroaniline	943-41-9	CN(C1=CC=C(C=C1)[N+](=O)[O-])N=O	Non-Carcinogen
663	Nitroso-N-methyl-N-(2-phenyl)ethylamine	13256-11-6	CN(CCC1=CC=CC=C1)N=O	Carcinogen
664	N-Nitroso-N-methyl-N-tetradecylamine	75881-20-8	CCCCCCCCCCCCCCN(C)N=O	Carcinogen
665	N-Nitroso-N-methyldecylamine	75881-22-0	CCCCCCCCCCN(C)N=O	Carcinogen
666	3-Nitroso-2-oxazolidinone	38347-74-9	C1COC(=O)N1N=O	Carcinogen
667	Nitroso-2-oxopropylethanolamine	92177-49-6	CC(=O)CN(CCO)N=O	Carcinogen
668	di(N-Nitroso)-perhydropyrimidine	15973-99-6	C1CN(CN(C1)N=O)N=O	Carcinogen
669	Nitroso-1,2,3,6-tetrahydropyridine	55556-92-8	C1CN(CC=C1)N=O	Carcinogen
670	N-Nitroso(2,2,2-trifluoroethyl) ethylamine	82018-90-4	CCN(CC(F)(F)F)N=O	Carcinogen
671	N-Nitroso-2,2,4-trimethyl-1,2-dihydroquinoline polymer	29929-77-9	CC1=CC(N(C2=CC=CC=C12)N=O)(C)C	Carcinogen
672	1-Nitroso-3,4,5-trimethylpiperazine	75881-18-4	CC1CN(CC(N1C)C)N=O	Carcinogen
673	N-Nitrosoallyl-2,3-dihydroxypropylamine	88208-16-6	C=CCN(CC(CO)O)N=O	Carcinogen
674	N-Nitrosoallyl-2-hydroxypropylamine	91308-70-2	CC(CN(CC=C)N=O)O	Carcinogen
675	N-Nitrosoallyl-2-oxopropylamine	91308-71-3	CC(=O)CN(CC=C)N=O	Carcinogen
676	N-Nitrosoallylethanolamine	91308-69-9	C=CCN(CCO)N=O	Carcinogen

677	Nitrosoamylurethane	64005-62-5	CCCCCN(C(=O)OCC)N=O	Carcinogen
678	Nitrosoanabasine	1133-64-8	C1CCN(C(C1)C2=CN=CC=C2)N=O	Carcinogen
679	N-Nitrosobenzthiazuron	51542-33-7	CN(C(=O)NC1=NC2=CC=CC=C2S1)N=O	Carcinogen
680	N-Nitrosobis(2-hydroxypropyl)amine	53609-64-6	CC(CN(CC(C)O)N=O)O	Carcinogen
681	N-Nitrosobis(2-oxopropyl)amine	60599-38-4	CC(=O)CN(CC(=O)C)N=O	Carcinogen
682	N-Nitrosobis(2,2,2-trifluoroethyl) amine	625-89-8	C(C(F)(F)F)N(CC(F)(F)F)N=O	Non-Carcinogen
683	Nitrosochlordiazepoxide	51715-17-4	CC1=C(N=CN1)CSCCN=C(NC#N)N(C)N=O	Non-Carcinogen
684	Nitrosodibutylamine	924-16-3	CCCCN(CCCC)N=O	Carcinogen
685	N-Nitrosodiethanolamine	1116-54-7	C(CO)N(CCO)N=O	Carcinogen
686	N-Nitrosodiethylamine	55-18-5	CCN(CC)N=O	Carcinogen
687	N-Nitrosodiphenylamine	86-30-6	C1=CC=C(C=C1)N(C2=CC=CC=C2)N=O	Carcinogen
688	p-Nitrosodiphenylamine	156-10-5	C1=CC=C(C=C1)NC2=CC=C(C=C2)N=O	Carcinogen
689	N-Nitrosodipropylamine	621-64-7	CCCN(CCC)N=O	Carcinogen
690	N-Nitrosodithiazine	114282-83-6	C1N(CSCS1)N=O	Non-Carcinogen
691	Nitrosododecamethyleneimine	40580-89-0	C1CCCCCCN(CCCCC1)N=O	Carcinogen
692	N-Nitrosoephedrine	17608-59-2	CC(C(C1=CC=CC=C1)O)N(C)N=O	Carcinogen
693	Nitrosoethylmethylamine	10595-95-6	CCN(C)N=O	Carcinogen
694	Nitrosoethylurethane	614-95-9	CCN(C(=O)OCC)N=O	Carcinogen
695	N-Nitrosoguvacoline	55557-02-3	COC(=O)C1=CCCN(C1)N=O	Non-Carcinogen
696	Nitrosoheptamethyleneimine	20917-49-1	C1CCCN(CCC1)N=O	Carcinogen
697	1-Nitrosohydantoin	42579-28-2	C1C(=O)NC(=O)N1N=O	Carcinogen
698	Nitrosohydroxypro	30310-80-6	C1[C@H](CN([C@@H]1C(=O)O)N=O)O	Non-Carcinogen

	line			
699	Nitrosoiminodiacetic acid	25081-31-6	C(C(=O)O)N(CC(=O)O)N=O	Non-Carcinogen
700	Nitrosomethyl-3-carboxypropylamine	61445-55-4	CN(CCCC(=O)O)N=O	Carcinogen
701	N-Nitrosomethyl-2,3-dihydroxypropylamine	86451-37-8	CN(CC(CO)O)N=O	Carcinogen
702	N-Nitrosomethyl-(2-hydroxyethyl) amine	26921-68-6	CN(CCO)N=O	Carcinogen
703	N-Nitrosomethyl-(3-hydroxypropyl) amine	70415-59-7	CN(CCCO)N=O	Carcinogen
704	N-Nitrosomethyl-2-hydroxypropylamine	75411-83-5	CC(CN(C)N=O)O	Carcinogen
705	N-Nitrosomethyl(2-oxopropyl)amine	55984-51-5	CC(=O)CN(C)N=O	Carcinogen
706	N-Nitrosomethyl-(2-tosyloxyethyl) amine	NOCAS	CC1=CC=C(C=C1)S(=O)(=O)OCCN(C)N=O	Carcinogen
707	2-Nitrosomethylaminopyridine	16219-98-0	CN(C1=CC=CC=N1)N=O	Carcinogen
708	3-Nitrosomethylaminopyridine	69658-91-9	CN(C1=CN=CC=C1)N=O	Non-Carcinogen
709	4-Nitrosomethylaminopyridine	16219-99-1	CN(C1=CC=NC=C1)N=O	Non-Carcinogen
710	Nitrosomethylaniline	614-00-6	CN(C1=CC=CC=C1)N=O	Carcinogen
711	Nitrosomethylundecylamine	68107-26-6	CCCCCCCCCCCN(C)N=O	Carcinogen
712	N-Nitrosomorpholine	59-89-2	C1COCCN1N=O	Carcinogen
713	N'-Nitrosonornicotine	53759-22-1	C1CC(N(C1)N=O)C2=CN=CC=C2	Carcinogen
714	N'-Nitrosonornicotine-1-N-oxide	78246-24-9	C1CC(N(C1)N=O)C2=C[N+](=CC=C2)[O-]	Carcinogen
715	Nitrosopipecolic acid	4515-18-8	C1CCN(C(C1)C(=O)O)N=O	Non-Carcinogen
716	N-Nitrosopiperazine	5632-47-3	C1CN(CCN1)N=O	Carcinogen

717	N-Nitrosopiperidine	100-75-4	C1CCN(CC1)N=O	Carcinogen
718	Nitrosoproline	7519-36-0	C1C[C@H](N(C1)N=O)C(=O)O	Non-Carcinogen
719	N-Nitrosopyrrolidine	930-55-2	C1CCN(C1)N=O	Carcinogen
720	N-Nitrosothialdine	81795-07-5	CC1N(C(SC(S1)C)C)N=O	Carcinogen
721	N-Nitrosothiomorpholine	26541-51-5	C1CSCCN1N=O	Carcinogen
722	o-Nitrosotoluene	611-23-4	CC1=CC=CC=C1N=O	Carcinogen
723	o-Nitrotoluene	88-72-2	CC1=CC=CC=C1[N+](=O)[O-]	Carcinogen
724	p-Nitrotoluene	99-99-0	CC1=CC=C(C=C1)[N+](=O)[O-]	Carcinogen
725	Norharman	244-63-3	C1=CC=C2C(=C1)C3=C(N2)C=NC=C3	Non-Carcinogen
726	Norlestrin	51-98-9	CC(=O)O[C@]1(CC[C@@H]2[C@@]1(CC[C@H]3[C@H]2CCC4=CC(=O)CC[C@H]34)C)C#C	Carcinogen
727	Novadelox	94-36-0	C1=CC=C(C=C1)C(=O)OOC(=O)C2=CC=CC=C2	Non-Carcinogen
728	Ochratoxin A	303-47-9	C[C@@H]1CC2=C(C=C(C(=C2C(=O)O1)O)C(=O)N[C@@H](CC3=CC=CC=C3)C(=O)O)Cl	Carcinogen
729	Octachlorostyrene	29082-74-4	C1(=C(C(=C(C(=C1Cl)Cl)Cl)Cl)Cl)C(=C(Cl)Cl)Cl	Non-Carcinogen
730	Oleate	143-19-1	CCCCCCCC/C=C\CCCCCCCC(=O)[O-]	Non-Carcinogen
731	Oltipraz	64224-21-1	CC1=C(SSC1=S)C2=NC=CN=C2	Non-Carcinogen
732	Omeprazole	73590-58-6	CC1=CN=C(C(=C1OC)C)CS(=O)C2=NC3=C(N2)C=C(C=C3)OC	Carcinogen
733	C.I. acid orange 3	6373-74-6	C1=CC=C(C=C1)NC2=C(C=C(C=C2)NC3=C(C=C(C=C3)[N+](=O)[O-])[N+](=O)[O-])S(=O)(=O)[O-]	Carcinogen
734	C.I. acid orange 10	1936-15-8	C1=CC=C(C=C1)N/N=C/2\C(=O)C=CC3=CC(=CC(=C32)S(=O)(=O)[O-])S(=O)(=O)[O-]	Non-Carcinogen
735	Orotic acid	154-85-8	C1=C(NC(=O)NC1=O)C(=O)[O-]	Non-Carcinogen
736	gamma-Oryzanol	11042-64-1	C[C@H](CCC=C(C)C)[C@H]1CC[C@@]2([C@@]1(CC[C@]34[C@H]2CC[C@@H]5[C@]3(C4)CC[C@@H](C5(C)C)OC(=O)/C=C/C6=CC(=C(C=C6)O)OC)C)C	Non-Carcinogen
737	Osutidine	140695-21-2	CNCC1=CC=C(O1)CSCCNC(=NCC(C2=CC=C(C=C2)O)O)NS(=O)(=O)C	Non-Carcinogen
738	Oxamyl	23135-22-0	CNC(=O)O/N=C(/C(=O)N(C)C)\SC	Non-Carcinogen
739	Oxazepam	604-75-1	C1=CC=C(C=C1)C2=NC(C(=O)NC3=C2C=C(C=C3)Cl)O	Non-Carcinogen
740	N-(9-Oxo-2-fluorenyl)acetamide	3096-50-2	CC(=O)NC1=CC2=C(C=C1)C3=CC=CC=C3C2=O	Carcinogen
741	Oxolinic acid	14698-29-4	CCN1C=C(C(=O)C2=CC3=C(C=C21)OCO3)C(=O)O	Carcinogen
742	1-(2-Oxopropyl)nit	110559-85-8	CC(=O)CN(C(=O)NCCCl)N=O	Non-Carcinogen

	roso-3-(2-chloroethyl)urea			
743	2-Oxopropylnitrosourea	89837-93-4	CC(=O)CN(C(=O)N)N=O	Non-Carcinogen
744	1'-Oxosafrole	30418-53-2	C=CC(=O)C1=CC2=C(C=C1)OCO2	Non-Carcinogen
745	Oxprenolol	6452-73-9	CC(C)NCC(COC1=CC=CC=C1OCC=C)O	Non-Carcinogen
746	4,4'-Oxydianiline	101-80-4	C1=CC(=CC=C1N)OC2=CC=C(C=C2)N	Carcinogen
747	N-Oxydiethylene thiocarbamyl-N-oxydiethylene sulfenamide	13752-51-7	C1COCCN1C(=S)SN2CCOCC2	Carcinogen
748	Oxymetholone	434-07-1	C[C@]12CC[C@H]3[C@H]([C@@H]1CC[C@]2(C)O)CC[C@@H]4[C@@]3(C/C(=C/O)/C(=O)C4)C	Carcinogen
749	Oxytetracycline	2058-46-0	C[C@@]1([C@H]2[C@@H]([C@H]3[C@@H](C(=O)C(=C([C@]3(C(=O)C2=C(C4=C1C=CC=C4O)O)O)O)C(=O)N)N(C)C)O)O	Non-Carcinogen
750	Palonidipine	96515-74-1	CC1=C(C(C(=C(N1)C)C(=O)OCC(C)(C)CN(C)CC2=CC=CC=C2)C3=C(C=CC(=C3)[N+](=O)[O-])F)C(=O)OC	Non-Carcinogen
751	Parathion	56-38-2	CCOP(=S)(OCC)OC1=CC=C(C=C1)[N+](=O)[O-]	Non-Carcinogen
752	Patulin	149-29-1	C1C=C2C(=CC(=O)O2)C(O1)O	Non-Carcinogen
753	Penicillin VK	132-98-9	CC1([C@@H](N2[C@H](S1)[C@@H](C2=O)NC(=O)COC3=CC=CC=C3)C(=O)[O-])C	Non-Carcinogen
754	Pentachloroanisole	1825-21-4	COC1=C(C(=C(C(=C1Cl)Cl)Cl)Cl)Cl	Carcinogen
755	Pentachloronitrobenzene	82-68-8	C1(=C(C(=C(C(=C1Cl)Cl)Cl)Cl)Cl)[N+](=O)[O-]	Non-Carcinogen
756	Pentaerythritol tetranitrate	78-11-5	C(C(CO[N+](=O)[O-])(CO[N+](=O)[O-])CO[N+](=O)[O-])O[N+](=O)[O-]	Non-Carcinogen
757	N-Pentyl-N'-nitro-N-nitrosoguanidine	13010-10-1	CCCCCN(/C(=N/[N+](=O)[O-])/N)N=O	Non-Carcinogen
758	Perhexiline maleate	6724-53-4	C1CCC(CC1)C(CC2CCCCN2)C3CCCCC3	Non-Carcinogen
759	Petasitenine	60102-37-6	C[C@@H]1CC2([C@H](O2)C)C(=O)O[C@@H]3CCN(C/C=C(\C3=O)/COC(=O)[C@]1(C)O)C	Carcinogen
760	Phenacetin	62-44-2	CCOC1=CC=C(C=C1)NC(=O)C	Carcinogen
761	Phenazone	60-80-0	CC1=CC(=O)N(N1C)C2=CC=CC=C2	Carcinogen
762	Phenazopyridine	136-40-3	C1=CC=C(C=C1)N=NC2=C(N=C(C=C2)N)N	Carcinogen
763	Phenesterin	3546-10-9	C[C@H](CCCC(C)C)[C@H]1CC[C@@H]2[C@@]1(CC[C@H]3[C@H]2CC=C4[C@@]3(CC[C@@H](C4)OC(=O)CC5=CC=C(C=C5)N(CCCl)CCCl)C)C	Carcinogen

764	Phenethyl isothiocyanate	2257-09-2	C1=CC=C(C=C1)CCN=C=S	Non-Carcinogen
765	Phenformin	834-28-6	C1=CC=C(C=C1)CCN=C(N)N=C(N)N	Non-Carcinogen
766	Phenobarbital	50-06-6	CCC1(C(=O)NC(=O)NC1=O)C2=CC=CC=C2	Non-Carcinogen
767	Phenobarbital	57-30-7	CCC1(C(=O)NC(=NC1=O)[O-])C2=CC=CC=C2	Carcinogen
768	Phenol	108-95-2	C1=CC=C(C=C1)O	Non-Carcinogen
769	Phenolphthalein	77-09-8	C1=CC=C2C(=C1)C(=O)OC2(C3=CC=C(C=C3)O)C4=CC=C(C=C4)O	Carcinogen
770	Phenoxybenzamine	63-92-3	CC(COC1=CC=CC=C1)N(CCCl)CC2=CC=CC=C2	Carcinogen
771	1-Phenyl-3,3-dimethyltriazene	7227-91-0	CN(C)N=NC1=CC=CC=C1	Carcinogen
772	1-Phenyl-3-methyl-5-pyrazolone	89-25-8	CC1=NN(C(=O)C1)C2=CC=CC=C2	Non-Carcinogen
773	Phenyl-beta-naphthylamine	135-88-6	C1=CC=C(C=C1)NC2=CC3=CC=CC=C3C=C2	Non-Carcinogen
774	N-Phenyl-p-phenylenediamine	2198-59-6	C1=CC=C(C=C1)NC2=CC=C(C=C2)N	Non-Carcinogen
775	2-Phenyl-1,3-propanediol dicarbamate	25451-15-4	C1=CC=C(C=C1)C(COC(=O)N)COC(=O)N	Carcinogen
776	(E)-7-Phenyl-7-(3-pyridyl)-6-heptenoic acid	89667-40-3	C1=CC=C(C=C1)/C(=C\CCCCC(=O)O)/C2=CN=CC=C2	Non-Carcinogen
777	1-Phenyl-2-thiourea	103-85-5	C1=CC=C(C=C1)NC(=S)N	Non-Carcinogen
778	1-Phenylazo-2-naphthol	842-07-9	C1=CC=C(C=C1)N/N=C/2\C(=O)C=CC3=CC=CC=C32	Carcinogen
779	Phenylbutazone	50-33-9	CCCCC1C(=O)N(N(C1=O)C2=CC=CC=C2)C3=CC=CC=C3	Carcinogen
780	p-Phenylenediamine	106-50-3	C1=CC(=CC=C1N)N	Non-Carcinogen
781	m-Phenylenediamine	541-69-5	C1=CC(=CC(=C1)N)N	Non-Carcinogen
782	o-Phenylenediamine	615-28-1	C1=CC=C(C(=C1)N)N	Carcinogen
783	Phenylephrine	61-76-7	CNC[C@@H](C1=CC(=CC=C1)O)O	Non-Carcinogen
784	Phenylethyl-3-methylcaffeate	71835-85-3	COC1=C(C=CC(=C1)/C=C/C(=O)OCCC2=CC=CC=C2)O	Non-Carcinogen
785	Phenylglycidyl ether	122-60-1	C1C(O1)COC2=CC=CC=C2	Carcinogen
786	6-Phenylhexyl isothiocyanate	133920-06-6	C1=CC=C(C=C1)CCCCCCN=C=S	Non-Carcinogen
787	o-Phenylphenol	90-43-7	C1=CC=C(C=C1)C2=CC=CC=C2O	Carcinogen

788	o-Phenylphenate	132-27-4	C1=CC=C(C=C1)C2=CC=CC=C2[O-]	Carcinogen
789	3-Phenylpropyl isothiocynate	2627-27-2	C1=CC=C(C=C1)CCCN=C=S	Non-Carcinogen
790	PhIP	NOCAS	CN1C2=C(N=CC(=C2)C3=CC=CC=C3)N=C1N	Carcinogen
791	Phthalamide	88-96-0	C1=CC=C(C(=C1)C(=O)N)C(=O)N	Non-Carcinogen
792	Phthalic anhydride	85-44-9	C1=CC=C2C(=C1)C(=O)OC2=O	Non-Carcinogen
793	Picloram	1918-02-1	C1(=C(C(=NC(=C1Cl)Cl)C(=O)O)Cl)N	Non-Carcinogen
794	Pilocarpine	92-13-7	CC[C@H]1[C@H](COC1=O)CC2=CN=CN2C	Non-Carcinogen
795	Pimaricin	7681-93-8	C[C@@H]1C/C=C/C=C/C=C/C=C/[C@@H](C[C@H]2[C@@H]([C@H](C[C@](O2)(C[C@H](C[C@@H]3[C@H](O3)/C=C/C(=O)O1)O)O)O)C(=O)O)O[C@H]4[C@H]([C@H]([C@@H]([C@H](O4)C)O)N)O	Non-Carcinogen
796	Piperazine	110-85-0	C1CNCCN1	Non-Carcinogen
797	Piperidine	110-89-4	C1CCNCC1	Non-Carcinogen
798	Piperonyl butoxide	51-03-6	CCCCOCCOCCOCC1=CC2=C(C=C1CCC)OCO2	Carcinogen
799	Piperonyl sulfoxide	120-62-7	CCCCCCCCS(=O)C(C)CC1=CC2=C(C=C1)OCO2	Non-Carcinogen
800	Pirmenol	61477-94-9	C[C@@H]1CCC[C@@H](N1CCCC(C2=CC=CC=C2)(C3=CC=CC=N3)O)C	Non-Carcinogen
801	Piroxicam	36322-90-4	CN1C(=C(C2=CC=CC=C2S1(=O)=O)O)C(=O)NC3=CC=CC=N3	Non-Carcinogen
802	Pivalolactone	1955-45-9	CC1(COC1=O)C	Carcinogen
803	2,2',4,4',5,5'-Hexabromobiphenyl	67774-32-7	C1=C(C(=CC(=C1Br)Br)Br)C2=CC(=C(C=C2Br)Br)Br	Carcinogen
804	Polybrominated biphenyls	59536-65-1	C1=CC(=C(C(=C1C2=CC(=C(C(=C2)Br)Br)Br)Br)Br)Br	Non-Carcinogen
805	2-[2-(4-Nonylphenoxy)ethoxy]ethanol	9016-45-9	CCCCCCCCCC1=CC=C(C=C1)OCCOCCO	Non-Carcinogen
806	Polysorbate 80	9005-65-6	CCCCCCCC/C=C/CCCCCCCC(=O)OCCOCC(C1C(CC(O1)OCCO)OCCO)OCCO	Non-Carcinogen
807	Polyvinylpyridine-N-oxide	9045-81-2	C=CC1=CC=CC=[N+]1[O-]	Non-Carcinogen
808	Practolol	6673-35-4	CC(C)NCC(COC1=CC=C(C=C1)NC(=O)C)O	Non-Carcinogen
809	Pranlukast	150821-03-7	C1=CC=C(C=C1)CCCCOC2=CC=C(C=C2)C(=O)NC3=CC=CC4=C3OC(=CC4=O)C5=NNN=N5	Non-Carcinogen
810	Prazepam	2955-38-6	C1CC1CN2C(=O)CN=C(C3=C2C=CC(=C3)Cl)C4=CC=CC=C4	Non-Carcinogen
811	Praziquantel	55268-74-1	C1CCC(CC1)C(=O)N2CC3C4=CC=CC=C4CCN3C(=O)C2	Non-Carcinogen
812	Prednimustine	29069-24-7	C[C@]12C[C@@H]([C@H]3[C@H]([C@@H]1CC[C@@]2(C(=O)COC(=O)CCCC4=CC=C(C=C4)	Carcinogen

			N(CCCl)CCCl)O)CCC5=CC(=O)C=C[C@]35C)O	
813	Prednisolone	50-24-8	C[C@]12C[C@@H]([C@H]3[C@H]([C@@H]1CC[C@@]2(C(=O)CO)O)CCC4=CC(=O)C=C[C@]34C)O	Carcinogen
814	Premarin	12126-59-9	C[C@]12CC[C@H]3C(=CCC4=C3C=CC(=C4)OS(=O)(=O)[O-])[C@@H]1CCC2=O	Non-Carcinogen
815	Primidone	125-33-7	CCC1(C(=O)NCNC1=O)C2=CC=CC=C2	Non-Carcinogen
816	Probenecid	57-66-9	CCCN(CCC)S(=O)(=O)C1=CC=C(C=C1)C(=O)O	Non-Carcinogen
817	Procarbazine	671-16-9	CC(C)NC(=O)C1=CC=C(C=C1)CNNC	Carcinogen
818	Procarbazine	366-70-1	CC(CN1C2=CC=CC=C2SC3=CC=CC=C31)N(C)C	Non-Carcinogen
819	Propane sultone	1120-71-4	C1COS(=O)(=O)C1	Carcinogen
820	beta-Propiolactone	57-57-8	C1COC1=O	Carcinogen
821	Propranolol	318-98-9	CC(C)NCC(COC1=CC=CC2=CC=CC=C21)O	Non-Carcinogen
822	Propyl gallate	121-79-9	CCCOC(=O)C1=CC(=C(C(=C1)O)O)O	Non-Carcinogen
823	N-Propyl-N'-nitro-N-nitrosoguanidine	13010-07-6	CCCN(/C(=N/[N+](=O)[O-])/N)N=O	Carcinogen
824	N-Propyl-N-nitrosourea	816-57-9	CCCN(C(=O)N)N=O	Carcinogen
825	Propylene	115-07-1	CC(CO)O	Non-Carcinogen
826	Propylene glycol mono-t-butyl ether	57018-52-7	CC(COC(C)(C)C)O	Non-Carcinogen
827	1,2-Propylene oxide	75-56-9	CC1CO1	Carcinogen
828	Propylthiouracil	51-52-5	CCCC1=CC(=O)NC(=S)N1	Carcinogen
829	Proresid	1508-45-8	CCNNC(=O)[C@H]1[C@@H]([C@H](C2=CC3=C(C=C2[C@H]1C4=CC(=C(C(=C4)OC)OC)OC)OCO3)O)CO	Non-Carcinogen
830	Protocatechuic acid	99-50-3	C1=CC(=C(C=C1C(=O)O)O)O	Non-Carcinogen
831	SX purple	2611-82-7	C1=CC=C2C(=C1)C(=CC=C2S(=O)(=O)[O-])N/N=C/3\C(=O)C=CC4=CC(=CC(=C43)S(=O)(=O)[O-])S(=O)(=O)[O-]	Carcinogen
832	Purpurin	81-54-9	C1=CC=C2C(=C1)C(=O)C3=C(C2=O)C(=C(C=C3O)O)O	Carcinogen
833	Pyrazinamide	98-96-4	C1=CN=C(C=N1)C(=O)N	Non-Carcinogen
834	Pyridine	110-86-1	C1=CC=NC=C1	Carcinogen
835	Pyrilamine	59-33-6	CN(C)CCN(CC1=CC=C(C=C1)OC)C2=CC=CC=N2	Carcinogen
836	Pyrimethamine	58-14-0	CCC1=C(C(=NC(=N1)N)N)C2=CC=C(C=C2)Cl	Non-Carcinogen
837	Quinapril	82586-55-8	CCOC(=O)[C@H](CCC1=CC=CC=C1)N[C@@H](C)C(=O)N2CC3=CC=CC=C3C[C@H]2C(=O)O	Non-Carcinogen
838	p-Quinone dioxime	105-11-3	C1=CC(=CC=C1NO)N=O	Carcinogen

839	Ramosetron	132907-72-3	CN1C=C(C2=CC=CC=C21)C(=O)[C@@H]3CCC4=C(C3)NC=N4	Non-Carcinogen
840	C.I. acid red 114	6459-94-5	CC1=CC=C(C=C1)S(=O)(=O)OC2=CC=C(C=C2)N=NC3=C(C=C(C=C3)C4=CC(=C(C=C4)N/N=C/5\C(=O)C=CC6=CC(=CC(=C65)S(=O)(=O)[O-])S(=O)(=O)[O-])C)C	Carcinogen
841	C.I. food red 3	3567-69-9	C1=CC=C2C(=C1)C(=CC=C2S(=O)(=O)[O-])N/N=C\3/C=C(C4=CC=CC=C4C3=O)S(=O)(=O)[O-]	Non-Carcinogen
842	C.I. pigment red 3	2425-85-6	CC1=CC(=C(C=C1)N/N=C/2\C(=O)C=CC3=CC=CC=C32)[N+](=O)[O-]	Carcinogen
843	C.I. pigment red 23	6471-49-4	COC1=C(C=C(C=C1)[N+](=O)[O-])N/N=C/2\C3=CC=CC=C3C=C(C2=O)C(=O)NC4=CC(=CC=C4)[N+](=O)[O-]	Non-Carcinogen
844	D & C red no. 5	3761-53-3	CC1=CC(=C(C=C1)N/N=C\2/C3=C(C=CC(=C3)S(=O)(=O)[O-])C=C(C2=O)S(=O)(=O)[O-])C	Carcinogen
845	D & C red no. 10	1248-18-6	C1=CC=C2C(=C1)C=CC(=C2S(=O)(=O)[O-])N/N=C/3\C(=O)C=CC4=CC=CC=C43	Non-Carcinogen
846	FD & C red no. 1	3564-09-8	CC1=CC(=C(C=C1C)N/N=C\2/C3=C(C=C(C=C3)S(=O)(=O)[O-])C=C(C2=O)S(=O)(=O)[O-])C	Carcinogen
847	FD & C red no. 2	915-67-3	C1=CC=C2C(=C1)C(=CC=C2S(=O)(=O)[O-])N/N=C\3/C4=C(C=C(C=C4)S(=O)(=O)[O-])C=C(C3=O)S(=O)(=O)[O-]	Carcinogen
848	FD & C red no. 3	16423-68-0	C1=CC=C(C(=C1)C2=C3C=C(C(=O)C(=C3OC4=C(C(=C(C=C24)I)[O-])I)I)I)C(=O)[O-]	Non-Carcinogen
849	FD & C red no. 4	4548-53-2	CC1=CC(=C(C=C1N/N=C/2\C=C(C3=CC=CC=C3C2=O)S(=O)(=O)[O-])S(=O)(=O)[O-])C	Carcinogen
850	Food red no. 106	3520-42-1	CCN(CC)C1=C(C=C2C(=C1)OC3=CC(=[N+](CC)CC)C(=CC3=C2C4=C(C=C(C=C4)S(=O)(=O)[O-])S(=O)(=O)[O-])C)C	Non-Carcinogen
851	HC red no. 3	2871-01-4	C1=CC(=C(C=C1N)[N+](=O)[O-])NCCO	Non-Carcinogen
852	Reserpine	50-55-5	CO[C@H]1[C@@H](C[C@@H]2CN3CCC4=C([C@H]3C[C@@H]2[C@@H]1C(=O)OC)NC5=C4C=CC(=C5)OC)OC(=O)C6=CC(=C(C(=C6)OC)OC)OC	Carcinogen
853	Resorcinol	108-46-3	C1=CC(=CC(=C1)O)O	Non-Carcinogen
854	Retinoic acid	302-79-4	CC1=C(C(CCC1)(C)C)/C=C/C(=C/C=C/C(=C/C(=O)O)/C)/C	Non-Carcinogen
855	Retinol acetate	127-47-9	CC1=C(C(CCC1)(C)C)/C=C/C(=C/C=C/C(=C/COC(=O)C)/C)/C	Carcinogen
856	Retinol palmitate	79-81-2	CCCCCCCCCCCCCCCC(=O)OC/C=C(\C)/C=C/C=C(\C)/C=C/C1=C(CCCC1(C)C)C	Non-Carcinogen
857	Retrorsine	480-54-6	C/C=C\1/C[C@H]([C@@](C(=O)OCC2=CCN3[C	Carcinogen

			@H]2[C@@H](CC3)OC1=O)(CO)O)C	
858	Rhodamine 6G	989-38-8	CCNC1=C(C=C2C(=C1)OC3=CC(=NCC)C(=CC3=C2C4=CC=CC=C4C(=O)OCC)C)C	Non-Carcinogen
859	Riddelliine	23246-96-0	C/C=C\1/CC(=C)[C@@](C(=O)OCC2=CCN3[C@H]2[C@@H](CC3)OC1=O)(CO)O	Carcinogen
860	Rifampicin	13292-46-1	C[C@H]1/C=C/C=C(\C(=O)NC\2=C(C3=C(C(=C4C(=C3C(=O)/C2=C\NN5CCN(CC5)C)C)C(=O)[C@](O4)(O/C=C/[C@@H]([C@H]([C@H]([C@@H]([C@@H]([C@@H]([C@H]1O)C)O)C)OC(=O)C)C)OC)C)C)O)O)/C	Non-Carcinogen
861	Ripazepam	26308-28-1	CCN1C2=C(C(=N1)C)NC(=O)CN=C2C3=CC=CC=C3	Non-Carcinogen
862	Rosaniline	632-99-5	CC1=CC(=C(C2=CC=C(C=C2)N)C3=CC=C(C=C3)N)C=CC1=N	Non-Carcinogen
863	p-Rosaniline	569-61-9	C1=CC(=N)C=CC1=C(C2=CC=C(C=C2)N)C3=CC=C(C=C3)N	Carcinogen
864	Rotenone	83-79-4	CC(=C)[C@H]1CC2=C(O1)C=CC3=C2O[C@@H]4COC5=CC(=C(C=C5[C@@H]4C3=O)OC)OC	Non-Carcinogen
865	Rutin	153-18-4	C[C@H]1[C@@H]([C@H]([C@H]([C@@H](O1)OC[C@@H]2[C@H]([C@@H]([C@H]([C@@H](O2)OC3=C(OC4=CC(=CC(=C4C3=O)O)O)C5=CC(=C(C=C5)O)O)O)O)O)O)O)O	Non-Carcinogen
866	Saccharin	81-07-2	C1=CC=C2C(=C1)C(=O)NS2(=O)=O	Non-Carcinogen
867	Saccharin	6485-34-3	C1=CC=C2C(=C1)C(=NS2(=O)=O)[O-]	Non-Carcinogen
868	Saccharin	128-44-9	C1=CC=C2C(=C1)C(=O)[N-]S2(=O)=O	Carcinogen
869	Safrole	94-59-7	C=CCC1=CC2=C(C=C1)OCO2	Carcinogen
870	Salbutamol	18559-94-9	CC(C)(C)NCC(C1=CC(=C(C=C1)O)CO)O	Carcinogen
871	Salicylazosulfapyridine	599-79-1	C1=CC=NC(=C1)NS(=O)(=O)C2=CC=C(C=C2)N/N=C\3/C=CC(=O)C(=C3)C(=O)O	Carcinogen
872	Scopolamine	6533-68-2	CN1[C@@H]2CC(C[C@H]1[C@H]3[C@@H]2O3)OC(=O)[C@@H](CO)C4=CC=CC=C4	Non-Carcinogen
873	Senkirkine	2318-18-5	C/C=C\1/C[C@H]([C@@](C(=O)OC/C/2=C/CN(CC[C@H](C2=O)OC1=O)C)(C)O)C	Carcinogen
874	Sertraline	79559-97-0	CN[C@H]1CC[C@H](C2=CC=CC=C12)C3=CC(=C(C=C3)Cl)Cl	Non-Carcinogen
875	Sesamol	533-31-3	C1OC2=C(O1)C=C(C=C2)O	Carcinogen
876	Sodium diethyldithiocarbamate	20624-25-3	CCN(CC)C(=S)[S-]	Non-Carcinogen
877	Sorbic acid	110-44-1	C/C=C/C=C/C(=O)O	Non-Carcinogen
878	Sotalol	959-24-0	CC(C)NCC(C1=CC=C(C=C1)NS(=O)(=O)C)O	Non-Carcinogen
879	Sterigmatocystin	10048-13-2	COC1=C2C(=C3C4C=COC4OC3=C1)OC5=C(C2=	Carcinogen

			O)C(=CC=C5)O	
880	Stevioside	57817-89-7	C[C@@]12CCC[C@@]([C@H]1CC[C@]34[C@H]2CC[C@](C3)(C(=C)C4)O[C@H]5[C@@H]([C@H]([C@@H]([C@H](O5)CO)O)O)O[C@H]6[C@@H]([C@H]([C@@H]([C@H](O6)CO)O)O)O)(C)C(=O)O[C@H]7[C@@H]([C@H]([C@@H]([C@H](O7)CO)O)O)O	Non-Carcinogen
881	Streptozotocin	18883-66-4	CN(C(=O)N[C@@H]1[C@H]([C@@H]([C@H](O[C@@H]1O)CO)O)O)N=O	Carcinogen
882	Styrene	100-42-5	C=CC1=CC=CC=C1	Carcinogen
883	trans-beta-Nitrostyrene	102-96-5	C1=CC=C(C=C1)/C=C/[N+](=O)[O-]	Non-Carcinogen
884	Styrene oxide	96-09-3	C1C(O1)C2=CC=CC=C2	Carcinogen
885	Succinic anhydride	108-30-5	C1CC(=O)OC1=O	Non-Carcinogen
886	Sucrose acetate isobutyrate	126-13-6	CC(C)C(=O)OC[C@@H]1[C@H]([C@@H]([C@](O1)(COC(=O)C)O[C@@H]2[C@@H]([C@H]([C@@H]([C@H](O2)COC(=O)C)OC(=O)C(C)C)OC(=O)C(C)C)OC(=O)C(C)C)OC(=O)C(C)C)OC(=O)C(C)C	Non-Carcinogen
887	Sulfallate	95-06-7	CCN(CC)C(=S)SCC(=C)Cl	Carcinogen
888	Sulfisoxazole	127-69-5	CC1=C(ON=C1C)NS(=O)(=O)C2=CC=C(C=C2)N	Non-Carcinogen
889	3-Sulfolene	77-79-2	C1C=CCS1(=O)=O	Non-Carcinogen
890	4,4'-Sulfonylbisacetanilide	77-46-3	CC(=O)NC1=CC=C(C=C1)S(=O)(=O)C2=CC=C(C=C2)NC(=O)C	Carcinogen
891	Sulindac sulfone	59973-80-7	CC\1=C(C2=C(/C1=C\C3=CC=C(C=C3)S(=O)(=O)C)C=CC(=C2)F)CC(=O)O	Non-Carcinogen
892	Suxibuzone	27470-51-5	CCCCC1(C(=O)N(N(C1=O)C2=CC=CC=C2)C3=CC=CC=C3)COC(=O)CCC(=O)O	Non-Carcinogen
893	Symphytine	22571-95-5	C/C=C(\C)/C(=O)O[C@@H]1CCN2[C@@H]1C(=CC2)COC(=O)C(C(C)C)(C(C)O)O	Carcinogen
894	Tace	569-57-3	COC1=CC=C(C=C1)C(=C(C2=CC=C(C=C2)OC)Cl)C3=CC=C(C=C3)OC	Non-Carcinogen
895	Taltirelin	NOCAS	CN1C(=O)C[C@H](NC1=O)C(=O)N[C@@H](CC2=CN=CN2)C(=O)N3CCC[C@H]3C(=O)N	Non-Carcinogen
896	Tamoxifen	54965-24-1	CC/C(=C(\C1=CC=CC=C1)/C2=CC=C(C=C2)OCCN(C)C)/C3=CC=CC=C3	Carcinogen
897	Tegafur	37076-68-9	C1CC(OC1)N2C=C(C(=O)NC2=O)F	Non-Carcinogen
898	Terbutaline	23031-25-6	CC(C)(C)NCC(C1=CC(=CC(=C1)O)O)O	Carcinogen
899	3,3',4,4'-Tetraaminobiphenyl	7411-49-6	C1=CC(=C(C=C1C2=CC(=C(C=C2)N)N)N)N	Carcinogen
900	2,3,5,6-Tetrachloro-4-nitroanisole	2438-88-2	COC1=C(C(=C(C(=C1Cl)Cl)[N+](=O)[O-])Cl)Cl	Non-Carcinogen

901	2,2',5,5'-Tetrachlorobenzidine	15721-02-5	C1=C(C(=CC(=C1Cl)N)Cl)C2=CC(=C(C=C2Cl)N)Cl	Non-Carcinogen
902	2,3,7,8-Tetrachlorodibenzo-p-dioxin	1746-01-6	C1=C2C(=CC(=C1Cl)Cl)OC3=CC(=C(C=C3O2)Cl)Cl	Carcinogen
903	Tetracycline	64-75-5	C[C@@]1([C@H]2C[C@H]3[C@@H](C(=O)C(=C([C@]3(C(=O)C2=C(C4=C1C=CC=C4O)O)O)O)C(=O)N)N(C)C)O	Non-Carcinogen
904	Tetraethylthiuram disulfide	97-77-8	CCN(CC)C(=S)SSC(=S)N(CC)CC	Non-Carcinogen
905	Tetrafluoro-m-phenylenediamine	63886-77-1	C1(=C(C(=C(C(=C1F)F)F)N)F)N	Non-Carcinogen
906	Tetrahydro-2-nitroso-2H-1,2-oxazine	40548-68-3	C1CCON(C1)N=O	Carcinogen
907	1-trans-delta9-Tetrahydrocannabinol	1972-08-3	CCCCCC1=CC2=C([C@@H]3C=C(CC[C@H]3C(O2)(C)C)C)C(=C1)O	Non-Carcinogen
908	Tetrahydrofuran	109-99-9	C1CCOC1	Carcinogen
909	3,4,5,6-Tetrahydrouridine	18771-50-1	C1CN(C(=O)NC1=O)[C@H]2[C@@H]([C@@H]([C@H](O2)CO)O)O	Non-Carcinogen
910	Tetramethylthiuram disulfide	137-26-8	CN(C)C(=S)SSC(=S)N(C)C	Non-Carcinogen
911	Thenyldiamine	91-79-2	CN(C)CCN(CC1=CSC=C1)C2=CC=CC=N2	Non-Carcinogen
912	Theophylline	58-55-9	CN1C2=C(C(=O)N(C1=O)C)NC=N2	Non-Carcinogen
913	Thiabendazole	148-79-8	C1=CC=C2C(=C1)NC(=N2)C3=CSC=N3	Non-Carcinogen
914	Thiamphenicol	15318-45-3	CS(=O)(=O)C1=CC=C(C=C1)[C@H]([C@@H](CO)NC(=O)C(Cl)Cl)O	Non-Carcinogen
915	Thio-TEPA	52-24-4	C1CN1P(=S)(N2CC2)N3CC3	Carcinogen
916	4,4'-Thiobis(6-tert-butyl-m-cresol)	96-69-5	CC1=CC(=C(C=C1SC2=CC(=C(C=C2C)O)C(C)(C)C)C(C)(C)C)O	Non-Carcinogen
917	4,4'-Thiodianiline	139-65-1	C1=CC(=CC=C1N)SC2=CC=C(C=C2)N	Carcinogen
918	beta-Thioguanine deoxyriboside	789-61-7	C1[C@@H]([C@H](O[C@H]1N2C=NC3=C2NC(=NC3=S)N)CO)O	Carcinogen
919	Thiouracil	141-90-2	C1=CNC(=S)NC1=O	Carcinogen
920	Tilidine fumarate	55567-81-2	CCOC(=O)[C@@]1(CCC=C[C@H]1N(C)C)C2=CC=CC=C2	Non-Carcinogen
921	Tilisolol	62774-96-3	CC(C)(C)NCC(COC1=CN(C(=O)C2=CC=CC=C21)C)O	Non-Carcinogen
922	dl-alpha-Tocopherol	10191-41-0	CC1=C(C(=C2CCC(OC2=C1C)(C)CCCC(C)CCCC(C)CCCC(C)C)C)O	Non-Carcinogen
923	dl-alpha-Tocopheryl acetate	7695-91-2	CC1=C2C(=C(C(=C1C)OC(=O)C)C)CCC(O2)(C)CCCC(C)CCCC(C)CCCC(C)C	Non-Carcinogen
924	Tolazamide	1156-19-0	CC1=CC=C(C=C1)S(=O)(=O)NC(=O)NN2CCCCCC2	Non-Carcinogen

925	Tolbutamide	64-77-7	CCCCNC(=O)NS(=O)(=O)C1=CC=C(C=C1)C	Non-Carcinogen
926	Toluene	108-88-3	CC1=CC=CC=C1	Carcinogen
927	Toluene diisocyanate	26471-62-5	CC1=C(C=C(C=C1)N=C=O)N=C=O	Carcinogen
928	o-Toluenesulfonamide	88-19-7	CC1=CC=CC=C1S(=O)(=O)N	Carcinogen
929	m-Toluidine	638-03-9	CC1=CC(=CC=C1)N	Non-Carcinogen
930	o-Toluidine	636-21-5	CC1=CC=CC=C1N	Carcinogen
931	p-Toluidine	540-23-8	CC1=CC=C(C=C1)N	Non-Carcinogen
932	p-Tolylurea	622-51-5	CC1=CC=C(C=C1)NC(=O)N	Non-Carcinogen
933	Toremifene citrate	89778-27-8	CN(C)CCOC1=CC=C(C=C1)/C(=C(/CCCl)\C2=CC=CC=C2)/C3=CC=CC=C3	Non-Carcinogen
934	Toxaphene	8001-35-2	C=C1C(C2(C(C(C1(C2(Cl)Cl)Cl)Cl)Cl)Cl)(CCl)CCl	Non-Carcinogen
935	Trenimon	68-76-8	C1CN1C2=CC(=O)C(=C(C2=O)N3CC3)N4CC4	Carcinogen
936	Triamcinolone acetonide	76-25-5	C[C@]12C[C@@H]([C@]3([C@H]([C@@H]1C[C@@H]4[C@]2(OC(O4)(C)C)C(=O)CO)CCC5=CC(=O)C=C[C@@]53C)F)O	Carcinogen
937	Triamterene	396-01-0	C1=CC=C(C=C1)C2=NC3=C(N=C2N)N=C(N=C3N)N	Non-Carcinogen
938	Tributyl phosphate	126-73-8	CCCCOP(=O)(OCCCC)OCCCC	Carcinogen
939	Tricaprylin	538-23-8	CCCCCCCC(=O)OCC(COC(=O)CCCCCCC)OC(=O)CCCCCCC	Carcinogen
940	2,4,6-Trichloroaniline	634-93-5	C1=C(C=C(C(=C1Cl)N)Cl)Cl	Non-Carcinogen
941	N-(Trichloromethylthio)phthalimide	133-07-3	C1=CC=C2C(=C1)C(=O)N(C2=O)SC(Cl)(Cl)Cl	Non-Carcinogen
942	2,4,6-Trichlorophenol	88-06-2	C1=C(C=C(C(=C1Cl)O)Cl)Cl	Carcinogen
943	2,4,5-Trichlorophenoxyacetic acid	93-76-5	C1=C(C(=CC(=C1Cl)Cl)Cl)OCC(=O)O	Non-Carcinogen
944	Trichlorophone	52-68-6	COP(=O)(C(C(Cl)(Cl)Cl)O)OC	Non-Carcinogen
945	1,2,3-Trichloropropane	96-18-4	C(C(CCl)Cl)Cl	Carcinogen
946	Tricresyl phosphate	1330-78-5	CC1=CC=C(C=C1)OP(=O)(OC2=CC=C(C=C2)C)OC3=CC=C(C=C3)C	Non-Carcinogen
947	Triethanolamine	102-71-6	C(CO)N(CCO)CCO	Non-Carcinogen
948	Triethylene glycol	112-27-6	C(COCCOCCO)O	Non-Carcinogen
949	2,2,2-Trifluoro-N-[4-(5-nitro-2-furyl)-2-thiazolyl]acetamide	42011-48-3	C1=C(OC(=C1)[N+](=O)[O-])C2=CSC(=N2)NC(=O)C(F)(F)F	Carcinogen

950	Trifluralin	1582-09-8	CCCN(CCC)C1=C(C=C(C=C1[N+](=O)[O-])C(F)(F)F)[N+](=O)[O-]	Non-Carcinogen
951	Trimethadione	127-48-0	CC1(C(=O)N(C(=O)O1)C)C	Non-Carcinogen
952	(+-)-7-(3,5,6-trimethyl-1,4-benzoquinon-2-yl)-7-phenylheptanoic acid	112665-43-7	CC1=C(C(=O)C(=C(C1=O)C)C(CCCCCC(=O)O)C2=CC=CC=C2)C	Non-Carcinogen
953	2,4,5-Trimethylaniline	137-17-7	CC1=CC(=C(C=C1C)N)C	Carcinogen
954	2,4,5-Trimethylaniline	21436-97-5	CC1=CC(=C(C(=C1)C)N)C	Carcinogen
955	1,2,4-Trimethylbenzene	95-63-6	CC1=CC(=C(C=C1)C)C	Carcinogen
956	Trimethylphosphate	512-56-1	COP(=O)(OC)OC	Non-Carcinogen
957	Trimethylthiourea	2489-77-2	CNC(=S)N(C)C	Carcinogen
958	1,3,5-Trinitrobenzene	99-35-4	C1=C(C=C(C=C1[N+](=O)[O-])[N+](=O)[O-])[N+](=O)[O-]	Non-Carcinogen
959	Trinitroglycerin	55-63-0	C(C(CO[N+](=O)[O-])O[N+](=O)[O-])O[N+](=O)[O-]	Carcinogen
960	Triphenyltin hydroxide	76-87-9	C1=CC=C(C=C1)[Sn](C2=CC=CC=C2)C3=CC=CC=C3	Non-Carcinogen
961	Triprolidine monohydrate	6138-79-0	CC1=CC=C(C=C1)/C(=C\CN2CCCC2)/C3=CC=CC=N3	Non-Carcinogen
962	Tris(2-chloroethyl) phosphate	115-96-8	C(CCl)OP(=O)(OCCCl)OCCCl	Carcinogen
963	Tris(2,3-dibromopropyl)phosphate	126-72-7	C(C(CBr)Br)OP(=O)(OCC(CBr)Br)OCC(CBr)Br	Carcinogen
964	Tris-(1,3-dichloro-2-propyl)phosphate	13674-87-8	C(C(CCl)OP(=O)(OC(CCl)CCl)OC(CCl)CCl)Cl	Carcinogen
965	Tris(2-ethylhexyl)phosphate	78-42-2	CCCCC(CC)COP(=O)(OCC(CC)CCCC)OCC(CC)CCCC	Non-Carcinogen
966	Tris(2-hydroxypropyl)amine	122-20-3	CC(CN(CC(C)O)CC(C)O)O	Non-Carcinogen
967	N-Tritriacontane-16-18-dione	24514-86-1	CCCCCCCCCCCCCCCC(=O)CC(=O)CCCCCCCCCCCCCCC	Non-Carcinogen
968	Trp-P-1 acetate	75104-43-7	CC1=C(N=C(C2=C1NC3=CC=CC=C32)C)N	Carcinogen
969	Trp-P-2 acetate	72254-58-1	CC1=C2C3=CC=CC=C3NC2=CC(=N1)N	Carcinogen
970	dl-Tryptophan	54-12-6	C1=CC=C2C(=C1)C(=CN2)CC(C(=O)O)N	Non-Carcinogen
971	l-Tryptophan	73-22-3	C1=CC=C2C(=C1)C(=CN2)C[C@@H](C(=O)O)N	Non-Carcinogen
972	Uracil	66-22-8	C1=CNC(=O)NC1=O	Carcinogen
973	Urapidil	34661-75-1	CN1C(=CC(=O)N(C1=O)C)NCCCN2CCN(CC2)C	Non-Carcinogen

			3=CC=CC=C3OC	
974	Urethane	51-79-6	CCOC(=O)N	Carcinogen
975	Vinyl acetate	108-05-4	CC(=O)OC=C	Carcinogen
976	3-Methylstyrene	100-80-1	CC1=CC=CC(=C1)C=C	Non-Carcinogen
977	N-Vinylpyrrolidone-2	88-12-0	C=CN1CCCC1=O	Carcinogen
978	FD & C violet no. 1	1694-09-3	CCN(CC1=CC(=CC=C1)S(=O)(=O)[O-])C2=CC=C(C=C2)C(=C3C=CC(=[N+](C)C)C=C3)C4=CC=C(C=C4)N(CC)CC5=CC(=CC=C5)S(=O)(=O)[O-]	Carcinogen
979	Voglibose	83480-29-9	C1[C@@H]([C@@H]([C@H]([C@@H]([C@]1(CO)O)O)O)O)NC(CO)CO	Non-Carcinogen
980	Watanidipine	133743-71-2	CC1=C(C(C(=C(N1)C)C(=O)OCCC2=CC=C(C=C2)N3CCN(CC3)C(C4=CC=CC=C4)C5=CC=CC=C5)C6=CC(=CC=C6)[N+](=O)[O-])C(=O)OC	Non-Carcinogen
981	Xylazine	23076-35-9	CC1=C(C(=CC=C1)C)NC2=NCCCS2	Non-Carcinogen
982	2,4-Xylidine	21436-96-4	CC1=CC(=C(C=C1)N)C	Non-Carcinogen
983	2,5-Xylidine	51786-53-9	CC1=CC(=C(C=C1)C)N	Carcinogen
984	C.I. disperse yellow 3	2832-40-8	CC1=C/C(=N/NC2=CC=C(C=C2)NC(=O)C)/C(=O)C=C1	Carcinogen
985	C.I. pigment yellow 12	6358-85-6	CC(=O)C(C(=O)NC1=CC=CC=C1)N=NC2=C(C=C(C=C2)C3=CC(=C(C=C3)N=NC(C(=O)C)C(=O)NC4=CC=CC=C4)Cl)Cl	Non-Carcinogen
986	C.I. pigment yellow 16	5979-28-2	CC1=C(C=CC(=C1)C2=CC(=C(C=C2)NC(=O)C(C(=O)C)N=NC3=C(C=C(C=C3)Cl)Cl)C)NC(=O)C(C(=O)C)N=NC4=C(C=C(C=C4)Cl)Cl	Non-Carcinogen
987	C.I. pigment yellow 83	5567-15-7	CC(=O)C(C(=O)NC1=CC(=C(C=C1OC)Cl)OC)N=NC2=C(C=C(C=C2)C3=CC(=C(C=C3)N=NC(C(=O)C)C(=O)NC4=CC(=C(C=C4OC)Cl)OC)Cl)Cl	Non-Carcinogen
988	C.I. vat yellow 4	128-66-5	C1=CC=C2C(=C1)C3=C4C(=CC=C5C4=C(C=C3)C(=O)C6=CC=CC=C65)C2=O	Non-Carcinogen
989	FD & C yellow no. 5	1934-21-0	C1=CC(=CC=C1N/N=C\2/C(=NN(C2=O)C3=CC=C(C=C3)S(=O)(=O)[O-])C(=O)[O-])S(=O)(=O)[O-]	Non-Carcinogen
990	FD & C yellow no. 6	2783-94-0	C1=CC(=CC=C1N/N=C\2/C3=C(C=CC2=O)C=C(C=C3)S(=O)(=O)[O-])S(=O)(=O)[O-]	Non-Carcinogen
991	HC yellow 4	59820-43-8	C1=CC(=C(C=C1[N+](=O)[O-])OCCO)NCCO	Non-Carcinogen
992	Zatosetron maleate	123482-23-5	CC1(CC2=C(O1)C(=CC(=C2)Cl)C(=O)NC3C[C@H]4CCC(C3)N4C)C	Non-Carcinogen
993	Zearalenone	17924-92-4	C[C@H]1CCCC(=O)CCC/C=C/C2=CC(=CC(=C2C(=O)O1)O)O	Non-Carcinogen
994	2,2-Bis(bromomethyl)-1,3-propanediol	3296-90-0	C(C(CO)(CBr)CBr)O	Carcinogen

995	C.I. direct blue 218	28407-37-6	C1=CC(=C(C=C1C2=CC(=C(C=C2)N/N=C/3\C(=O)C4=C(C=C(C=C4C=C3S(=O)(=O)[O-])S(=O)(=O)[O-])N)O)O)N/N=C/5\C(=O)C6=C(C=C(C=C6C=C5S(=O)(=O)[O-])S(=O)(=O)[O-])N	Carcinogen
996	C.I. direct brown 95	16071-86-6	C1=CC(=CC=C1C2=CC=C(C=C2)N=NC3=CC(=C(C=C3)[O-])C(=O)[O-])N/N=C/4\C(=O)C=C/C(=N\NC5=C(C=CC(=C5)S(=O)(=O)[O-])[O-])/C4=O	Carcinogen
997	1,3-Butadiene	106-99-0	C=CC=C	Carcinogen
998	Calcium lactate	814-80-2	CC(C(=O)[O-])O	Non-Carcinogen
999	Calcium valproate	33433-82-8	CCCC(CCC)C(=O)[O-]	Carcinogen
1000	Propylene	115-07-1	CC=C	Non-Carcinogen
1001	Chlorin E6	19660-77-6	CCC1=C(C2=CC3=NC(=CC4=NC(=C(C5=NC(=C(C5=C([O-])[O-])C)C=C1[N-]2)CC(=O)[O-])[C@H]([C@@H]4C)CCC(=O)[O-])C(=C3C=C)C)C	Non-Carcinogen
1002	Tetrakis(hydroxymethyl)phosphonium chloride	124-64-1	C(O)[P+](CO)(CO)CO	Non-Carcinogen
1003	Vinblastine	865-21-4	CC[C@@]1(C[C@@H]2C[C@@](C3=C(CCN(C2)C1)C4=CC=CC=C4N3)(C5=C(C=C6C(=C5)[C@]78CCN9[C@H]7[C@@](C=CC9)([C@H]([C@@]([C@@H]8N6C)(C(=O)OC)O)OC(=O)C)CC)OC)C(=O)OC)O	Non-Carcinogen

Supplementary Table S3: Detailed information for the compounds in the external validation dataset from the ISSCAN database

Index	Name	CAS	SMILES	Class
1	2-Ethylhexyl Sulfate	126-92-1	CCCCC(CC)COS(=O)(=O)[O-]	Non-Carcinogen
2	Bis(2-chloroethyl) ether	111-44-4	ClCCOCCCl	Non-Carcinogen
3	Diethanolamine	111-42-2	OCCNCCO	Non-Carcinogen
4	Benzethonium Chloride	121-54-0	O(c1ccc(cc1)C(C)(C)CC(C)(C)C)CCOCC[N+](Cc1ccccc1)(C)C	Non-Carcinogen
5	2-tert-Butylhydroquinone	1948-33-0	Oc1c(cc(O)cc1)C(C)(C)C	Non-Carcinogen
6	Sodium Xylenesulfonate	1300-72-7	[O-]S(=O)(=O)c1cc(C)cc(C)c1	Non-Carcinogen
7	Diethyl Phthalat	84-66-2	O=C(OCC)c1c(cccc1)C(=O)OCC	Non-Carcinogen
8	Allyl Chloride	107-05-1	C=CCCl	Non-Carcinogen
9	Acifluorfen	50594-66-6	OC(=O)c1c(ccc(c1)Oc1ccc(cc1Cl)C(F)(F)F)[N+](=O)[O-]	Non-Carcinogen
10	alpha-(2,4-Dichlorophenoxy)propionic acid	120-36-5	Clc1c(ccc(c1)Cl)O[C@@H](C(=O)O)C	Non-Carcinogen
11	l-Glutamic acid	56-86-0	N[C@H](CCC(=O)O)C(=O)O	Non-Carcinogen
12	Isomalt	64519-82-0	[C@@H]1(O[C@@H]([C@H]([C@@H]([C@H]1O)O)O)CO)OC[C@H]([C@H]([C@@H]([C@@H](CO)O)O)O)O)O	Non-Carcinogen
13	p-Methylstyrene	622-97-9	C=Cc1ccc(cc1)C	Non-Carcinogen
14	dl-Monosodium glutamate	617-65-2	O=C(C(CCC(=O)O)N)O	Non-Carcinogen
15	Nitrosomethylphenidate	55557-03-4	c1ccc(C(C(=O)OC)C2N(N=O)CCCC2)cc1	Non-Carcinogen
16	Simazine	122-34-9	Clc1nc(nc(n1)NCC)NCC	Non-Carcinogen
17	Temazepam	846-50-4	CN1c2ccc(cc2C(=NC(C1=O)O)c1ccccc1)Cl	Non-Carcinogen
18	4-Nitroquinoline-N-Oxide	56-57-5	N(=O)(=O)c1cc[n+]([O-])c2ccccc12	Carcinogen
19	Methylazoxymethanol Acetate	592-62-1	CC(=O)OCN=N(=O)C	Carcinogen
20	D&C Red no. 9	1190723	Clc1cc(c(/N=N/c2c3ccccc3ccc2O)cc1C)S(=O)(=O)[O-]	Carcinogen
21	Chlornaphazine	494-03-1	ClCCN(CCCl)c1ccc2ccccc2c1	Carcinogen
22	Adriamycin	23214-92-8	O=C1c2c(O)c3c(c(O)c2C(=O)c2cccc(OC)c12)C[C@@](O)(C(=O)CO)C[C@@H]3O[C@@H]1O[C@H]([C@@H](O)[C@@H](N)C1)C	Carcinogen

23	Diethyl Sulfate	64-67-5	O=S(=O)(OCC)OCC	Carcinogen
24	Ethyl Methanesulfonate	62-50-0	O=S(=O)(OCC)C	Carcinogen
25	Methylthiouracil	56-04-2	O=C1C=C(NC(N1)=S)C	Carcinogen
26	2-Amino-3-methyl-9H-pyrido[2,3-b]indole	68006-83-7	n1c(c(cc2c3c(cccc3)[nH]c12)C)N	Carcinogen
27	N-Nitroso-2,6-dimethylmorpholine	1456-28-6	O=NN1C[C@@H](O[C@@H](C)C1)C	Carcinogen
28	9,10-Anthraquinone	84-65-1	O=C1c2c(cccc2)C(=O)c2c1cccc2	Carcinogen
29	N-nitroso-1,3-oxazolidine	39884-52-1	C1N(COC1)N=O	Carcinogen
30	D & C yellow no. 11	8003-22-3	c1ccc2nc(C3C(=O)c4ccccc4C3=O)ccc2c1	Carcinogen
31	2,3-dibromo-1-propanol	96-13-9	OCC(Br)CBr	Carcinogen
32	1,2-Dihydro-2,2,4-trimethylquinoline	147-47-7	N1c2c(C(=CC1(C)C)C)cccc2	Carcinogen
33	beta-2'-Deoxythioguanosine	789-61-7	C1[C@@H]([C@H](O[C@H]1N2C=NC3=C2NC(=NC3=S)N)CO)O	Carcinogen
34	Biphenyl	92-52-4	C1=CC=C(C=C1)C2=CC=CC=C2	Carcinogen
35	7,12-Dimethylbenz(a)anthracene	57-97-6	Cc1c2ccccc2c(c2ccc3c(c12)cccc3)C	Carcinogen
36	Isosafrole	120-58-1	C/C=C/c1cc2c(cc1)OCO2	Carcinogen
37	Mestranol	72-33-3	C[C@@]12[C@]([C@]3([C@@](c4c(cc(cc4)OC)CC3)(CC1)C)C)(CC[C@]2(O)C#C)C	Carcinogen
38	N-Nitrosomethyl-(2-tosyloxyethyl) amine	NOCAS	NC(COS(=O)(=O)c1ccc(C)cc1)CN=O	Carcinogen
39	Norethynodrel	68-23-5	C[C@@]12C(C3C(C4=C(CC(=O)CC4)CC3)CC1)CC[C@@]2(C#C)O	Carcinogen
40	N,N,N'-trimethyl-N'-nitroso-1,2-ethanediamine	23834-30-2	CN(C)CCN(C)N=O	Carcinogen
